# Differing field methods and site conditions lead to varying bias in suspended sediment concentrations in the Lower Mississippi and Atchafalaya Rivers

**DOI:** 10.1007/s10661-023-11836-z

**Published:** 2023-10-02

**Authors:** J. Murphy, L. Schafer, S. Mize

**Affiliations:** 1 US Geological Survey, DeKalb, IL USA; 2 US Geological Survey, Catonsville, MD USA; 3 US Geological Survey, Baton Rouge, LA USA

**Keywords:** Suspended sediment, Lower Mississippi/Atchafalaya River Basin, Depth-integrated, Point-integrated, Field methods, Weighted regressions on time, discharge, and season (WRTDS)

## Abstract

At sites that have been sampled for decades, changes in field and laboratory methods happen over time as instrumentation and protocols improve. Here, we compare the influence of depth- and point-integrated sampling on total, fine (< 0.0625 mm), and coarse (≥ 0.0625 mm) suspended sediment (SS) concentrations in the Lower Mississippi and Atchafalaya Rivers. Using historical field method information, we identified seven sites to test such differences. We found SS samples collected using point-integration tended to have higher concentrations than those collected using depth-integration. However, the presence and magnitude of the bias were inconsistent across sites. Bias was present at the site with less-than-ideal conditions (i.e., non-trapezoidal channel, non-uniform flow) and non-existent at the ideal site location, indicating the bias between sampling methods depends on site sampling conditions. When present, the bias is greater at higher concentrations and at moderate to high flows. At the less-than-ideal site, point-integrated samples can have 16% (total) and 34% (coarse) higher concentrations than depth-integrated samples. When flow effects are removed, this translates to a bias of 19, 9, and 8 mg per liter for total, fine, and coarse SS. When a change in field methods occurs, comparison samples and a rigorous evaluation of those samples are warranted to determine the proper course of action for a particular site. Often, the effect and solution will not be known until several years of comparison samples have been collected under a variety of hydrologic conditions.

**Supplementary Information:**

The online version contains supplementary material available at 10.1007/s10661-023-11836-z.

## Introduction

As technology advances, more precise and accurate determinations of the physical and chemical properties of water are possible due to improvements in laboratory and field instruments. However, at sites that have been sampled for decades, these laboratory and field method changes may interfere with the ability to track environmental changes over time and can lead to biased estimates of flux. For suspended sediment (SS), studies show that field grab samples and the “total suspended solids” laboratory method are biased low compared to depth-integrated sampling methods and the “suspended sediment concentration” laboratory method (Gray et al., 2000; Groten & Johnson, 2018). This bias is due to the former methods failing to adequately capture or characterize the coarse (sands) SS fraction. Identifying and rectifying SS records produced using multiple laboratory methods, field methods, or both can be challenging and would be helped by a combination of historical information about sample collection and laboratory analysis along with statistical evaluation of the data to detect differences and their magnitudes.

In the Lower Mississippi and Atchafalaya River Basin, point-integrated sampling is an additional field method used to collect SS samples, and sites in this area were typically sampled using point- or depth-integrated fields methods or sometimes both. The primary difference between point-integrated and depth-integrated sampling methods is whether the sampler stays open and collects a single integrated water sample along the entire vertical (i.e., depth-integrated) or if the sampler is only opened at specific depths, and multiple individual samples are collected along each vertical (i.e., point-integrated).

**Fig. 1:**
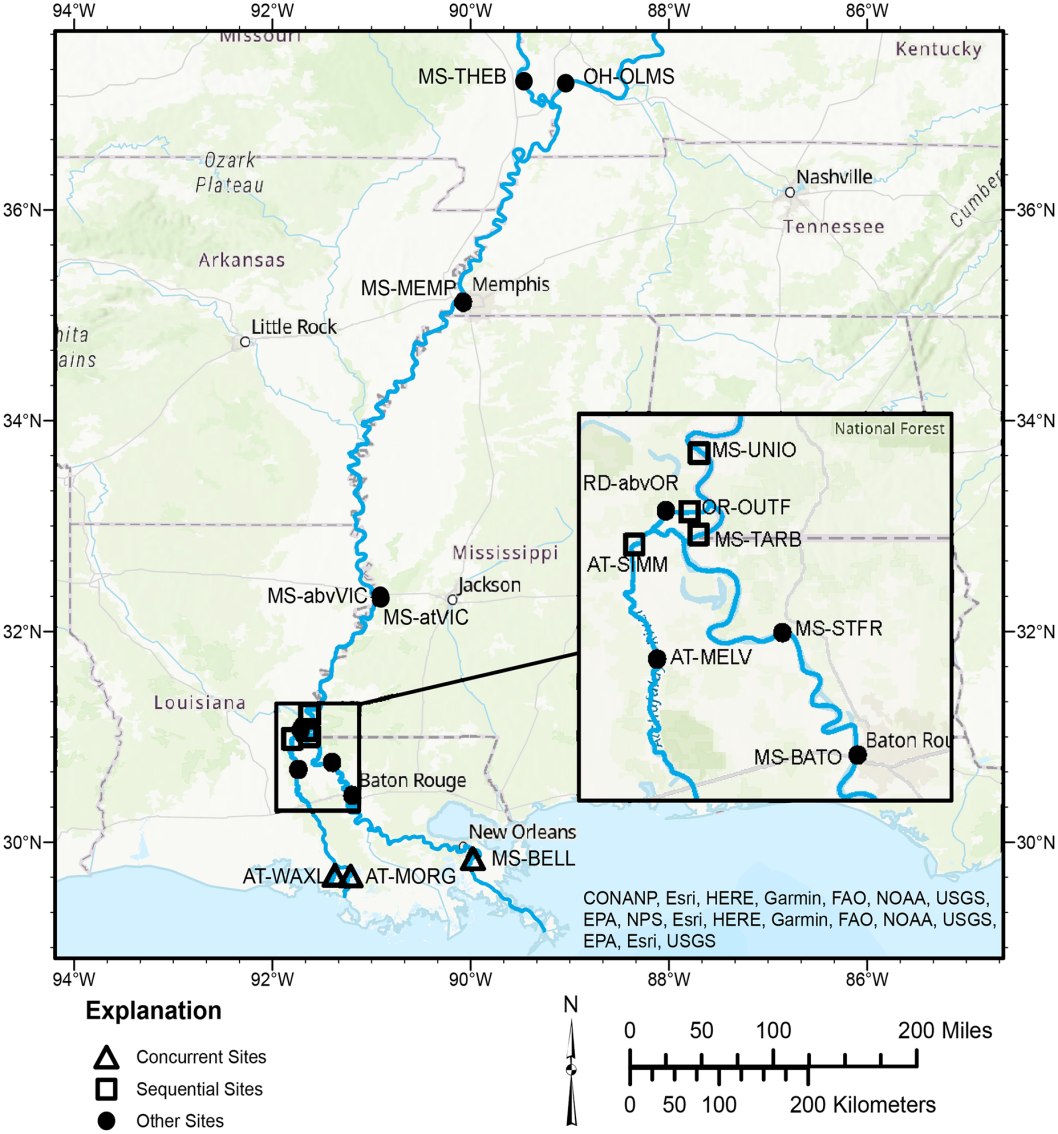
Map of Mississippi/Atchafalaya River Basin sites used in this study, including sites on the Ohio River, the Middle and Lower Mississippi River, Red River, and Atchafalaya River

To our knowledge, no one has directly compared point- and depth-integrated techniques in the field at multiple sites and across a range of hydrologic and environmental conditions. For this study, we compiled reported field method information for 16 sites in the Middle and Lower Mississippi/Atchafalaya River Basin (Fig. 1) to understand how SS field methods have evolved over time and identify datasets where differences in depth- and point-integrated sampling can be evaluated. At 7 of 16 sites, we use a variety of statistical techniques to explore the influence of depth- versus point-integrated sampling on total, fine (< 0.0625 mm diameter), and coarse (≥ 0.0625 mm diameter) SS concentrations. Statistical techniques used to test for differences include multiple linear regression (MLR) and nonparametric tests of paired and unpaired samples. At two sites with the longest records, we also evaluate effects on composited estimates, specifically annual mean concentrations, derived from a model calibrated with only point- or depth-integrated samples.

Our primary objective is to determine if there is significant bias in SS concentrations, for total, fine, or coarse SS, based on the sampling methods used to collect the sample and, if present, characterize this bias across a range of conditions. We also provide possible ways for how to address the bias if present. Ultimately, we want to determine the usability of SS data records that were collected using multiple field methods to help resource managers, scientists, and engineers working in large river systems better understand and model SS from these complex records. In the Lower Mississippi and Atchafalaya Rivers, like in many large coastal river systems, SS concentrations are used to determine SS fluxes (e.g., Allison et al., 2012) and trends (e.g., Kleiss et al., 2021; Mize et al., 2018), and accurate estimation of these values are important for maintaining adequate channel depths for navigation and for planning and implementing coastal restoration activities.

## Background

Historically, the US Army Corps of Engineers (USACE) and the US Geological Survey (USGS) used point-integrated sampling techniques at select sites on the Lower Mississippi and Atchafalaya Rivers. Like depth-integrated sampling, point-integrated sampling is used to determine the cross-sectional variability of SS concentrations for a river channel. Both sampling methods collect water samples along multiple vertical profiles across a channel cross section that are then composited to determine the SS concentration (Edwards & Glysson, 1999; Gray & Landers, 2014). For point-integrated sampling, typically four to eight verticals are used, and two to five samples are collected at specific depths ranging from 10 to 90% the stream depth for each vertical (sometimes 2 feet from the bottom of the channel). The placement of the verticals is determined based on the variation of the stream velocity across the cross section, which is determined prior to sampling. Details of some of the USACE Lower Mississippi sediment sampling program and analysis are described in Thorne et al. (2000). USGS point-integrated sampling protocols can be found in Edwards and Glysson (1999).

Historically and recently, the USGS also sampled many of these same sites using depth-integrated methods (Edwards & Glysson, 1999). Depth-integrated sampling at these sites includes equal-width increment (EWI) or the equal-discharge increment (EDI) methods. Placement of the verticals is determined by dividing the length of the channel cross section into equal widths (i.e., EWI) or by dividing the flow volume of the cross section into columns of water representing equal flow but with different widths (i.e., EDI). For each vertical, samples are collected throughout the entire water column from near surface to near bottom (e.g., 90% the stream depth or 2 feet from the channel bottom). The sampler is raised and lowered at a consistent speed (for each transect if EWI or for all transects if EDI), which is determined prior to sampling based on current stream velocities, sampler type, and nozzle size. Water for the sample is collected the entire time the sampler is raised and lowered. Davis and FISP (2005) and Edwards and Glysson (1999) describe sediment sampler types and USGS guidelines for their use.

The Federal Interagency Sedimentation Project (FISP) has been responsible for developing and testing sediment samplers for decades. One goal is the equivalency of the SS measurements collected using FISP approved samplers (Davis & FISP, 2005). However, most of the SS sampler testing has occurred in the laboratory or with a sampler towed behind a boat in calm waters, and few field tests in rivers exist (Sabol & Topping, 2013). A few studies have evaluated the performance and representativeness of depth-integrated (Sabol & Topping, 2013; Sabol et al., 2022; Topping et al., 2011) *or* point-integrated (Gitto et al., 2017) sampling techniques in the field or compared depth-integrated sampling versus other methods such as grab or pump samples (Horowitz et al., 1990).

Topping et al. (2011) provide a framework for understanding multiple sources of error during the collection of a depth-integrated sample. Although the user derived errors (e.g., incorrect transit rate) are specific to depth-integrated techniques, the non-user derived errors are equally relevant to point-integrated techniques. These include errors due to non-uniform distribution of SS concentration across the channel cross section and errors due to the limited amount of time used to compute an average concentration of a vertical section of the water column. Topping et al. (2011) found errors could be reduced approximately 30% by doubling the number of times the depth-integrated sampler was raised and lowered for a given vertical. Similar time-related errors occur for point-integrated sampling. Gitto et al. (2017) studied SS in the Frasier River in Canada and found that point-integrated techniques could not provide a representative mean SS concentration at a point because the sampler was not open long enough to provide an integrated measure of the SS variability. Horowitz et al. (1990) found statistically significant differences in SS concentrations between samplers and sampling methods when compared in rivers with high proportions of coarse SS.

At some sites in the Lower Mississippi and Atchafalaya Rivers, both point- and depth-integrated sampling methods were used over the same period of time; at other sites, sampling methods switched from point-integrated to depth-integrated sampling methods in 2019 or 2020. Most of the samples from these sites, regardless of the sampling method used to collect them, were analyzed in the USGS sediment laboratory in Baton Rouge, Louisiana, following a combination of USGS protocols from Guy (1969) and local laboratory procedures (Marlon Johnson, US Geological Survey, written communication, July 5, 2023). These data allow for an evaluation of how differences in sampling methods influence concentrations for total, fine (silt), and coarse (sand) SS concentrations in big rivers carrying large quantities of suspended material.

## Data and methods

### Study sites and data compilation

This study includes 16 large river sites located in the Mississippi/Atchafalaya River Basin (Fig. 1), including sites on the Ohio River, Middle and Lower Mississippi River, Red River, and Atchafalaya River (the Mississippi River’s largest distributary). All 16 sites have been sampled for SS for at least 10 years (Table 1). Period of records start as early as 1973 and as late as 2008, and most sites are currently being sampled (as of 2023) although sampling at two locations was discontinued in the mid-2000s (Table 1). SS sampling frequency and duration vary between sites and over time. A sampled water year typically has at least 10 samples (Online Resource 1, Fig. SI1-1). A water year is a period from October 1 to September 30 designated by the year in which it ends (e.g., water year (WY) 2020 was from October 1, 2019, to September 30, 2020).

**Table 1:** Site information, period of record (in water years; the period from October 1 to September 30 designated by the year in which it ends) for available total suspended sediment concentration data in Murphy et al. (2022). Online Resource 3 includes streamgage numbers and flow record processing steps. Latitude and longitude in decimal degrees, North American Datum of 1983

**Site (USGS site numbers)**	**Paired streamgage index**	**Site name**	**Suspended sediment period of record**	**Latitude, longitude**
MS-THEB (07022000)	MS-THEB	Mississippi River at Thebes, IL	1973–2021	37.22160, − 89.46298
OH-OLMS (03612600; 03612500)	OH-OLMSqx	Ohio River at Olmsted, IL; Ohio River at Dam 53 near Grand Chain, IL	1973–2021	37.17922, − 89.05840; 37.20311, − 89.04174
MS-MEMP (07032000)	MS-MEMPqx	Mississippi River at Memphis, TN	1989–2020	35.12315, − 90.07759
MS-abvVIC (322023090544500)	MS-abvVICqx	Mississippi River above Vicksburg at Mile 438, MS	2008–2021	32.33972, − 90.91250
MS-atVIC (07289000)	MS-atVICqx	Mississippi River at Vicksburg, MS	1973–2008	32.31500, − 90.90583
MS-UNIO (07295025)	MS-UNIOqx	Mississippi River at Union Point (Mile 326), LA	1991–2021	31.21625, − 91.62197
MS-TARB (07295100)	MS-TARB	Mississippi River at Tarbert Landing, MS	1990–2021	31.00851, − 91.62373
MS-STFR (07373420)	MS-TARB	Mississippi River near St. Francisville, LA	1978–2021	30.75852, − 91.39595
MS-BATO (07374000)	MS-BATO	Mississippi River at Baton Rouge, LA	1975–2021	30.44567, − 91.19156
MS-BELL (07374525)	MS-BELLqx	Mississippi River at Belle Chasse, LA	2006–2021	29.85715, − 89.97785
RD-abvOR (310408091424500)	RD-abvORqx	Red River above Old River Outflow Channel above Simmesport, LA	1990–2005, 2019–2022^a^	31.06907, − 91.71262
OR-OUTF (07381482)	OR-OUTF	Old River Outflow Channel below Hydropower Channel	2010–2021	31.06675, − 91.64828
AT-SIMM (07381490)	AT-SIMM	Atchafalaya River at Simmesport, LA	1990^b^–2021	30.98250, − 91.79833
AT-MELV (07381495)	AT-SIMM	Atchafalaya River at Melville, LA	1980–2021	30.69074, − 91.73623
AT-WAXL (07381590)	AT-WAXLqx	Wax Lake Outlet at Calumet, LA	1990–2021	29.69799, − 91.37289
AT-MORG (07381600)	AT-MORGqx	Lower Atchafalaya River at Morgan City, LA	1990–2021	29.69282, − 91.21194

^a^Additional samples collected approximately half a mile upstream at USGS site 07355690

^b^4–5 samples available in 1976

The Mississippi/Atchafalaya River Basin is approximately 3.2 million square kilometers and covers about 40% of the conterminous United States. The basin is highly engineered allowing for extensive use of the basin’s waterways for recreation, navigation, power generation, and flood control. These engineering features include single- and multi-purpose dams, navigations locks, levees, floodways, dikes, and revetments, along with channel straightening and deepening efforts (Alexander et al., 2012). However, the Middle and Lower Mississippi River do not have any main channel dams. Furthermore, the Mississippi River downstream from the Ohio River historically had a wide floodplain that allowed lateral migration of the channel. Over the last century, engineering structures have restrained the lateral movement of the river and encouraged channel deepening to support navigation (Alexander et al., 2012). SS has declined in the Mississippi River for many decades. The largest decreases in SS occurred prior to or around the 1980s in the Lower Mississippi and Atchafalaya Rivers (Meade & Moody, 2010; Mize et al., 2018) yet leveled off over the last decade (Kleiss et al., 2021).

The data used in this study are available in Murphy et al. (2022) and include SS concentrations and particle size fractions, field method information, and continuous records of daily streamflow. These data were originally retrieved from the USGS National Water Information System (NWIS) database (US Geological Survey, 2022). For this study, we focus on total, fine, and coarse SS sediment concentrations. We use total SS concentrations (USGS parameter code (pcode) 80154) and the percentage of suspended particles with diameters smaller than 0.0625 mm (i.e., fine SS or silt; pcode 70331) to compute concentrations of fine and coarse SS. The percentage of fine sediment was multiplied by the concentration of total SS to get the concentration of fine SS. Coarse SS concentrations (diameters ≥ 0.0625 mm) were computed as the difference between the concentrations of total and fine SS. We used R (R Core Team, 2022), RStudio (RStudio Team, 2020), and the tidyverse (Wickham et al., 2019) for data processing and analysis. Unless noted otherwise, the base R stats package was used for statistical analyses (R Core Team, 2022).

The field method information in Murphy et al. (2022) was harmonized after retrieval from NWIS, which included cleaning and combining various text fields. The available field method information includes the organization that collected the sample, sampling method (pcode 82398), type of sampler (pcode 84164), type of sample splitter (pcode 84171), nozzle material (pcode 72219), nozzle diameter (pcode 72220), material of the sampler (pcode 84182), and the number of sampling points (pcode 00063). Additionally, we compiled summary timelines of field method information for each site that combined information from NWIS with information found in internal agency documents and provided via written and verbal communication from USGS and USACE staff involved in sample collection (Online Resource 2). Information from NWIS and the summary timelines were used to identify periods when field methods or equipment changed and periods when more than one sampling method or equipment type was used concurrently. These situations provided us with datasets to test for the effect of different field methods on total, fine, and coarse SS concentrations. Murphy et al. (2022) provides additional metadata information about the compilation, cleaning, and harmonization of the field method information.

Continuous daily streamflow records from Murphy et al. (2022) were used for 7 of the 16 sites. Murphy et al. (2022) compiled these streamflow records from multiple sources. Measured values were retrieved from NWIS using the dataRetrieval package (De Cicco et al., 2021), USACE’s rivergages.com webpage, and written communication from USACE staff (James Lewis, USACE, written communication, August 31, 2021, September 1, 2021, and September 9, 2021). Days with missing values were estimated using interpolated daily streamflow ratios or a smoothed structural time series model implemented via the waterData R package (Ryberg & Vecchia, 2017). For some sites, streamflow was computed by adding or subtracting flow from upstream and/or downstream gages. Online Resource 3 provides more details about the compilation and computation of the daily streamflow records available in Murphy et al. (2022) and used in this analysis.

### Point- and depth-integrated datasets

A review of the summary field method timelines (Online Resource 2) and NWIS field method information by site and over time (Online Resources 4 and 5) revealed seven sites where differences in point- and depth-integrated sampling could be tested (Table 2). A sample was designated as “point-integrated” if the sampling method listed “composite-points” or sampler type listed “Point sampler.” A sample was designated as “depth-integrated” if the sampling method was “EDI” or “EWI,” or if sampler type listed “US D-74,” “US D-96,” or “US D-99” (Davis & FISP, 2005). The seven sites form two groups based on if the sampling methods occurred concurrently (i.e., samples were collected using both point- and depth- integrated techniques over the same sampling period) or sequentially (i.e., there was an abrupt switch from point-integrated to depth-integrated sampling). Due to differences in data density and time periods, different statistical techniques were used depending on the sites and dataset (Table 2). Also, depending on the analysis, sites were analyzed individually, in combination with other sites, or only for a subset of the data, such as for paired samples collected on the same day (Table 2).

**Table 2:** Sites, analyses, datasets, sample counts, and main findings for tests of differences in total, fine, and coarse suspended sediment (SS) concentrations related to point- versus depth-integrated sampling. Note, some sites have 1–4 fewer samples for fine or coarse SS. Significant differences are those where *p* value < 0.05. Flow-adjusted concentrations are the difference between the observations and a LOESS smooth line through the concentration versus streamflow relation

	**Site**	**Analyses**	**Dataset**	**Total SS sample count**	**Findings**
Concurrent sampling methods (PI and DI)	AT-WAXL	Multiple linear regression	Samples from 9-year period with concurrent PI and DI	231 samples	Total, fine, coarse: no significant difference
		Wilcoxon signed rank test	Paired samples from 9-year period	18 paired samples	Total, fine, coarse: no significant difference
		Differences between annual estimates	WRTDS estimates of annual and FN concentrations	9 paired WY estimates	Total, fine, coarse: no significant difference FN total: PI higher, median diff 4 mg/L FN fine: no significant difference FN coarse: DI higher, median diff 1.3 mg/L
	AT-MORG	Multiple linear regression	Samples from 9-year period with concurrent PI and DI	240 samples	Total: PI 16% higher than DI Fines: No significant difference Coarse: PI 34% higher than DI Larger differences at higher flows and concs
		Wilcoxon signed rank test	Paired samples from 9-year period	20 paired samples	Total: PI higher, median diff 15 mg/L Coarse: PI higher, median diff 2.5 mg/L
		Differences between annual estimates	WRTDS estimates of annual and FN concentrations	9 paired WY estimates	Total: PI higher, median diff 15 mg/L Fines: PI higher, median diff 6 mg/L Coarse: PI higher, median diff 6 mg/L FN total: PI higher, median diff 19 mg/L FN fines: PI higher, median diff 9 mg/L FN coarse: PI higher, median diff 8 mg/L
	MS-BELL	Wilcoxon rank sum test	PI and DI samples collected in the winter and spring of 2015	12 samples per group (unpaired)	No significant differences for concentrations or flow-adjusted concentrations
Sequential sampling methods (PI then DI)	MS-UNIO	Multiple linear regression	Samples from WY2017 through WY2021 at MS-UNIO and MS-TARB. 2-years of PI samples followed by a 1-year gap and 2-years of DI samples	50 samples	Total: PI 41% higher than DI Fines: PI 30% higher than DI Coarse: PI 12 mg/L higher than DI Largest differences for total and fine SS at moderate flows and highest concs. Coarse SS consistent difference across range of flows and concs Time and sampling method are confounded
	MS-TARB			76 samples	
	OR-OUT	Multiple linear regression	Samples from WY2016 through WY2021. 3-years of PI samples followed by 3-years of DI samples	102 samples	Total, fine: no significant differences Time and sampling method are confounded
		Wilcoxon rank sum test	Coarse SS samples for above time period	102 samples	Coarse: no significant differences for concentrations or flow-adjusted concentrations Time and sampling method are confounded
	AT-SIMM	Multiple linear regression	Samples from WY2013 through WY2021. 3-years of PI samples followed by 3-year gap and 3-years of DI samples	104 samples	Total: PI 22% higher than DI Fines: PI 17% higher than DI Coarse: no significant difference Largest differences for total and fine SS at moderate flows and highest concs Time and sampling method are confounded

*PI* point-integrated samples, *DI* depth-integrated samples, *AT-WAXL* Wax Lake Outlet at Calumet, LA, *AT-MORG* Lower Atchafalaya River at Morgan City, LA, *MS-BELL* Mississippi River at Belle Chasse, LA, *MS-UNIO* Mississippi River at Union Point (mile 326), LA, *MS-TARB* Mississippi River at Tarbert Landing, MS, *OR-OUT* Old River outflow channel below hydropower channel, *AT-SIMM* Atchafalaya River at Simmesport, LA, *WY* water year, a 12-month period beginning October 1st and named using the calendar year in which it ends, *WRTDS* weighted regressions on time, discharge, and season model, *FN* flow normalized annual estimates that remove the influence of streamflow variability on annual estimates of concentration, *diff* difference, *concs* concentration, *mg/L* milligrams per liter

**Fig. 2:**
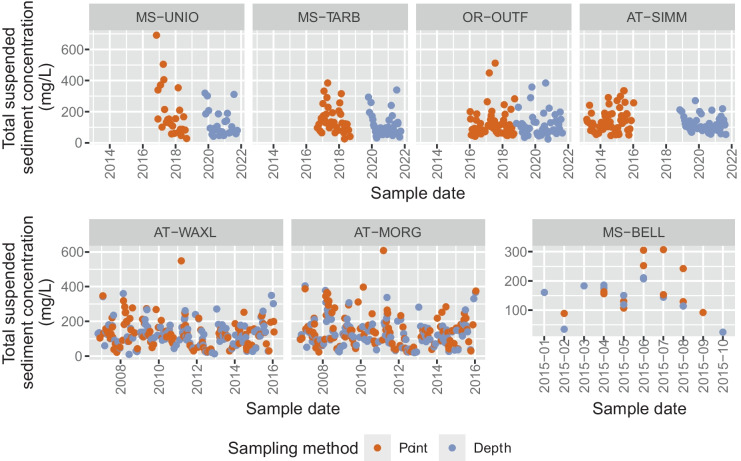
Total suspended sediment concentration data from point- and depth-integrated samples used in this study. Note, two samples are not plotted: one sample collected on 2021-08-04 at OR-OUTF with a concentration of 1540 mg/L and another sample on 2016-01-28 at AT-MORG with a concentration of 996 mg/L. MS-UNIO, Mississippi River at Union Point, LA; MS-TARB, Mississippi River at Tarbert Landing, LA; OR-OUTF, Old River outflow channel below hydropower channel; AT-SIMM, Atchafalaya River at Simmesport, LA; AT-WAXL, Wax Lake Outlet at Calumet, LA; AT-MORG, Lower Atchafalaya River at Morgan City, LA; MS-BELL, Mississippi River at Belle Chasse, LA

The four sites located around the diversion of the Lower Mississippi River (MS-UNIO, MS-TARB, OR-OUTF, and AT-SIMM, Fig. 1) were historically sampled using point-integration, and in WY 2019, the USGS began sampling using depth-integration (Fig. 2). A few samples in WY 2021 did not have a reported sampling method; we assumed these were collected using depth-integration methods. Three of the four sites have 1- to 3-year data gaps during the switch in sampling methods when no samples were collected. Depth-integrated samples were only collected in the last 2–3 years of the dataset, and point-integrated samples were often collected for several decades prior (Table 1). To create a balanced dataset with equal numbers of samples in each sampling-method group, we subsampled the point-integrated samples. First, we determined the length of time that depth-integrated samples were collected at each site and extracted the point-integrated samples for this same length of time, extending back in time from the most recent point-integrated sample (usually in WY 2019). Next, we retained these data if the numbers of point- and depth-integrated samples were the same. If there were fewer point-integrated samples than depth-integrated samples, we continued to extend back in time until there were an equal number of samples. If there were more point-integrated samples than depth-integrated samples, we randomly subsampled the point-integrated samples to the same sample count as the depth-integrated samples (Fig. 2). We refer to these datasets as having “sequential sampling methods” (Table 2).

Depth- and point-integration were used concurrently at three sites (Fig. 1) for some length of time. Two of these sites are located on the Lower Atchafalaya River (AT-WAXL and AT-MORG). We extracted a 9-year period with concurrent depth- and point-integrated sampling (Fig. 2) from the longer multidecadal record at these two sites. A subset of the samples during this period are paired samples, defined as having both a depth-integrated sample and point-integrated sample collected on the same day. These paired samples were also extracted to create paired-sample datasets for further analyses (Table 2). The most downstream site on the Mississippi River, MS-BELL (Fig. 1), also had concurrent depth- and point-integrated samples. Concurrent sampling at this site occurred for a short time in the winter and spring of 2015, and only two of the 24 samples were collected on the same day (Fig. 2). These data were also extracted from the longer record at this site and used to test for differences related to sampling methods. We refer to the datasets extracted from these three sites as having “concurrent sampling methods” (Table 2).

### Multiple linear regression

Six of the seven sites have at least 100 samples either individually or when combined with another site, and for these six sites, we used ordinary least squares MLR to test if the sampling method explained a significant (alpha = 0.05) amount of variation in SS concentration, after accounting for as much seasonal, flow-related, between-site, and temporal variability as possible. For most MLR models, the response variable was the natural logarithm of concentration, but because coarse SS sediment data contains values of zero, we added “1” to all these concentrations prior to log-transforming. Five negative flow values in the datasets were used for developing the MLR models, two at Wax Lake Outlet (AT-WAXL) and three at Morgan City (AT-MORG). For these values, we substituted the minimum, non-negative flow value at each site. We modeled the Mid-Lower Mississippi River sites (MS-UNIO and MS-TARB) together because they had the same period of record (Fig. 2) and lower individual sample counts. We developed two MLR models for each combination of site(s) and sediment size fraction: a covariate-only model and a covariate-plus-sampling-method model.

While developing the covariate-only models, we tried to explain as much variability in SS concentration as possible without including a term for sampling method. Potential explanatory variables included daily streamflow with polynomial terms, flow anomaly terms (computed using the waterData R package by Ryberg & Vecchia, 2017), hysteresis terms (Garrett, 2012), seasonal terms using 2 and 4 pi cycles, a categorical variable for site if the dataset contained more than one site, a categorical variable for flows below or above 100,000 cubic feet per second (cfs) at the time of sample collection, and a time trend term. We rescaled decimal date for each dataset, so that January 1st of the calendar year containing the first sample equals zero. We used best subsets within the leaps R package (Lumley, 2020) to compute Bayesian Information Criterion (BIC), adjusted coefficient of determination (*R*^2^), and Mallow’s Cp statistics for the top three models (based on the models’ residual sum of squares) for each k-parameter model. We reviewed plots of the statistics versus k parameters and a list of the variables contained with the top 3 models to select a subset of models for further evaluation using residual plots. During review of the residual plots, we checked for model assumptions (independent and identically distributed (i.i.d.) errors) and appropriate model fit. We evaluated the normality of the residuals using quantile–quantile (Q-Q) plots, histograms, and the Shapiro–Wilk normality test available in the stats R package (R Core Team, 2022). We also explored the use of additional explanatory variables and interaction terms during this process. We only used a more complex model if supported by the results of an F-test that compared the simpler model to the more complex model. In addition to reviewing residual plots and checking for model assumptions, we also reviewed variance inflation factors (VIFs), Akaike Information Criterion (AIC), adjusted *R*^2^, and predicted residual error sum of squares (PRESS) statistics to ultimately select the final covariate-only model for each combination of site(s) and sediment fraction.

Once the covariate-only model was satisfactory, we created a second MLR model by adding a two-level categorical variable for sampling method to the covariate-only model. If the coefficient of the sampling method variable was significant (alpha = 0.05), this provided evidence that sampling method explained a meaningful portion of the concentration variability after controlling for covariates. Model assumptions were reviewed again at this point. Because the response variable is log-transformed, the coefficient for sampling method can be converted to a percentage using this equation: 100*(exp(coefficient) − 1). Time was excluded as an explanatory variable for sites that had sequential sampling methods (MS-UNIO, MS-TARB, OR-OUTF, and AT-SIMM) because the point- and depth-integrated sampling occurred during distinct periods of time (Fig. 2).

Finally, we used the emmeans R package (Lenth, 2023) to estimate marginal means and 95% confidence intervals of total, fine, and coarse SS for each sampling method and site. Marginal means, also referred to as least-squares means or predicted means, are not the observed means computed from the data; instead, they are estimates of the means from the statistical model. The benefit of computing and comparing marginal means is that they represent the average response of concentration at particular levels of a categorical variable, after controlling for other covariates (usually using the mean value if the covariate is continuous). In addition to estimating marginal mean concentrations for the point- and depth-integrated samples, we also tested for significant differences (i.e., contrasts) between these marginal means. The estimated marginal means, contrasts, confidence intervals, and tests of difference were initially computed in units of the response variable (i.e., log(concentration), log(concentration + 1), or concentration) and then retransformed to units of concentration, if appropriate, using the same R package.

### Nonparametric tests

For small datasets (i.e., less than 50 samples) or those that could not be modeled appropriately using MLR (i.e., errors not i.i.d.), nonparametric techniques were used to test for differences between depth- and point-integrated samples. The Wilcoxon signed rank test was used with the paired sample datasets. Paired sample datasets were those when a point-integrated sample and depth-integrated sample were collected on the same day at the same site. The length of time between paired samples was typically 1–2 h with the longest gap being 7 h. The null hypothesis for the Wilcoxon signed rank test is that the median difference between all paired samples is zero. If samples were not paired, the Wilcoxon rank sum test was used. The null hypothesis for this test is that the samples were drawn from the same population. This is often interpreted as a test for differences in group medians; however, that is only true if the populations have the same shape. All tests were run separately for each combination of site and sediment fraction, using concentrations in milligrams per liter (mg/L). The Wilcoxon rank sum test was also used on flow-adjusted concentrations from the unpaired datasets. Flow-adjusted concentrations were computed by fitting a LOESS line through the concentration by streamflow relation for each sampling method and subtracting the observed concentrations from this line. An alpha level of 0.05 was used to determine the significance of all the tests, and the effect size (either the difference in medians or median difference) was reported. The base R wilcox.test function was used for the Wilcoxon tests (R Core Team, 2022).

### Comparison of annual estimates

For the two Lower Atchafalaya River sites (AT-WAXL and AT-MORG) with 9-year records of concurrent sampling methods, we estimated annual mean concentrations for total, fine, and coarse SS and investigated the possible effect of sampling method on these estimates. We used the weighted regressions on time, discharge, and season with Kalman filter model (WRTDS-K) (Hirsch et al., 2010; Zhang & Hirsch, 2019) to estimate annual and flow-normalized concentrations. WRTDS-K is implemented through the R package Exploration and Graphics for RivEr Trends (EGRET) (Hirsch & De Cicco, 2015). The following multiple regression equation is the basis of WRTDS,

$$\mathrm{ln}\left(c\right)={\beta }_{0}+{\beta}_{1}T+{\beta}_{2}Q+{\beta }_{3}\;\text{sin}\left(2\pi T\right)+{\beta }_{4}\;\text{cos}\left(2\pi T\right)+\varepsilon$$

where *c* is concentration in milligrams per liter, *β*_*0-4*_ are the regression coefficients, *Q* is natural log of daily mean streamflow, *T* is decimal time, and *ε* is the error (unexplained variation).

During calibration, WRTDS uses moving windows and weights the water-quality measurements in relation to time, streamflow, and season to estimate daily concentrations, which are aggregated to seasonal or annual mean concentrations and loads. The flexible weighting that WRTDS uses permits the coefficients to vary through time, allowing the model to respond to evolving, and often non-monotonic, changes in the system (Hirsch et al., 2010). WRTDS-K is identical to WRTDS but includes a Kalman filter that adjusts the daily estimates based on the serial correlation of the observed residuals (Zhang & Hirsch, 2019).

Annual mean concentrations can be strongly influenced by flow conditions, which can have high variability as a result of variable weather. To control for and remove this flow-related variability, we used the “flow-normalized” (FN) estimates from WRTDS. WRTDS uses integrated probability distributions of daily streamflow and the model coefficients for each day to estimate these FN concentrations over time (Hirsch et al., 2010). FN estimates are often used for characterizing non-monotonic changes in water quality over time (i.e., trends), but here, we use them to control for flow-related variability.

Measures of uncertainty for the WRTDS output were estimated using a block bootstrap, implemented through the R package EGRETci (Hirsch & De Cicco, 2015). This extension of WRTDS uses a sampling block that is based on a time interval (instead of a sample count) to adequately bootstrap from periods with differing data densities. Prediction intervals for the annual mean estimates and confidence intervals for the FN estimates were computed using this approach.

We calibrated two WRTDS-K models for each SS fraction (total, fine, and coarse) at the two lower Atchafalaya River sites (AT-WAXL and AT-MORG). One model was calibrated using only point-integrated samples and another only using depth-integrated samples. This resulted in 12 WRTDS-K models that were used to estimate annual and FN concentrations. The WRTDS-K models and bootstrap analyses were computed with default specifications. These were windowY = 7, window = 2, windowS = 0.5, minNumObs = 100, minNumUncen = 50, and edgeAdjust = TRUE. The Kalman filter options were rho = 0.9 and niter = 200. The daily estimates were aggregated by WY from WY 2007 through WY 2015. We also computed 90% prediction and confidence intervals for the annual and FN estimates, respectively. Default specifications for generating the annual prediction intervals were nBoot = 10, nKalman = 10, and rho = 0.9, and for the FN confidence intervals were nBoot = 100 and blockLength = 200. Each fitted model was reviewed using residual plots, and error statistics were computed using the EGRET package.

For the annual and FN values, we compared the estimates from the point-integrated and depth-integrated model calibrations to assess effects of differences in sampling method. We considered estimates from the two calibrations for the same WY to be paired samples and used the nonparametric Wilcoxon rank sum test to identify statistically significant differences and effect sizes by sampling method for each site and sediment fraction. Additionally, we also counted the number of WYs where the depth- and point-integrated uncertainty intervals did not overlap.

## Results

### Summary of field methods over time

**Fig. 3:**
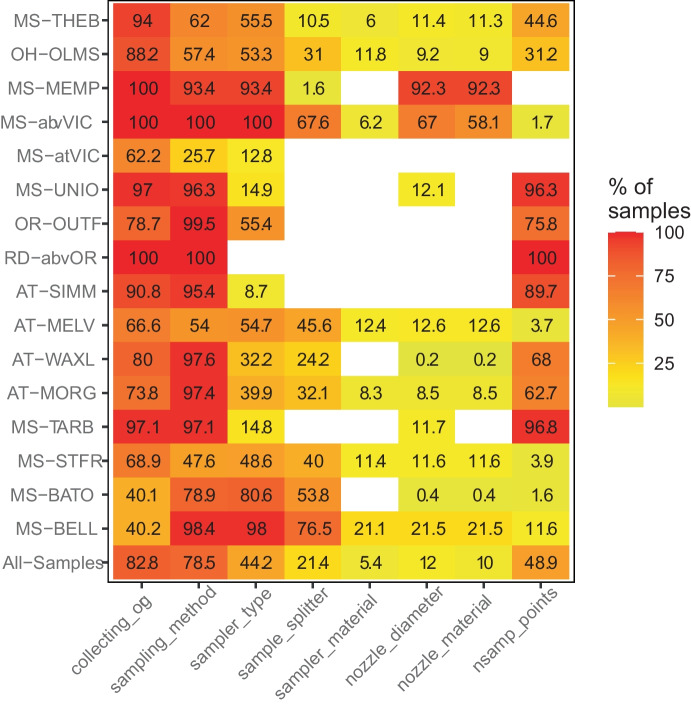
Percentage of samples at each site that report a given type of field method information. All-Samples is the percentage of all samples across all sites in the study. A white box indicates that no samples at that site reported this information. Refer to Table 1 for full site names

Of the eight USGS field method pcodes considered in this study, only two, collecting organization and sampling method, were regularly reported (Fig. 3). Across the 16 sites, collecting organization was almost entirely “USGS” or “USGS/USACE” with a few samples at the Ohio River site (OH-OLMS) collected by the Illinois Environmental Protection Agency (Online Resource 6). The availability of sampling method information varied by site from 100% of the samples (MS-MEMP and RD-abvOR) to about 26% of the samples (MS-atVIC; Fig. 3). About one-third of all the samples report sampling method as “composite-points” (i.e., point-integrated techniques) and another 40% reported either “EDI” or “EWI” (i.e., depth integrated techniques; Online Resource 6). Samples collected using point-integration were exclusively from Atchafalaya River sites (MS-UNIO, AT-SIMM, AT-WAXL, AT-MORG) and three sites around the Old River Control Structure (OR-OUTF, RD-abvOR, and MS-TARB). These sites were historically sampled by the USACE. Depth-integrated techniques were more common at Mississippi River sites. EDI sampling tended to be used at sites farther upstream (MS-THEB, OH-OLMS, MS-MEMP and MS-abvVC), and EWI sampling was common at downstream sites, especially those in the lowermost Mississippi River (Online Resource 6).

Sampler type and the number of sampling points were reported for about half the samples, while the remaining four pieces of sampling information (sample splitter, sampler material, nozzle diameter, and nozzle material) were reported for about 5% and 21% of the samples, if reported at all (Fig. 3). Sample splitter may not be reported for some samples because splitters are not always used when collecting suspended sediment. Field samplers US D-96 and US D-99 were commonly used, with fewer sites reporting use of a point sampler (Online Resource 6). Although this study focuses on comparing point- and depth-integrated sampling methods, other field method comparisons can be made using this database. For example, differences in nozzle diameters, number of sampling points, sampler or nozzle materials, and between EWI and EDI sampling methods could be evaluated. Some of these comparisons have already been made in a laboratory but this database provides an opportunity to evaluate differences across multiple sites and flow conditions in a field setting. The Online Resources 4 and 5 provide timeline plots of NWIS-derived field method information by site and by field method information, respectively. Online Resource 6 shows the frequency of different types of field method information. Finally, Online Resource 2 summarizes much of this material and supplements it with information from field staff and other resources to provide a compiled timeline of field method activities for each site.

### Multiple linear regression results

All covariate-only models were statistically significant although the amount of variability explained by each model varied by dataset and sediment fraction (Table 3). The adjusted *R*^2^ tended to be high (0.66–0.92) for models that were calibrated with the 9-year datasets (AT-WAXL and AT-MORG). *R*^2^ was also high (0.84) for the coarse SS model for the Atchafalaya River at Simmesport (AT-SIMM). Much less variability was explained by the other covariate-only models, which were all fit using datasets that had sequential sampling methods. Although still statistically significant, *R*^2^ values were between 0.22 and 0.39 (Table 3).

**Table 3:** Multiple linear regression model results for the covariates-only model [cov] and the covariates + sampling method model [cov + samp], by site/dataset and sediment fraction. Refer to Online Resource 1, Table SI1-1 for fitted equations and Table SI1-2 for list of outliers removed

	**Sites used in model**	**Sediment fraction**	**Response variable**	**Model**	**DOF**	**Adjusted R**^**2**^	**AIC**	**Sampling method** ***p*** **value (bold if < 0.05)**	**Sampling method coefficient (difference)**
Concurrent sampling methods	AT-WAXL	Total	ln(c)	cov	223	0.79	109	–	–
				cov + samp	222	0.79	109	0.126	0.06 (6%)
		Fine	ln(c)	cov	221	0.73	130	–	–
				cov + samp	220	0.73	130	0.126	0.07 (7%)
		Coarse	ln(c + 1)	cov	219	0.92	220	–	–
				cov + samp	218	0.92	220	0.227	-0.06 (-6%)
	AT-MORG	Total	ln(c)	cov	231	0.78	130	–	–
				cov + samp	230	0.79	118	**< 0.001**	**0.15 (16%)**
		Fine	ln(c)	cov	230	0.66	180	–	–
				cov + samp	229	0.66	179	0.084	0.08 (8%)
		Coarse	ln(c + 1)	cov	229	0.86	452	–	–
				cov + samp	228	0.86	441	**0.003**	**0.29 (34%)**
Sequential sampling methods	MS-UNIO & MS-TARB (Mid-lower Mississippi)	Total	ln(c)	cov	121	0.22	223	–	–
				cov + samp	120	0.28	214	**< 0.001**	**0.34 (41%)**
		Fine	ln(c)	cov	121	0.32	194	–	–
				cov + samp	120	0.36	188	**0.005**	**0.26 (30%)**
		Coarse	c	cov	117	0.39	1127	–	–
				cov + samp	116	0.41	1125	**0.044**	**12 (mg/L)**
	OR-OUTF	Total	ln(c)	cov	96	0.24	159	–	–
				cov + samp	95	0.24	161	0.870	0.02 (2%)
		Fine	ln(c)	cov	96	0.28	142	–	–
				cov + samp	95	0.27	144	0.572	0.06 (6%)
	AT-SIMM	Total	ln(c)	cov	100	0.38	91	–	–
				cov + samp	99	0.41	86	**0.010**	**0.20 (22%)**
		Fine	ln(c)	cov	99	0.37	97	–	–
				cov + samp	98	0.39	95	**0.046**	**0.16 (17%)**
		Coarse	ln(c + 1)	cov	97	0.84	149	–	–
				cov + samp	96	0.84	150	0.691	0.04 (4%)

Bold entries are statistically significant (p < 0.05)

*DOF* degrees of freedom, *AIC* Akaike Information Criterion, *In(c)* natural log of concentration, *c* concentration in milligrams per liter (mg/L), *---* not applicable, *AT-WAXL* Wax Lake Outlet at Calumet, LA, *AT-MORG* Lower Atchafalaya River at Morgan City, LA, *MS-UNIO* Mississippi River at Union Point, LA, *MS-TARB* Mississippi River at Tarbert Landing, LA, *OR-OUTF* Old River outflow channel below hydropower channel, *AT-SIMM* Atchafalaya River at Simmesport, LA

The form of the covariate-only models varied by site and sediment parameter (Online Resource 1, Table S1-1 provides fitted equations). Some noteworthy variations in model form include the use of untransformed concentration as the response variable in the coarse SS model for the mid-lower Mississippi River sites (MS-UNIO and MS-TARB), which changes the interpretation of the coefficient for sampling method. Also, although nonsignificant, we retained the 2-level categorical site variable for the mid-lower Mississippi models to illustrate differences, or lack thereof, between these sites. Finally, no coarse SS model was fit for the Old River outflow channel site (OR-OUTF) because the five highest coarse SS concentrations could not be adequately modeled. These uncharacteristic concentrations could be the result of samples collected during or near a flushing event through the Old River Control Structure and thus not representative of normal operating conditions.

Across all the datasets used for MLR, 11 outliers were identified and removed during the development of the covariate-only models (Online Resource 1, Table SI1-2). Removing these outliers allowed the residuals to be normally distributed, a key modeling assumption for MLR. After removing outliers, only one model, the covariate-only model for coarse SS at mid-lower Mississippi (MS-UNIO and MS-TARB), had non-normal residuals. Residuals from the models that included sampling method (cov + samp) were normally distributed. We also re-ran all the models with the outliers in the calibration datasets and found the significance of the sampling method coefficients (whether *p* value > or < 0.05) did not change; thus, the outliers have no effect on our interpretation of the MLR results.

When sampling method was added to the covariate-only models, this coefficient was statistically significant in 7 of the 14 models (Table 3). This included models for all SS fractions at the mid-lower Mississippi River sites (MS-UNIO and MS-TARB), total and fine SS at Simmesport (AT-SIMM), and total and coarse SS at Morgan City (AT-MORG). For these seven models, the AIC decreased and the *R*^2^ typically increased (+ 0.03 on average) compared to their corresponding covariate-only model (Table 3), indicating that differentiation by sampling method explained a meaningful amount of variability in concentration even after controlling for season, time, various aspects of flow, and between-site differences. Additionally, the coefficients were all positive, indicating samples collected using point integration tended to have higher concentrations than those collected using depth integration. The bias between point- and depth-integrated sampling is multiplicative for most of the sites as demonstrated by the need for a log-transformed response variable in the MLR. Only one model, coarse SS for the mid-lower Mississippi River sites (MS-UNIO and MS-TARB), indicated an additive bias of sampling method based on the untransformed response variable. As such, we can expect the bias to be larger at higher concentrations at most sites.

**Fig. 4:**
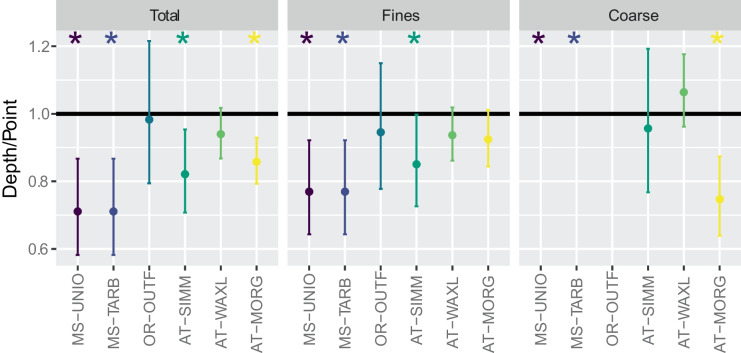
Ratio and 95% confidence interval of estimated marginal means for point- and depth-integrated samples from multiple linear regression (MLR) models. Because models are in log-space, differences in marginal means are expressed as a ratio (Depth/Point) when retransformed. Black line is at *y* = 1, indicating no difference in concentrations based on sampling method. Stars indicate statistically significant (*p* < 0.05) differences between point- and depth-integrated samples, computed using units of the response variable. Recall MS-UNIO and MS-TARB are included in the same MLR model. No ratio or confidence intervals are shown for MS-UNIO and MS-TARB because the response variable for this model was in untransformed units of concentration. Contrasts for MS-UNIO and MS-TARB are 19.6 mg/L (point- minus depth-integrated marginal means). There was no appropriate MLR model for OR-OUTF coarse SS. AT-WAXL, Wax Lake Outlet at Calumet, LA; AT-MORG, Lower Atchafalaya River at Morgan City, LA; MS-UNIO, Mississippi River at Union Point, LA; MS-TARB, Mississippi River at Tarbert Landing, LA; OR-OUTF, Old River outflow channel below hydropower channel; AT-SIMM, Atchafalaya River at Simmesport, LA

Because most of the models were fit in log space, the ratio of the marginal means shows the relative difference between sampling methods (Fig. 4). A ratio of 1 indicates no difference. Results show significantly low ratios (depth/point) for total SS for all sites except the Old River outflow channel (OR-OUTF) and Wax Lake Outlet (AT-WAXL). The lowest ratios occur for the Mid-Lower Mississippi River sites (MS-UNIO and MS-TARB). A similar pattern is apparent for fine SS, except Morgan City (AT-MORG) has a nonsignificant difference. Fine SS ratios are smaller compared to total SS ratios for some sites but not others. Although total and fine SS show a similar pattern of magnitudes across the sites (i.e., MS-UNIO and MS-TARB have the lowest ratios and OR-OUTF the highest), this pattern diverges for coarse SS (Fig. 4). This is partly because the mid-lower Mississippi River sites (MS-UNIO and MS-TARB) have concentration differences in mg/L, not ratios, and thus are not plotted on Fig. 4, but also because the Morgan City site (AT-MORG) has a much lower ratio for coarse SS compared to the corresponding ratios for total and fine SS, indicating large relative differences between sampling methods for coarse SS at this site (Fig. 4).

**Fig. 5:**
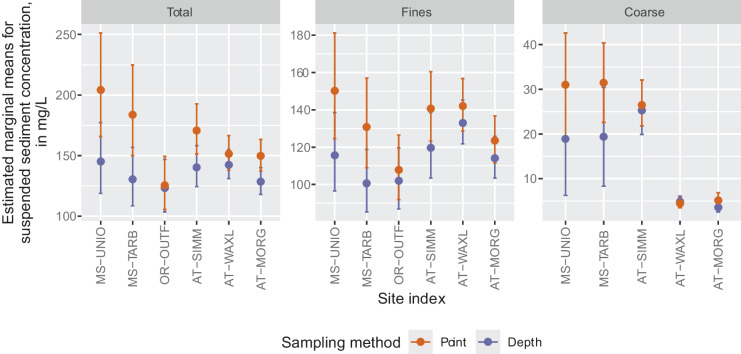
Estimated marginal means (solid points) and 95% confidence intervals (vertical lines) of suspended sediment (SS) concentration after controlling for covariates. Retransformed estimates have been bias corrected using the residual standard error of each model, when appropriate. Recall, sites MS-UNIO and MS-TARB are in the same multiple linear regression (MLR) model. AT-WAXL, Wax Lake Outlet at Calumet, LA; AT-MORG, Lower Atchafalaya River at Morgan City, LA; MS-UNIO, Mississippi River at Union Point, LA; MS-TARB, Mississippi River at Tarbert Landing, LA; OR-OUTF, Old River outflow channel below hydropower channel; AT-SIMM, Atchafalaya River at Simmesport, LA

Additionally, we used the marginal means and confidence intervals computed from the MLR models to interpret how mean concentrations of SS, adjusted for covariates, vary between sites, sampling methods (Fig. 5), and by streamflow (Online Resource 1, Fig. SI1-2). Regardless of sampling method, coarse SS marginal mean concentrations are considerably lower than total and fine SS, and especially so for the two Atchafalaya River sites (AT-WAXL and AT-MORG; note different y-axis on Fig. 5). Marginal means for point-integrated samples were higher, although not always significantly higher, than depth-integrated samples for all sites and sediment fractions, except for coarse SS at the Wax Lake Outlet (AT-WAXL; Fig. 5). Marginal means also varied with streamflow magnitude when plotted against the 10th through 90th percentiles of streamflow (Online Resource 1, Fig. SI1-2). All sites had the highest coarse SS concentration at or above the 70th percentile of flow. Similarly, the highest total and fine SS concentrations at the two lower Atchafalaya sites (AT-WAXL and AT-MORG) were also at or above the 70th percentile of flow. However, the other sites had the highest total and fine SS concentrations at moderate flows (40 − 50th percentiles). These patterns were similar for both point- and depth-integrated samples. Additionally, the flow percentiles with the highest concentrations also align with the largest bias in concentration between point- and depth-integrated samples (Online Resource 1, Fig. SI1-3).

### Nonparametric test results

The results of the Wilcoxon tests indicated few significant differences between concentrations collected using different sampling methods (Online Resource 1, Table SI1-3) and corroborate the results of the MLR models and estimated marginal means (Table 2). Wilcoxon signed rank tests of paired samples at Morgan City (AT-MORG) indicate point-integrated samples were 15 mg/L and 2.5 mg/L higher than depth-integrated samples for total and coarse SS, respectively. Given mean concentrations of 147 and 27 mg/L for total and coarse SS at this site, these differences equate to about a 10% relative bias. Fine SS at this site was 7.5 mg/L higher for point-integrated samples, although nonsignificant (*p* value = 0.055). No significant differences were found for total, fine, or coarse SS at the other lower Atchafalaya River site (AT-WAXL). Similarly, no significant differences for concentrations or flow-adjusted concentrations were found for any of the sediment fractions at Belle Chasse (MS-BELL). Finally, because an adequate model using MLR could not be developed for coarse SS at the Old River Outflow Channel (OR-OUTF), Wilcoxon rank sum tests were used to test for differences in concentration and flow-adjusted concentration for coarse SS at this site. Even with more than 100 samples, no significant differences were found (Table 2).

### Comparison of annual estimates results

**Fig. 6:**
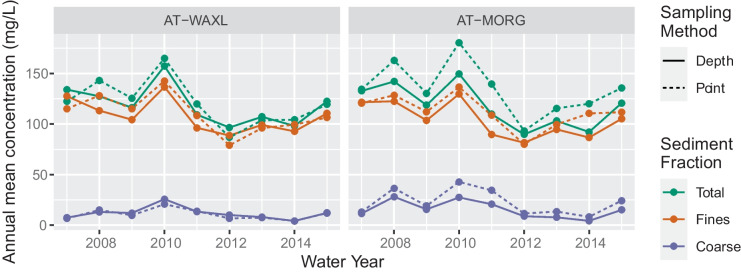
Annual estimates of total, fine, and coarse suspended sediment (SS) for the two Lower Atchafalaya River sites, Wax Lake Outlet (AT-WAXL), and Morgan City (AT-MORG). Estimates computed from weighted regressions on time, discharge, and season (WRTDS) models calibrated separately using only depth- or point-integrated samples

The 12 WRTDS models for the two lower Atchafalaya River sites, each calibrated with only depth- or point-integrated samples (Fig. 6), had relatively high *R*^2^ values, ranging from 0.42 to 0.91, with higher *R*^2^ values for the coarse SS (Online Resource 1, Table SI1-4). Flux bias statistics (a measure of modeled bias based on days with observed samples) for fine and total SS models were low, ranging between 0.007 and − 0.021, respectively, indicating a bias near 0%. However, the coarse SS models had much higher flux bias statistics ranging from 0.125 to 0.455 across the four models, indicating a tendency for the models to overestimate coarse SS loads. Across all the WRTDS models, root mean square error (RMSE) in log-concentration ranged from 0.347 to 0.900; when retransformed (exponentiated), this equates to between 1.4 and 2.5 mg/L. The highest RMSEs were for the coarse sediment models (Online Resource 1, Table SI1-4).

**Fig. 7:**
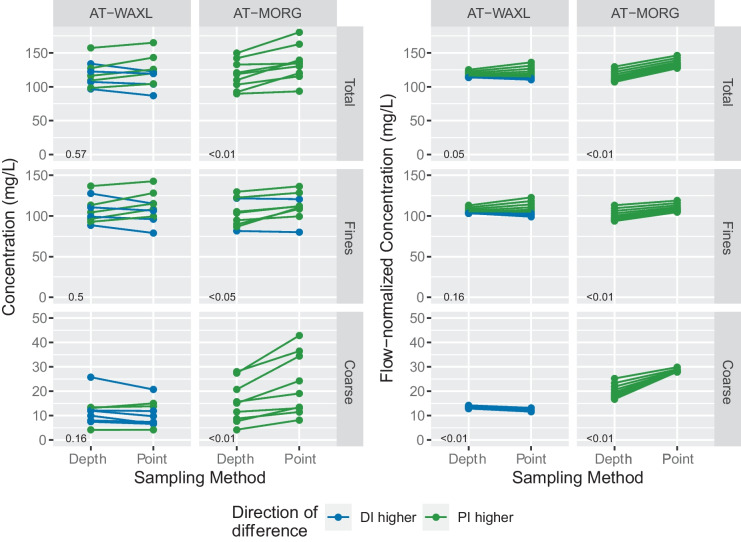
Annual and flow-normalized estimates of total, fine, and coarse suspended sediment (SS) for the two Lower Atchafalaya River sites, Morgan City (AT-MORG), and Wax Lake Outlet (AT-WAXL). Lines connect estimates from the same water year. *p* values in lower left are from Wilcoxon rank sum tests

The annual estimates from the point-integrated samples were routinely higher than the depth-integrated samples at Morgan City (AT-MORG), and WYs with higher concentrations also tended to have larger differences. However, for Wax Lake Outlet (AT-WAXL), the pattern is less consistent (Fig. 6). Differences of depth- minus point-integrated estimates for the same WY range from − 31 to 1.5 mg/L (mean − 11 mg/L) at Morgan City (AT-MORG) and from − 16 to 12 mg/L (mean − 1.2 mg/L) at Wax Lake Outlet (AT-WAXL), depending on the sediment type. Differences were statistically significant for total, fine, and coarse SS at Morgan City (AT-MORG) and nonsignificant at the Wax Lake Outlet (AT-WAXL) (Fig. 7). Differences in the FN estimates, which removes much of the flow-related variability from the annual estimates, are also statistically significant at Morgan City (AT-MORG). The mean annual percentage difference between sampling methods is 16%, 8.5%, and 43% for total, fine, and coarse SS, respectively. Although all the significant differences indicate higher annual concentrations from models calibrated with point-integrated samples compared to depth-integrated samples, unexpectedly, FN coarse SS at Wax Lake Outlet (AT-WAXL) shows the opposite (Fig. 7), a pattern we cannot explain.

We compared the 90% confidence and prediction intervals of the annual and FN estimates, respectively, for the paired WYs and found that the intervals almost always overlap between the calibrations. The only exceptions across the 36 comparisons (i.e., 9-year period, two estimate types (annual and FN), and two sites) were 2, 1, and 1 WYs for total, fine, or coarse annual mean SS, respectively, at the Atchafalaya River at Morgan City (AT-MORG). The intervals overlapped for all WYs at Wax Lake Outlet (AT-WAXL), indicating an inability to distinguish differences in estimates based on sampling methods.

**Fig. 8:**
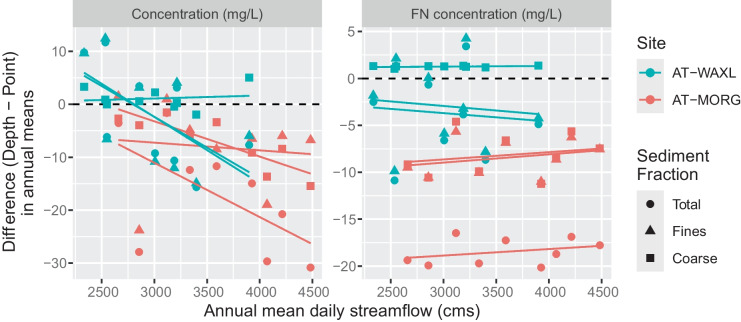
Differences of annual and flow-normalized estimates between the depth- and point-integrated calibrations plotted against annual mean daily streamflow. mg/L, milligrams per liter; cms, cubic meters per second; AT-WAXL, Wax Lake Outlet at Calumet, LA; AT-MORG, Lower Atchafalaya River at Morgan City, LA

Lastly, similar to the marginal means from the MLR models, the annual estimates from WRTDS also indicate an effect of streamflow magnitude on the concentration bias. WYs with higher flows also had larger differences in the annual estimates between sampling methods (Fig. 8). The effect is particularly strong at Morgan City (AT-MORG), although somewhat apparent at Wax Lake Outlet (AT-WAXL). Once we remove the flow-related variability using FN, the bias is nearly constant across the range of flows. For Morgan City (AT-MORG), approximate differences between sampling methods (depth-point), after removing flow effects, are − 19, − 9, and − 8 mg/L for total, fine, and coarse SS, respectively. For the Atchafalaya River at Wax Lake Outlet (AT-WAXL), approximate differences are smaller, − 4, − 3, and 1 mg/L for total, fine, and coarse SS, respectively.

## Discussion

Across the seven sites and various analyses used in this study, SS samples collected using point-integrated methods tended to have higher concentrations than those collected using depth-integrated methods. However, the absolute and relative bias was variable (Table 2). The MLR models, annual estimates from WRTDS, and nonparametric analyses all indicate the presence and magnitude of the bias is inconsistent across sites. The log-transformed response variable used with most of the MLR models indicates a multiplicative effect where higher concentrations have larger bias. Additionally, these models and the WRTDS analyses indicate larger bias at moderate and high flows (Table 2).

Across the three analyses, some measures of effect size indicate relatively small though statistically significant differences between sampling methods, whereas other measures indicate larger, meaningful effect sizes. The addition of the sampling method coefficient to the MLR equations indicates only a small increase in the variability explained. The change in adjusted *R*^2^ (Table 3) ranged from 0.00 to 0.06 with a mean change 0.03 (i.e., 3% more variability in log-concentration explained). Similarly, the Wilcoxon tests indicate effects sizes of 15 mg/L and 2.5 mg/L for total and coarse SS at Morgan City (AT-MORG), respectively, or relative differences of approximately 10%, arguably a small difference for SS datasets that are inherently noisy and often contain considerable amounts of uncertainty. On the other hand, the magnitudes of the statistically significant sampling method coefficients from the MLR equations range from 0.15 to 0.34 with a mean of 0.23, which equate to differences between sampling methods of 16 to 41% (mean of 26%) after controlling for covariates, depending on the site (Table 3). Additionally, the flow normalized estimates at Morgan City (AT-MORG), which remove much of the effect of year-to-year variability in flow, indicate mean percentage differences of 16%, 8.5%, and 43% for total, fine, and coarse SS concentrations, respectively (Fig. 7). Taken together, this supports our conclusion that the presence and magnitude of the sampling method bias vary depending on the site and also for total, fine, or coarse SS fractions.

Although significant differences were found at three of the four sites with sequential sampling methods (MS-UNIO, MS-TARB, and AT-SIMM; Table 2), we must acknowledge these tests provide some of the weakest evidence of meaningful differences. As mentioned previously, point- and depth-integrated techniques were used during different time periods (Fig. 2) and thus represent different environmental conditions. We tried to control for this effect by including a variety of covariates in the MLR models, but the models generally explained only about 30 to 40% of the SS concentration variability at these sites. Because sampling method is confounded with time at these sites, it is difficult to know whether some or all the bias is due to different sampling methods or different environmental conditions. This confounding may also explain the considerably high concentration differences, ranging from 17 to 41%, found at these sites (Table 2). Additional years of data collected using depth-integrated techniques and a test for a step change are likely the best way to assess the effect of sampling method changes in these datasets.

The datasets available at the two Lower Atchafalaya River sites (AT-WAXL and AT-MORG) provide the best information for evaluating differences in sampling method due to both sampling techniques being used over a 9-year period. Unexpectedly, these sites show conflicting results. Wax Lake Outlet (AT-WAXL) consistently showed no significant differences between sampling methods across the analyses whereas Morgan City (AT-MORG) had significant differences for total, fine, or coarse SS, or some combination of these depending on the analysis (Table 2). The differences in FN concentrations at Morgan City (AT-MORG) likely give the best estimate for the magnitude of the bias, after removing flow effects, which appears to be about 19, 9, and 8 mg/L for total, fine, and coarse SS, respectively. Given the positive relationship between bias magnitudes, SS concentrations, and streamflow, the relative bias (%) estimated from the MLR models provide a measure of the relative difference; as such, point-integrated samples of total and coarse SS have concentrations that are 16% and 34% higher, respectively, than depth-integrated samples (Table 2).

Discussion with sampling crews (Lane Simmons, US Geological Survey, written communication, July 10, 2023) and review of sampling information (Online Resource 2) indicates additional considerations when comparing the two Lower Atchafalaya River sites. The hydrology, channel geometry, bed material, and sampling conditions differ between these sites. Wax Lake Outlet (AT-WAXL) has a classic trapezoidal shaped channel with relatively uniform depth, a hard bottom, and close to uniform flow—recommended site conditions for sampling SS (Rantz et al., 1982; Guy & Norman, 1970; Edwards & Glysson, 1999). Conditions at Morgan City (AT-MORG) are quite different. Morgan City (AT-MORG) is a wide channel where flow is largely concentrated in about 60% of the width. A large shoal with lower flows is on one side of the channel. Also, a confluence is upstream, and the channel is periodically dredged. Additionally, Morgan City (AT-MORG) tends to have higher total and coarse SS concentrations than Wax Lake Outlet (AT-WAXL), although both have lower coarse SS concentrations than the other sites in this analysis (Fig. 5).

Beyond physical and environmental differences between the two Atchafalaya River sites, some differences are likely in the implementation of the point- and depth-integrated sampling methods (Lane Simmons, US Geological Survey, written communication, July 10, 2023). Different crews from different agencies collected these samples (Online Resource 4). There may have been differences in the number and placement of the verticals between the crews. Due to the difficulties of sampling large rivers, the verticals may have been at fixed points, not necessarily at equal widths or streamflow. Furthermore, the number of points and the number of verticals varied over time (Online Resource 2). Specifically at these two sites, there was a shift around WY 2018 from using 3 to 5 verticals across the channel cross section when collecting depth-integrated samples. However, the 9-year dataset used in these analyses was collected prior to that shift (Fig. 2). This is another field method change that could be explored with the datasets in Murphy et al. (2022) and the Online Resources attached to this report. Finally, at the Morgan City site (AT-MORG), one crew may have been sampling the large shoal area while another crew did not. As such, it is difficult to tease out the confounding effects of these variations with the available information.

One possible conclusion is that some variations in the application of point- and depth-integrated sampling methods have little to no effect on the collected SS concentrations at an ideal sampling location (e.g., AT-WAXL) but do have a larger influence at less-than-ideal locations, especially if the site has higher coarse SS and more heterogenous SS concentrations across the cross section. Other studies have noted larger differences in SS concentration when different field methods are applied at less-than-ideal sites. For example, Allen and Peterson (1981) compared replicate samples collected from a single depth-integrated vertical, using the Equal Transit Rate approach, and found differences as large as 30%. These larger differences tended to happen when (1) samples were collected by less experienced staff, and (2) samples were collected at the less-than-ideal sampling location. In the case of Allen and Peterson (1981), this meant a location with a large channel, truss bridge, and high sand load. Edwards and Glysson (1999) point out that the sampling site should be located sufficiently downstream from the confluence between a tributary and mainstem so that sufficient mixing is achieved. This is particularly important for coarse SS as it tends to be less uniform across the channel compared to fine SS. Finally, additional errors may arise when too few verticals are used for non-trapezoidal shaped cross sections. Errors can be as great as + / − 40% compared to a more uniform, trapezoidal cross section where errors can be an order of magnitude less, ≤ +/−4% with at least 5 verticals (Edwards & Glysson, 1999; Guy & Norman, 1970; Topping et al., 2011). Nevertheless, for some rivers, like some of the Atchafalaya River distributaries, no site location is ideal, which makes the tracking of field method information and site characteristics over time even more important at these sites compared to ideally located sites. Perhaps in locations like these, more vertical profiles, additional vertical point samples, or both, would minimize the concentration differences between depth- and point-integrated sampling methods.

Another mechanism that may be at play is the stratification of SS in the water column and differences in how samples collected near the bottom of the channel are weighted during the computation of the SS profile of the vertical. Large river SS concentrations are expected to vary with depth, especially in the lower portions of the water column. Near the bottom of the river, bed material can become entrained and provides an in-stream supply of sediment to the water column that can be captured in the SS sample. The amount of sediment that contributes to the water column is dependent on near-bottom stream velocities, bed sediment material, and bedload conditions at the sampling location. A study in the Lower Yellow River in China showed that stratification increased with increases in SS concentration, especially in rivers with low channel bed slopes (Moodie et al., 2022). Work in the lowermost Mississippi River shows longitudinal differences in bed material also influence the proportion of fine and coarse material in the water column, resulting in higher fine SS concentrations downstream from the Old River diversion due to greater access to the fine sediment comprising the channel bottom in the lower part of the river (Mossa, 1996). Sample collectors using point-integrated methods in the Lower Mississippi and Atchafalaya Rivers typically collect between 2- and 5-point samples at various depths for each vertical (Online Resource 2). Given this lean vertical distribution of samples within a water column that can range from 80 to 120 feet deep, depending on the site and flow conditions, perhaps the point samples collected near the channel bed, with presumably higher SS concentrations, are providing an overestimate of the SS concentration in the lower part of the water column. This in turn could lead to higher overall SS concentrations for the channel cross section compared to depth-integrated methods. Another consideration is that depth-integrated samplers have a lower effective sampling depth than point-integrated samplers (Edwards & Glysson, 1999), meaning depth-integrated methods may not be sampling as much of the lower water column, containing the highest SS concentrations, as point-integrated samplers are able to sample. However, this may be less important in deep rivers like the Mississippi and Atchafalaya Rivers.

Combining records containing both depth- and point-integrated samples may be more problematic for coarse SS compared to fine SS. Previous studies have shown larger variability in concentration and larger differences between field methods for coarse SS compared to fine or total SS (Horowitz et al., 1990). We also saw larger relative bias (%) between sampling methods for coarse SS at several sites. Even though the magnitude of the bias tended to be smaller for coarse than total SS, the fact that coarse SS concentrations were overall lower resulted in a larger percentage difference. This could be why we see big differences between sampling methods at sites around the diversion (MS-UNIO, MS-TARB, and AT-SIMM): they contain larger amounts of coarse SS than the Lower Atchafalaya River sites (Fig. 5). Coarse SS is further problematic because it is difficult to model due to the data representing a Pareto distribution which, depending on the calibration dataset, may not result in normally distributed residuals. This was the issue we had when trying to fit a MLR model for coarse SS at the Old River outflow channel (Table 2).

Because the bias is not consistent across sites, decisions for how to proceed with such datasets also vary by site. For a site like Wax Lake Outlet (AT-WAXL) with optimal site conditions, the results here indicate using the entire dataset, without regard to the sampling method, is appropriate. Incorporating samples collected using both depth- and point-integrated techniques doubles the overall sample count compared to just using depth- or point-integrated samples alone. Higher sample counts across a range of hydrologic conditions typically lead to improved estimates of flux. For a site like Morgan City (AT-MORG), with less-than-optimal site conditions and statistically significant differences due to sampling methods, the answer is more nuanced. For flux calculations, one approach is to “bias-adjust” the samples collected using the less common or historical sampling method. For Morgan City (AT-MORG), this would mean bias-adjusting the point-integrated samples, so they are lower in magnitude and more comparable to the depth-integrated samples. One could use either the proportional bias identified with the MLR equations or the difference between FN estimates identified with WRTDS. Additional testing would help to identify the best adjustment for removing the bias.

From a trend perspective, the above approaches for eliminating the bias are also sufficient. However, there are slight differences in the FN estimates over time (i.e., trend lines) between the sampling methods at both sites, and these differences vary between total, fine, and coarse SS (Online Resource 1, Fig. SI1-4). Trends between the start and end of the analysis period (2007 to 2015) for total and fine SS at Morgan City (AT-MORG) and coarse SS at Wax Lake Outlet (AT-WAXL) show similar rates of decreasing concentrations for both sampling methods, differing by 5% or less (Online Resource 1, Table SI1-5). The total and fine SS trends at Wax Lake Outlet (AT-WAXL) differ by about 10% between the sampling methods, with point-integrated samples showing steeper decreases in SS concentration compared to depth-integrated samples. Finally, the largest divergence in trend patterns between sampling methods is coarse SS at Morgan City (AT-MORG; 28% difference) where point samples show a slight decrease and then level off whereas depth-integrated samples show steeper sustained decreases in concentration (Online Resource 1, Fig. SI1-4). With the exception of the coarse SS trend at Morgan City (AT-MORG), all of these trend iterations show basically the same pattern of consistently decreasing SS concentrations over the analysis period. Small variations between trends can be unreliable and are often not statistically significant. Studies show regression-based techniques like WRTDS are sensitive to changes in the period of record, the addition of new data, and number of samples used during calibration (Chanat et al., 2016; Lee et al., 2017; Oelsner et al., 2017). Differences between trends of + / − 10% due to small alterations of the calibration dataset are not uncommon, and these issues can be even more influential for short analysis periods (Oelsner et al., 2017) like the ones shown here.

For sites with an abrupt change in sampling methods and significant differences (MS-UNIO, MS-TARB, and AT-SIMM; Table 2), an interim and precautionary solution is to bias-adjust the last few years of data and then re-evaluate for a step-change again after additional years of depth-integrated samples have been collected. If no step-change is detectable at a later point, then data could be used without regard to the sampling methods; however, if a step-change is identified, then an adjustment would be desirable. This approach would work equally well for flux or trend estimation. These site-by-site considerations highlight the importance of collecting comparison samples when field method changes are underway and rigorously evaluating those samples to determine the proper course of action for a particular site. Often the effect and solution will not be known until several years of samples have been collected under a variety of hydrologic conditions. A paired sample approach where samples are collected using both field methods sequentially during the same field trip is the ideal dataset (but also the most time-consuming and expensive) to evaluate potential differences and decide the best course of action.

The extent of mixed field method records across the USA is unknown; however, the results presented here have implications for estimating SS loads and understanding the size fractionation of contaminants that readily absorb to sediment. It is likely the bias (when present) between sampling methods leads to larger bias in SS loads compared to SS concentrations because loads are highly sensitive to times in the record when high concentrations are coincident with high flows. The marginal means from the MLR equations (Fig. SI1-2) and the annual estimates from the WRTDS model (Figs. 8 and SI1-3) both show larger bias between sampling methods at higher concentrations and at moderate to high flows. Estimates of SS load are notorious for their high uncertainty and large confidence intervals (e.g., see Lee et al., 2019); thus, attempts to account for additional sources of variability will likely increase the precision of these estimates. Furthermore, Horowitz et al. (1990) also suggest different sampling methods affect estimates of load, not only of SS but also sediment-associated trace elements. Depending on the element and site, Horowitz et al. (1990) found these effects were particularly apparent at locations with increased cross-sectional variability and higher amounts of coarse SS. Although point-integrated sampling was not included in their study, we suspect these same issues are at play at Morgan City (AT-MORG). Point-integrated sampling at this site captures less coarse SS than depth-integrated sampling, which may lead to lower loads of sediment-associated trace elements, such as copper, zinc, lead, chromium, and nickel (Horowitz et al., 1990). Identifying if multiple field methods exist within a single SS record and testing for bias between the methods are essential steps in computing not only SS loads but also loads and SS size fractionation for trace elements.

The variable bias in SS concentrations identified at some sites in this study points to the importance of sampling design and field methodology in an SS monitoring program. Although this study focused on large rivers, many of these issues are relevant for smaller rivers too. An understanding of the heterogeneity of SS concentrations in the channel cross section is important for accurately capturing a representative sample. However, the direction and shape of these relationships between river size, cross sectional heterogeneity, and sampling method bias are unclear. For example, although large rivers may develop vertical stratification (as shown by Moodie et al. (2022) in the Yellow River, China), smaller rivers can be more vertically mixed leading to a more uniform SS cross section and thus likely lowering or eliminating differences between depth- and point-integrated sampling methods. Alternatively, plumes of SS that originate from the bed may influence a larger proportion of the cross section in small rivers compared to large rivers, perhaps increasing the bias between sampling methods. Much can be said about the importance of standardized field methods and consistent equipment across sites and over time; however, technology advances, as does instrumentation, making these changes inevitable. Continued investigation of various types of field method changes across a range of river sizes, geographies, and SS size fractions may lead to more general conclusions about these competing effects.

One final note, the high spatial and temporal variability of SS across a channel cross section compared to other water quality constituents is well established, yet how this variability ultimately affects SS trend results, and their uncertainty is not well understood. Newer sediment-surrogate techniques like laser in situ scattering and transmissometry (LISST-ABS) and acoustic Doppler current profilers (ADCPs) can capture SS variability through time and across the channel. These newer techniques could inform simulation analyses that give insight into the effect high temporal and spatial variability have on trends that are computed using discrete SS samples. Furthermore, such a simulation analysis would allow us to gauge whether the statistically significant differences reported here are meaningful against the highly variable SS concentrations seen in many rivers.

## Conclusions

Across seven sites in the Lower Mississippi and Atchafalaya Rivers, we found point-integrated samples tended to have higher SS concentrations compared to depth-integrated samples. However, this bias was inconsistent in its presence and magnitude across sites. Comparison of the two longest datasets having concurrent depth- and point-integrating sampling for 9-years indicate the site with desirable field conditions had no statistically significant differences in SS concentrations between sampling methods whereas the site with less-than-ideal conditions (i.e., non-uniform flow, non-trapezoidal channel shape, tributary confluence upstream) and higher coarse SS did. With sufficient sampling across a range of flow conditions, sequential and concurrent differences in SS concentrations due to field methods can be tested and adjustments to the data can be made. Several years of data, collected under a variety of hydrologic conditions, would ideally be considered when gauging the effect of field method changes, and adjustments are likely to be site-specific.

Our study highlights the importance of recording field method protocols and equipment at the time of sampling. The lack of this information creates many unknowns about data quality and comparability. Excluding samples from an analysis because of unknown comparability limit the amount of available data, whereas including such samples may increase the noise of the dataset which interferes with the detection of important patterns and the accuracy of estimates derived from the data. At worst, this inclusion may induce a change over time that could be misinterpreted as an environmental change. This work demonstrates the relevance of field method information, especially decades later. Reporting of some sampling information has become more common over time, but more improvement is possible. Ideally, the reporting of field method information would become as routine as collecting the sample itself.

### Supplementary Information

Below is the link to the electronic supplementary material.

Supplementary file1 (DOCX 128 KB)

Supplementary file2 (XLSX 20 KB)

Supplementary file3 (DOCX 861 KB)

Supplementary file4 (PDF 3210 KB)

Supplementary file5 (PDF 1265 KB)

Supplementary file6 (PDF 105 KB)

## Data Availability

The datasets analyzed for the current study are available at https://doi.org/10.5066/P9YK3S9R.

## Declarations

All authors have read, understood, and have complied as applicable with the statement on “Ethical responsibilities of Authors” as found in the Instructions for Authors.

### Disclaimer

Any use of trade, firm, or product names is for descriptive purposes only and does not imply endorsement by the US Government.
