# Supplementary material for: Differing field methods and site conditions lead to varying bias in suspended sediment concentrations in the Lower Mississippi and Atchafalaya Rivers
Source: Environ Monit Assess. 2023 Oct 2;195(11):1260. doi: 10.1007/s10661-023-11836-z (PMC10749891; doi:10.1007/s10661-023-11836-z)
Supplement: Supplementary file 1 — Supplementary file1 (DOCX 128 KB) [file 10661_2023_11836_MOESM1_ESM.docx]

**Differing field methods and site conditions lead to varying bias in suspended sediment concentrations in the Lower Mississippi and Atchafalaya Rivers**

Environmental Monitoring and Assessment

Online Resource 1 – Supporting Tables and Figures

J. Murphy^1^

L. Schafer^2^

S. Mize^3^

^1^U.S. Geological Survey, DeKalb, Illinois, USA; jmurphy@usgs.gov

^2^U.S. Geological Survey, Catonsville, Maryland, USA

^3^U.S. Geological Survey, Baton Rouge, Louisiana, USA


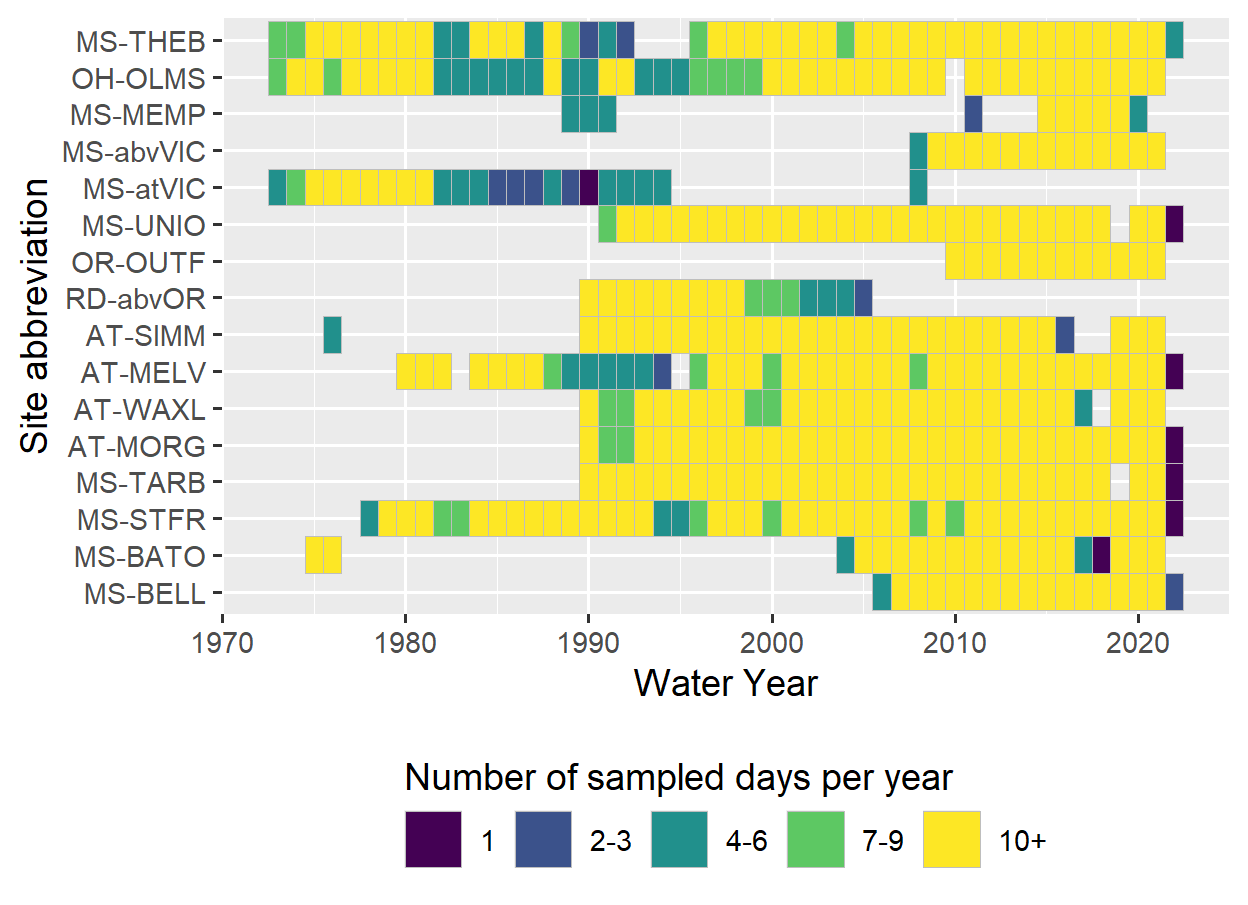
**Fig. SI1-1** Sampling duration and annual frequency of total suspended sediment at 16 sites used in this study. Each rectangle represents a water year (the period from October 1 to September 30 designated by the year in which it ends; water year 2020 was from October 1, 2019, to September 30, 2020) and is filled according to the number days per water year when at least one suspended sediment sample was collected. Data from Murphy et al. (2022). [MS-THEB, Mississippi River at Thebes, IL; OH-OLMS, Ohio River at Olmsted, IL; MS-MEMP, Mississippi River at Memphis, TN; MS-abvVIC, Mississippi River above Vicksburg, MS; MS-atVIC, Mississippi River at Vicksburg, MS; MS-UNIO, Mississippi River at Union Point (Mile 326), LA; MS-TARB, Mississippi River at Tarbert Landing, MS; MS-STFR, Mississippi River near St. Francisville, LA; MS-BATO, Mississippi River at Baton Rouge, LA; MS-BELL, Mississippi River at Belle Chasse, LA; RD-abvOR, Red River above Old River Outflow Channel above Simmesport, LA; OR-OUTF, Old River Outflow Channel below Hydropower Channel; AT-SIMM, Atchafalaya River at Simmesport, LA; AT-MELV, Atchafalaya River at Melville, LA; AT-WAXL, Wax Lake Outlet at Calumet, LA; AT-MORG, Lower Atchafalaya River at Morgan City, LA]

**Table SI1-1** Multiple linear regression equations used in study. [AT-WAXL, Wax Lake Outlet at Calumet, LA; AT-MORG, Lower Atchafalaya River at Morgan City, LA; MS-UNIO, Mississippi River at Union Point, LA; MS-TARB, Mississippi River at Tarbert Landing, MS; OR-OUTF, Old River Outflow Channel below Hydropower Channel, LA; AT-SIMM, Atchafalaya River at Simmesport, LA; log, natural logarithm; conc_mgL, suspended sediment concentration in milligrams per liter; Q, streamflow in cubic feet per second; sample_dt0, time in days since January 1^st^ of the year with the first sample; ltfa, long term flow anomaly; mtfa, medium term flow anomaly; stfa, short term flow anomaly; siteIndex, categorical variable for site, coded as 1 for MS-UNIO and as 0 for MS-TARB; sampling_method, categorical variable for sampling method with “point-integrated” coded a 1 and “depth-integrated” coded as 0; lowQ, categorical variable identifying if the sample was collected at low flows, coded as 0 if Q < 100,000 cfs or coded as 1 if Q >= 100,000 cfs; hyst3, 3-day hysteresis computed as the daily flow minus the mean flow of the previous 3 days]

| Site | Suspended sediment | Model type | Equation |
| --- | --- | --- | --- |
| AT-WAXL | Total | Covariates only | log(conc_mgL)  =  -73.1 + 12.8*log(Q) + -0.521*log(Q)^2^ +  23*stfa + 0.0815*sin(4*pi*sample_dt0) +  0.0338*cos(4*pi*sample_dt0) |
| AT-WAXL | Total | With sampling method | log(conc_mgL)  =  -73.9 + 12.9*log(Q) + -0.526*log(Q)^2^ +  23*stfa + 0.074*sin(4*pi*sample_dt0) +  0.0375*cos(4*pi*smple_dt0) + 0.0623*sampling_method |
| AT-WAXL | Fines | Covariates only | log(conc_mgL)  =  -93.5 + 16.5*log(Q) + -0.689*log(Q)^2^ +  22.3*stfa + 0.0684*sin(4*pi*sample_dt0) +  0.0382*cos(4*pi*sample_dt0) |
| AT-WAXL | Fines | With sampling method | log(conc_mgL)  =  -94.6 + 16.7*log(Q) + -0.698* log(Q)^2^ +   22.3*stfa + 0.0605*sin(4*pi*sample_dt0) +  0.0421*cos(4*pi*sample_dt0) + 0.0656*sampling_method |
| AT-WAXL | Coarse | Covariates only | log(conc_mgL + 1)  =  108 + -20.7*log(Q) + 0.992*log(Q)^2^ +  -483*lowQ + -0.00727*sin(2*pi*sample_dt0) +  0.273*cos(2*pi*sample_dt0) + 19.9*stfa +  80.7*log(Q)*lowQ + -3.37*(log(Q)^2^)*lowQ |
| AT-WAXL | Coarse | With sampling method | log(conc_mgL + 1)  =  106 + -20.4*log(Q) + 0.979*log(Q)^2^ +  -478*lowQ + -0.0115*sin(2*pi*sample_dt0) +  0.274*cos(2*pi*sample_dt0) + 19.9*stfa +  -0.0617*sampling_method + 79.7*log(Q)*lowQ +  -3.32*(log(Q)^2^)* lowQ |
| AT-MORG | Total | Covariates only | log(conc_mgL)  =  -39.5 + 6.84*log(Q) + -0.26*log(Q)^2^ +  27.6*stfa + 0.116*sin(4*pi*sample_dt0) +  0.0448*cos(4*pi*sample_dt0) + -0.675*hyst3 |
| AT-MORG | Total | With sampling method | log(conc_mgL)  =  -40.5 + 7*log(Q) + -0.267* log(Q)^2^ +  28.1*stfa + 0.103*sin(4*pi*sample_dt0) +  0.054*cos(4*pi*sample_dt0) +  -0.811*hyst3 + 0.153*sampling_method |
| AT-MORG | Fines | Covariates only | log(conc_mgL)  =  -62.1 + 10.9*log(Q) + -0.446*log(Q)^2^ + 22.4*stfa + 0.0953*sin(4*pi*sample_dt0) +  0.0714*cos(4*pi*sample_dt0) |
| AT-MORG | Fines | With sampling method | log(conc_mgL)  =  -62.8 + 11.1*log(Q) + -0.451*log(Q)^2^ +  22.3*stfa + 0.0879*sin(4*pi*sample_dt0) +  0.0767*cos(4*pi*sample_dt0) + 0.0792*sampling_method |
| AT-MORG | Coarse | Covariates only | log(conc_mgL + 1)  =  0.255 + 0.0307*log(Q) + -36.5*lowQ +  11.9*stfa + 0.197*sin(2*pi*sample_dt0) +  0.342*cos(2*pi*sample_dt0) + 3.21*log(Q)*lowQ |
| AT-MORG | Coarse | With sampling method | log(conc_mgL + 1)  =  0.732 + -0.0261*log(Q) + -36.8*lowQ +  11.3*stfa + 0.22*sin(2*pi*sample_dt0) +  0.341*cos(2*pi*sample_dt0) + 0.291*sampling_method +  3.24*log(Q)*lowQ |
| MS-UNIO  MS-TARB | Total | Covariates only | log(conc_mgL)  =  -138 + 0.123*siteIndex + 21.6*log(Q) +  -0.816* log(Q)^2^ + 33.3*stfa |
| MS-UNIO MS-TARB | Total | With sampling method | log(conc_mgL)  =  -150 + 0.106*siteIndex + 23.3*log(Q) +  -0.874* log(Q)^2^ + 31.5*stfa + 0.342*sampling_method |
| MS-UNIO  MS-TARB | Fines | Covariates only | log(conc_mgL)  =  -148 + 0.152*siteIndex + 23.3*log(Q) +  -0.886* log(Q)^2^ + 35.5*stfa |
| MS-UNIO  MS-TARB | Fines | With sampling method | log(conc_mgL)  =  -157 + 0.138*siteIndex + 24.6*log(Q) +  -0.931*log(Q)^2^ + 34.2*stfa + 0.262*sampling_method |
| MS-UNIO  MS-TARB | Coarse | Covariates only | conc_mgL  =  -30.9 + -1.21*siteIndex + 0.000154*Q +  -7.62e-11*Q^2^ + -2.57*sin(2*pi*sample_dt0) +  16.5*cos(2*pi*sample_dt0) + -4090*ltfa + 232000*ltfa^2^ |
| MS-UNIO  MS-TARB | Coarse | With sampling method | conc_mgL  =  -43.8 + -0.468*siteIndex + 0.000165*Q +  -8.27e-11*Q^2^ + -2.6*sin(2*pi*sample_dt0) +  15.8*cos(2*pi*sample_dt0) + -2760*ltfa +  182000*ltfa^2^ + 12.1*sampling_method |
| OR-OUTF | Total | Covariates only | log(conc_mgL)  =  266 + -71.1*log(Q) + 6.42* log(Q)^2^ +  -0.192*log(Q)^3^ + 23.1*stfa |
| OR-OUTF | Total | With sampling method | log(conc_mgL)  =  266 + -71.3*log(Q) + 6.44*log(Q)^2^ +  -0.193* log(Q)^3^ + 23.1*stfa + 0.0176*sampling_method |
| OR-OUTF | Fines | Covariates only | log(conc_mgL)  =  331 + -89.3*log(Q) + 8.1* log(Q)^2^ +  -0.244* log(Q)^3^ + 21*stfa |
| OR-OUTF | Fines | With sampling method | log(conc_mgL)  =  334 + -90*log(Q) + 8.16*log(Q)^2^ +  -0.245*log(Q)^3^ + 20.8*stfa + 0.0558*sampling_method |
| AT-SIMM | Total | Covariates only | log(conc_mgL)  =  -107 + 17.8*log(Q) + -0.71*log(Q)^2^ +  24.6*stfa |
| AT-SIMM | Total | With sampling method | log(conc_mgL)  =  -103 + 17*log(Q) + -0.675*log(Q)^2^ +  21.9*stfa + 0.197*sampling_method |
| AT-SIMM | Fines | Covariates only | log(conc_mgL)  =  910 + -232*log(Q) + 19.7*log(Q)^2^ +  -0.558*log(Q)^3^ + 21*stfa |
| AT-SIMM | Fines | With sampling method | log(conc_mgL)  =  678 + -176*log(Q) + 15.1*log(Q)^2^ +  -0.434* log(Q)^3^ + 19.1*stfa + 0.162*sampling_method |
| AT-SIMM | Coarse | Covariates only | log(conc_mgL + 1)  =  -164 + 25*log(Q) + -0.933* log(Q)^2^ +  0.145*sin(2*pi*sample_dt0) + 0.251*cos(2*pi*sample_dt0) +  26.8*stfa |
| AT-SIMM | Coarse | With sampling method | log(conc_mgL + 1)  =  -164 + 25*log(Q) + -0.932*log(Q)^2^ +  0.13*sin(2*pi*sample_dt0) + 0.26*cos(2*pi*sample_dt0) +  26*stfa + 0.0442*sampling_method |

**Table SI1-2** Outliers removed during multiple linear regression (MLR) modeling [AT-WAXL, Wax Lake Outlet at Calumet, LA; AT-MORG, Lower Atchafalaya River at Morgan City, LA; MS-UNIO, Mississippi River at Union Point, LA; OR-OUTF, Old River Outflow Channel below Hydropower Channel, LA; AT-SIMM, Atchafalaya River at Simmesport]

| Site | Sample fraction | Sample date |
| --- | --- | --- |
| AT-WAXL | Total | 8/16/2007 |
|  |  | 6/12/2008 |
|  | Fines | 8/16/2007 |
|  |  | 6/12/2008 |
|  | Coarse | 6/12/2008 |
| AT-MORG | Total | 9/25/2008 |
|  |  | 1/28/2016 |
| MS-UNIO | Coarse | 11/1/2016 |
| OR-OUTF | Total | 8/4/2021 |
|  | Fines | 8/4/2021 |
| AT-SIMM | Coarse | 5/6/2021 |


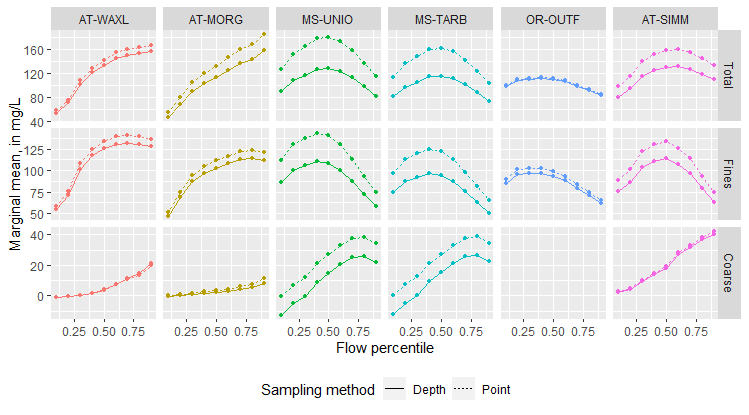
**Fig. SI1-2** Marginal means estimated from multiple linear regression (MLR) simulations, by sampling method and site, versus flow percentiles (0.10 through 0.90, in increments of 0.10). [AT-WAXL, Wax Lake Outlet at Calumet, LA; AT-MORG, Lower Atchafalaya River at Morgan City, LA; MS-UNIO, Mississippi River at Union Point, LA; MS-TARB, Mississippi River at Tarbert Landing, MS; OR-OUTF, Old River Outflow Channel below Hydropower Channel, LA; AT-SIMM, Atchafalaya River at Simmesport, LA]

**
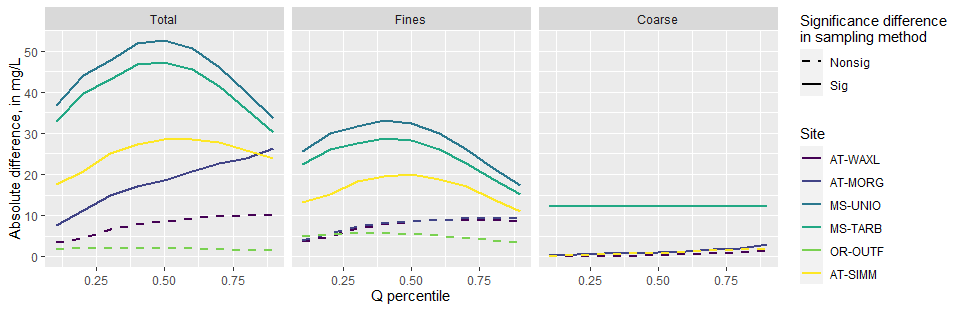
**

**Fig. SI1-3** Absolute difference in marginal means in milligrams per liter (mg/L) between point- and depth-integrated samples plotted versus flow percentiles (Q percentile). Significance was assessed using the p value of the coefficient on the 2-level sampling method categorical variable and an alpha level of 0.05. [AT-WAXL, Wax Lake Outlet at Calumet, LA; AT-MORG, Lower Atchafalaya River at Morgan City, LA; MS-UNIO, Mississippi River at Union Point, LA; MS-TARB, Mississippi River at Tarbert Landing, MS; OR-OUTF, Old River Outflow Channel below Hydropower Channel, LA; AT-SIMM, Atchafalaya River at Simmesport, LA]

**Table SI1-3** Results from nonparametric tests. Wilcoxon Signed Rank test used for paired sample data and Wilcoxon Rank Sum test used for unpaired (grouped) data. [PI, point-integrated samples; DI, depth-integrated samples; mg/L, milligrams per liter; AT-WAXL, Wax Lake Outlet at Calumet, LA; AT-MORG, lower Atchafalaya River at Morgan City, LA; MS-BELL, Mississippi River at Belle Chasse, LA; OR-OUTF, Old River Outflow Channel below Hydropower Channel, LA; concs, concentrations; Seq, Sequential field methods; p value from Wilcoxon test bolded if < 0.05]

|  | Site and data | Suspended sediment | n paired samples or samples per group (*2 for total n) | p value | Median difference  (PI – DI), in mg/L |
| --- | --- | --- | --- | --- | --- |
| Concurrent field methods | AT-WAXL paired concs | Total | 18 | 0.61 | -1.5 |
|  |  | Fine | 17 | 0.44 | -2.0 |
|  |  | Coarse | 17 | 0.14 | -1.0 |
|  | AT-MORG paired concs | Total | 20 | **<0.01** | **15** |
|  |  | Fine | 19 | 0.06 | 7.5 |
|  |  | Coarse | 19 | **0.02** | **2.5** |
|  |  |  |  |  | Difference in medians |
|  | MS-BELL concs | Total | 12 | 0.62 | 16 |
|  |  | Fine | 12 | 0.91 | 2.2 |
|  |  | Coarse | 12 | 0.49 | 5.7 |
|  | MS-BELL flow-adjusted concs | Total | 12 | 0.75 | 4.7 |
|  |  | Fine | 12 | 0.84 | 4.1 |
|  |  | Coarse | 12 | 0.62 | -0.51 |
| Seq | OR-OUTF concs | Coarse | 51 | 0.11 | -3.0 |
|  | OR-OUTF flow-adjusted concs | Coarse | 51 | 0.59 | 0.12 |

**Table SI1-4** Weighted regressions on time, discharge, and season (WRTDS) model error statistics. [LogC, logarithm of concentration; LogF, logarithm of flux (load); RMSE, root mean square error for LogC; SepPercent, standard error of prediction for concentration, as a percent; AT-WAXL, Wax Lake Outlet at Calumet, LA; AT-MORG, Lower Atchafalaya River at Morgan City, LA]

| Site | Sampling Method | Sediment Fraction | R^2^ LogC | R^2^ LogF | RMSE | SepPercent |
| --- | --- | --- | --- | --- | --- | --- |
| AT-WAXL | Depth | Total | 0.515 | 0.821 | 0.463 | 48.9 |
|  |  | Fines | 0.42 | 0.78 | 0.485 | 51.5 |
|  |  | Coarse | 0.913 | 0.947 | 0.508 | 54.2 |
|  | Point | Total | 0.724 | 0.911 | 0.347 | 35.8 |
|  |  | Fines | 0.64 | 0.887 | 0.37 | 38.3 |
|  |  | Coarse | 0.876 | 0.925 | 0.631 | 69.9 |
| AT-MORG | Depth | Total | 0.659 | 0.898 | 0.365 | 37.8 |
|  |  | Fines | 0.555 | 0.868 | 0.379 | 39.3 |
|  |  | Coarse | 0.819 | 0.888 | 0.789 | 93.0 |
|  | Point | Total | 0.687 | 0.900 | 0.384 | 39.9 |
|  |  | Fines | 0.499 | 0.844 | 0.426 | 44.6 |
|  |  | Coarse | 0.783 | 0.859 | 0.981 | 127 |


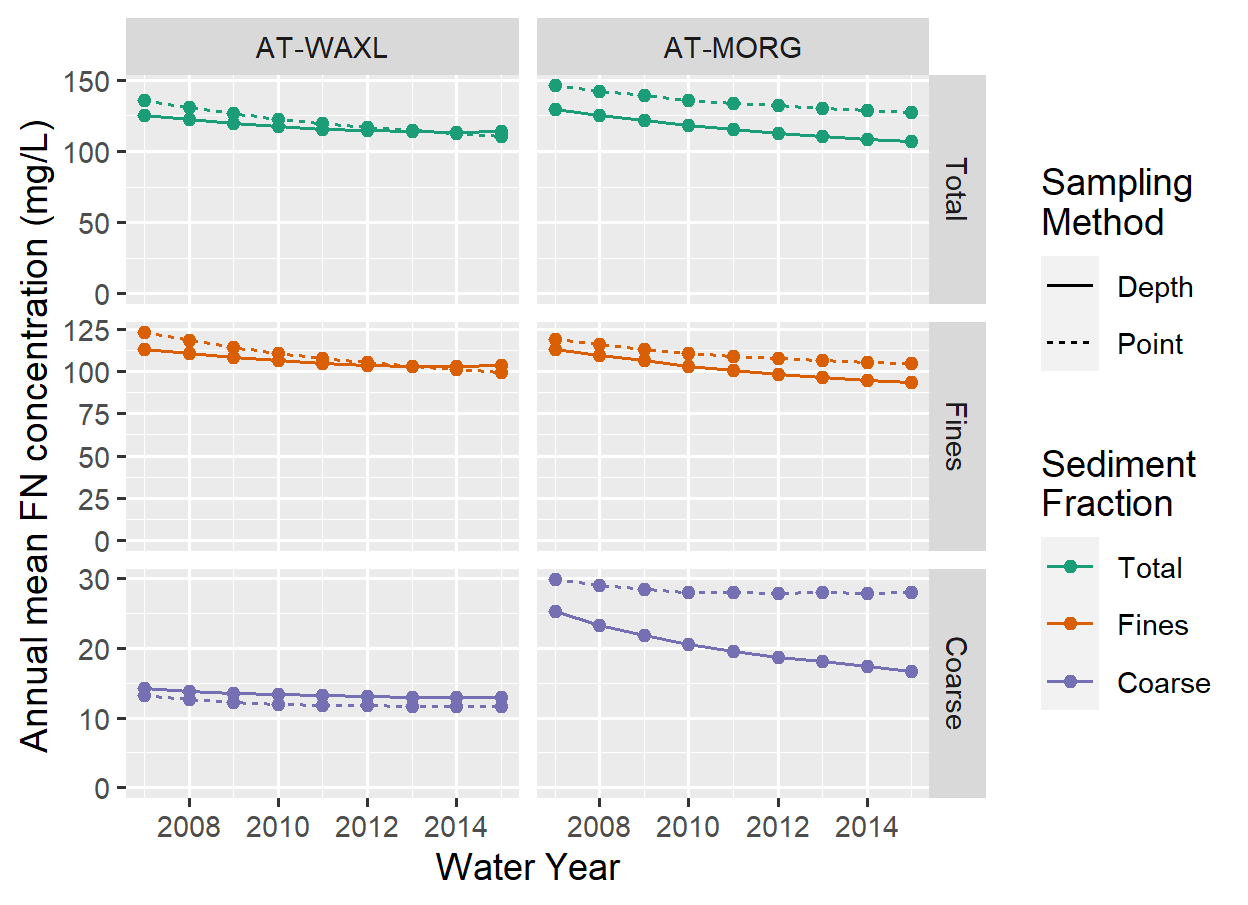


**Fig. SI1-4** Weighted regressions on time, discharge, and season (WRTDS) flow normalized (FN) concentrations in milligrams per liter (mg/L) vs. time plot (i.e., annual trends in mean suspended sediment concentration), by site, sediment fraction and sampling method. [AT-WAXL, Wax Lake Outlet at Calumet, LA; AT-MORG, Lower Atchafalaya River at Morgan City, LA]

**Table SI1-5** Weighted regressions on time, discharge, and season (WRTDS) trend results and comparisons. Trend computed as the annual estimate for water year 2015 minus water year 2007 (i.e., ending WY estimate minus beginning WY estimate; a water year is the period from October 1 to September 30 designated by the year in which it ends; water year 2020 was from October 1, 2019, to September 30, 2020). Trend as percent change is the trend divided by the starting estimate in WY 2007. Trend difference is calculated as the point-integrated trend estimate minus depth-integrated trend estimate. [mg/L, milligrams per liter; AT-WAXL, Wax Lake Outlet at Calumet, LA; AT-MORG, Lower Atchafalaya River at Morgan City, LA]

| **Site** | **Suspended Sediment** | **Sampling method** | **Trend, in mg/L** | **Trend, in percent change** | **Trend difference, in mg/L** | **Trend difference, in percent** |
| --- | --- | --- | --- | --- | --- | --- |
| AT-WAXL | Total | Depth | -11.0 | -9.0 | -15.0 | -10.0 |
| AT-WAXL | Total | Point | -26.0 | -19.0 |  |  |
| AT-WAXL | Fines | Depth | -9.5 | -8.4 | -14.0 | -11.0 |
| AT-WAXL | Fines | Point | -24.0 | -19.0 |  |  |
| AT-WAXL | Coarse | Depth | -1.3 | -9.3 | -0.2 | -2.7 |
| AT-WAXL | Coarse | Point | -1.5 | -12.0 |  |  |
| AT-MORG | Total | Depth | -23.0 | -17.0 | 4.0 | 4.0 |
| AT-MORG | Total | Point | -19.0 | -13.0 |  |  |
| AT-MORG | Fines | Depth | -20.0 | -17.0 | 6.0 | 5.0 |
| AT-MORG | Fines | Point | -14.0 | -12.0 |  |  |
| AT-MORG | Coarse | Depth | -8.5 | -34.0 | 6.6 | 28.0 |
| AT-MORG | Coarse | Point | -1.9 | -6.3 |  |  |

**References**

Murphy, J. C., Mize, S. V., Swarzenski, C. M., and Schafer, L. A. (2022). Datasets of suspended sediment concentration and percent fines (1973–2021), sampling information (1973–2021), and daily streamflow (1928–2021) for sites in the Lower Mississippi and Atchafalaya Rivers to support analyses of sediment transport and delivery. *U.S. Geological Survey data release*. <https://doi.org/10.5066/P9YK3S9R>
