# Supplementary material for: Differing field methods and site conditions lead to varying bias in suspended sediment concentrations in the Lower Mississippi and Atchafalaya Rivers
Source: Environ Monit Assess. 2023 Oct 2;195(11):1260. doi: 10.1007/s10661-023-11836-z (PMC10749891; doi:10.1007/s10661-023-11836-z)
Supplement: Supplementary file 3 — Supplementary file3 (DOCX 861 KB) [file 10661_2023_11836_MOESM3_ESM.docx]

**Differing field methods and site conditions lead to varying bias in suspended sediment concentrations in the Lower Mississippi and Atchafalaya Rivers**

Environmental Monitoring and Assessment

Online Resource 3 – Summary of daily streamflow record compilation

J. Murphy^1^

L. Schafer^2^

S. Mize^3^

^1^U.S. Geological Survey, DeKalb, Illinois, USA; jmurphy@usgs.gov

^2^U.S. Geological Survey, Catonsville, Maryland, USA

^3^U.S. Geological Survey, Baton Rouge, Louisiana, USA

# Scope and Background

This document summarizes how we compiled daily streamflow records for each of the 16 sediment sites in the Lower Mississippi/Atchafalaya River Basin that are used in this study. The hyperlinks in Table 1 provide more information about the computations, estimation methods, or source data used to compile these records. The compiled streamflow data are available in Murphy et al. (2022). Note, siteIndex and gageIndex are the primary location identifiers for suspended sediment and streamflow data, respectively. Each siteIndex is paired with a gageIndex, and each of these may include data from one or more U.S. Geological Survey (USGS) or U.S. Army Corps of Engineers (USACE) sites. See Table 1 in the manuscript and siteTable.csv in Murphy et al. (2022), for site and streamgage names and more information.

Murphy, J. C., Mize, S. V., Swarzenski, C. M., and Schafer, L. A., 2022, Datasets of suspended sediment concentration and percent fines (1973–2021), sampling information (1973–2021), and daily streamflow (1928–2021) for sites in the Lower Mississippi and Atchafalaya Rivers to support analyses of sediment transport and delivery: U.S. Geological Survey data release, <https://doi.org/10.5066/P9YK3S9R>.

# Summary

Within the streamflow dataset (Murphy et al., 2022), the main organizing column (i.e., location identifier) of the daily streamflow data is “gageIndex.” Some gages use data from multiple sources (e.g., U.S. Geological Survey [USGS] and U.S. Army Corps of Engineers [USACE]), some include estimated streamflows (using a variety of techniques), and some use computed values (i.e., addition or subtraction from upstream or downstream gages). Table 1 below shows the siteIndex-gageIndex pairings along with notes about data compilation and estimation. Monitored streamflow data were retrieved from three sources: USGS NWISweb via API retrieval, USACE rivergages.com webpage manual retrievals, and emailed comma-separated value (CSV) files from James Lewis (former Director of USACE Mississippi River Science and Technology Office) in September and October of 2021. Most of the streamgages use USGS data, a handful use USACE data, and a few use primarily USGS data with USACE data used to fill long gaps.


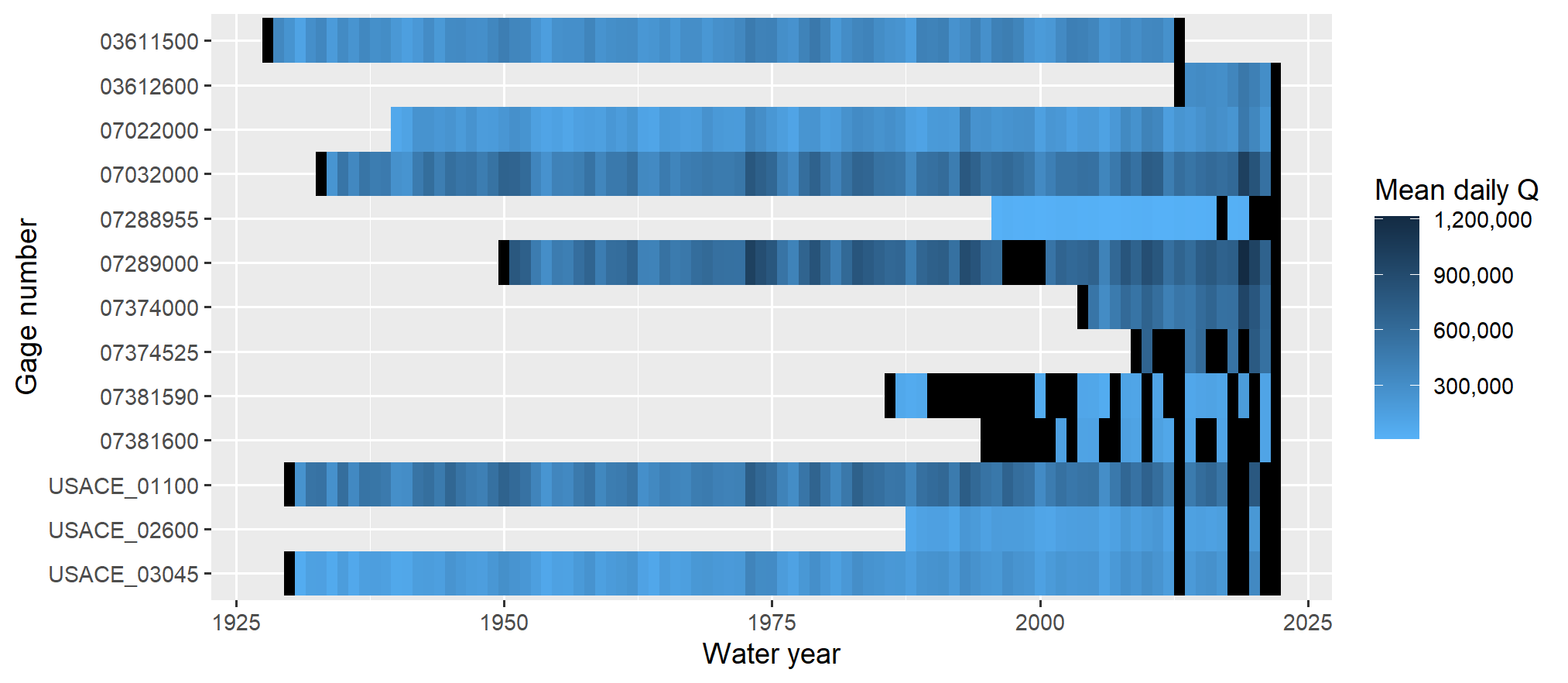


Figure SI3-1. Annual mean daily streamflow (Q) in cubic feet per second for gages used to compile continuous daily streamflow for the 16 study sites. Black boxes indicate a year with one or more days of missing data. Data from Murphy, J. C., Mize, S. V., Swarzenski, C. M., and Schafer, L. A., 2022, Datasets of suspended sediment concentration and percent fines (1973–2021), sampling information (1973–2021), and daily streamflow (1928–2021) for sites in the Lower Mississippi and Atchafalaya Rivers to support analyses of sediment transport and delivery: U.S. Geological Survey data release, <https://doi.org/10.5066/P9YK3S9R>.


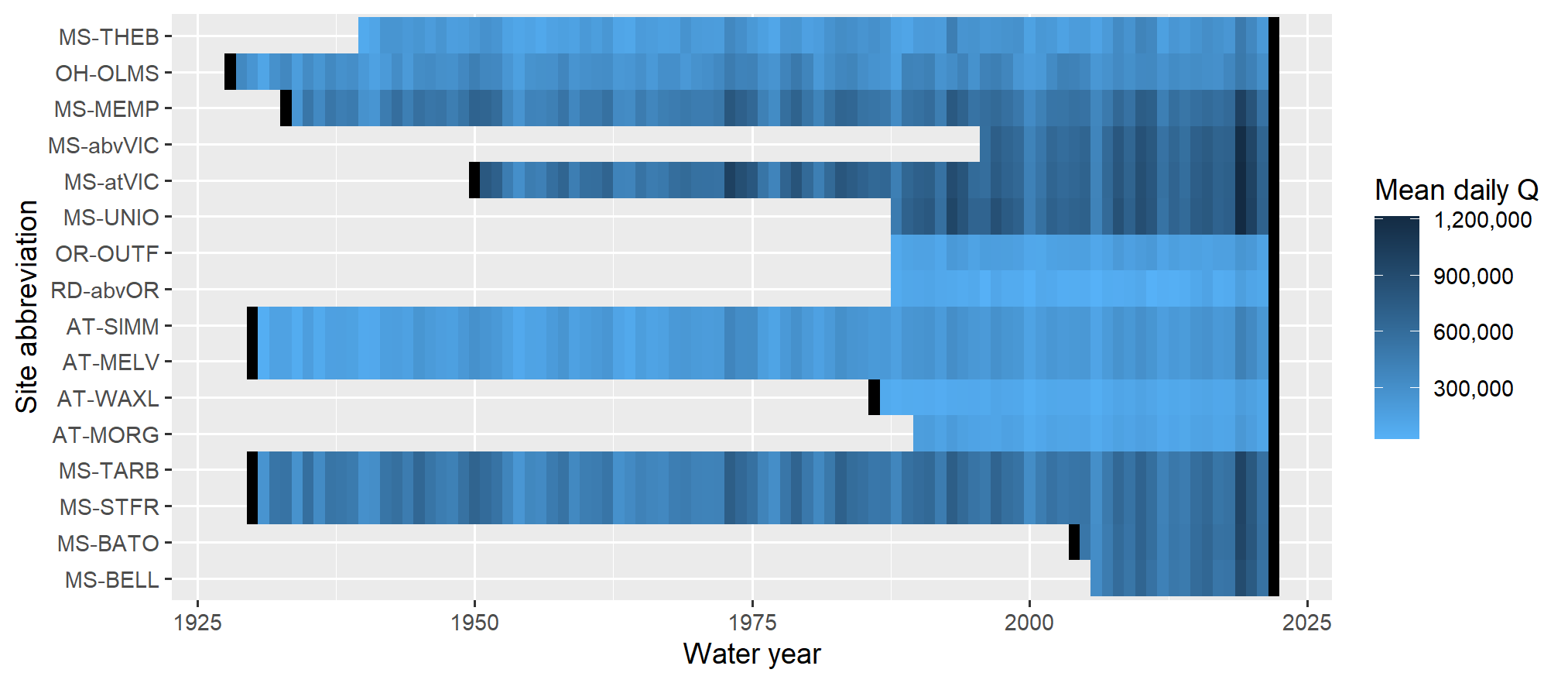


Figure SI3-2. Annual mean daily streamflow (Q) in cubic feet per second, compiled, cleaned, and processed for each of the 16 study sites. There are no missing days in these periods of records (except for partial years at some sites at the very beginning and end of the record). Data from Murphy, J. C., Mize, S. V., Swarzenski, C. M., and Schafer, L. A., 2022, Datasets of suspended sediment concentration and percent fines (1973–2021), sampling information (1973–2021), and daily streamflow (1928–2021) for sites in the Lower Mississippi and Atchafalaya Rivers to support analyses of sediment transport and delivery: U.S. Geological Survey data release, <https://doi.org/10.5066/P9YK3S9R>.

Table SI3-1. siteIndex (suspended sediment site) and gageIndex (streamflow gage) pairings for 16 study sites in the Lower Mississippi and Atchafalaya Rivers, plus information about the U.S. Geological Survey (USGS) and U.S. Army Corps of Engineers (USACE) streamflow gages used to compile the daily records, the number of days streamflows were estimated, and the complete period of record in water years (WY; the period from October 1 to September 30 designated by the year in which it ends; water year 2020 was from October 1, 2019, to September 30, 2020). See Table 1 in the manuscript for suspended sediment site names and numbers (siteIndex).

| **siteIndex \| gageIndex**  USGS or USACE gage numbers and additional information about record compilation | **Flows (days) computed using other gages** | **Sporadic missing flows estimated using time series model*** | **Complete period of record, in water years**  **(number of water years)** |
| --- | --- | --- | --- |
| **MS-THEB \| MS-THEB**  USGS gage 07022000 | --- | --- | 1939-10-01 to 2021-09-30 (83) |
| **OH-OLMS \|** [**OH-OLMSqx**](#_Mississippi_River_@)  Ohio River at Olmsted (03612600) from 2013-03-01 to present. Historical bias-corrected streamflow from Ohio River at Metropolis (03611500) prior to 2013-03-01. | --- | --- | 1928-10-01 to 2021-09-30 (94) |
| **MS-MEMP \|** [**MS-MEMPqx**](#_Mississippi_River_at)  Mississippi River at Memphis (07032000) with additional data from nearby USACE gage (written communication^#^) | --- | --- | 1933-10-01 to 2021-09-30 (89) |
| **MS-abvVIC \|** [**MS-abvVICqx**](#_Mississippi_River_above_1) Computed as Mississippi River above Vicksburg (07289000) minus Yazoo River (07288955) | All | **  51 (Yazoo) | 1995-10-01 to 2021-09-30 (27) |
| **MS-atVIC \|** [**MS-atVICqx**](#_Mississippi_River_@_1)  Mississippi River at Vicksburg (07289000) from 2008-01-01 to present with historical data from USACE (written communication^#^) | --- | 53 | 1950-10-01 to 2021-09-30 (72) |
| **MS-UNIO \| MS-UNIOqx**  Computed as Mississippi River at Tarbert Landing (USACE gage 01100) plus Old River Outflow Channel (USACE gage 02600) | All | ** | 1987-10-01 to 2021-09-30 (35) |
| **MS-TARB \| MS-TARB**  **MS-STFR \| MS-TARB**  Two siteIndexs use same gageIndex. Mississippi River at Tarbert Landing (USACE gage 01100) with long gaps filled with USACE data from written communication^#^ | --- | 21 | 1930-10-01 to 2021-09-30 (92) |
| **MS-BATO \| MS-BATO**  Mississippi River at Baton Rouge (07374000) | --- | 1 | 2004-10-01 to 2021-09-30 (18) |
| **MS-BELL \|** [**MS-BELLqx**](#_Mississippi_River_@_2)  Mississippi River at Belle Chasse (07374525), with historical data estimated using daily flow ratios and Baton Rouge flow | 1,123 (19%)  All values prior to ~WY 2009 | 35 | 2005-10-01 to 2021-09-30 (17) |
| **Red River \| RD-abvORqx** Computed as Atchafalaya River at Simmesport (USACE gage 03045) minus Old River Outflow Channel (USACE gage 02600) | All | ** | 1987-10-01 to 2021-09-30 (35) |
| **OR-OUTF \| OR-OUTF**  Old River Outflow Channel (USACE gage 02600), with long gaps filled with USACE data from written communication^#^ | --- | 18 | 1987-10-01 to 2021-09-30 (35) |
| **AT-SIMM \| AT-SIMM**  **AT-MELV \| AT-SIMM**  Two siteIndexs use same gageIndex. Atchafalaya River at Simmesport (USACE gage 03045), long gaps filled with USACE data from written communication^#^ | --- | 19 | 1930-10-01 to 2021-09-30 (92) |
| **AT-WAXL \|** [**AT-WAXLqx**](#_Atchafalaya_River_@)  Atchafalaya River at Wax Lake Outlet (07381590), with missing days estimated using daily flow ratios and Simmesport flow | 705 (6%) | 1 | 1986-10-01 to 2021-09-30 (36) |
| **AT-MORG \|** [**AT-MORGqx**](#_Atchafalaya_River_@_2)  Atchafalaya River at Morgan City (07381600), with historical data and gaps estimated using daily flow ratios and Simmesport flow | 9,132 (28%)  All values prior to WY 2006 | --- | 1989-10-01 to 2021-09-30 (33) |

*Number of missing days after initial estimations or computations described underneath the siteIndex | gageIndex pairings in the far-left column. These missing flows were filled using a structural time series model where the estimated series was smoothed using a state-space model. Implemented using the fillMiss function in the waterData R package (Ryberg K.R., and Vecchia A.V. (2017). waterData: Retrieval, Analysis, and Anomaly Calculation of Daily Hydrologic Time Series Data. R package version 1.0.8, <https://CRAN.R-project.org/package=waterData>).

**Records computed after missing values were filled.

^#^Written communication is USACE streamflow data emailed to Jennifer Murphy from James Lewis (former Director of the Mississippi River Science and Technology Office) in September and October 2021.

# Ohio River @ Olmsted (OH-OLMS | OH-OLMSqx)

The Ohio River at Olmsted site uses streamflow data from the Ohio River at Metropolis (03611500) from 1929 to 2013-02-28 and streamflow data from the Ohio River at Olmsted (03612600) from 2013-03-01 to present. Historical streamflow data from the Metropolis gage were bias corrected.

**Bias:** Streamflow data from both locations overlap from 2013-03-01 to 2015-01-12. Difference in daily streamflow during this period shows daily streamflows at Metropolis were ~9% lower than Olmsted, on average, with differences ranging from -23% to 38%.

**Bias correction:** Using the data from the overlapping period, we calculated the difference in streamflow between Olmsted and Metropolis (Olmsted - Metropolis) and regressed this difference against the log of streamflow at Metropolis and season (sine and cosine terms on decimal time) because there appeared to be seasonal variability in the bias. This “bias correction factor (BCF) model” had a p value < 0.01, all coefficients had p values < 0.01, and the coefficient of determination (r^2^) was 0.26. When the estimated BCFs are added to the daily streamflow data at Metropolis during the overlapping period, the mean difference between streamflow at Olmsted and Metropolis+BCF was 0 cubic feet per second (cfs) and the mean percent difference was <1%. Plots below show bias corrected data from Metropolis compared to Olmsted. The plot on the bottom right still shows some seasonality in the bias after correction but not as strong as without the correction.

The fitted BCF model was used to estimate a BCF for each day of the historical Metropolis data, and these BCFs were then added to the corresponding daily streamflow. The BCF was not applied to daily streamflows that were less than the lowest observed daily streamflow at Metropolis during the overlapping period used to develop the BCF model. These low flows (<55,000 cfs) were left as-is, with no BCF added.

Figure SI3-3. Top graph shows daily flow (Q) in cubic feet per second (cfs) versus Date where the salmon line is streamflow at Metropolis and the blue line is streamflow at Olmsted. Bottom left graph shows daily streamflow in cfs at Olmsted (Q_03612600) versus daily streamflow in cfs at Metropolis (Q_03611500). Bottom right boxplot shows percent difference (pctDiff) versus month of the year where “1” is January. For the boxplots, the bottom, middle, and top of the box represent the 25^th^ percentile, median value, 75^th^ percentile. Top and bottom whiskers extend to the largest or smallest value or no farther than +/- 1.5*IQR (interquartile range). Solid black circles are outliers beyond +/- 1.5*IQR.


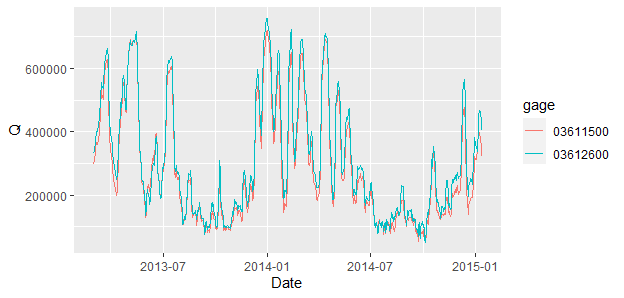

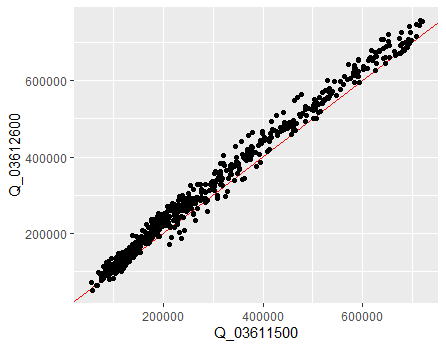

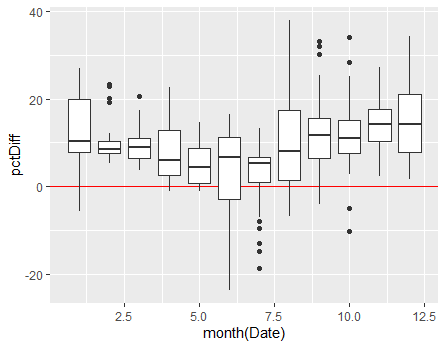

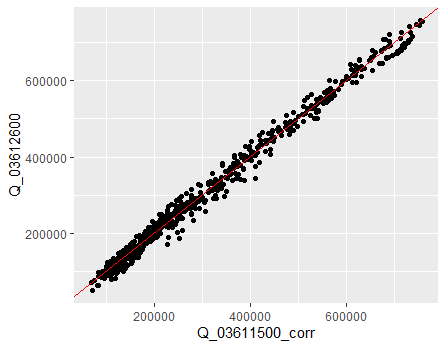

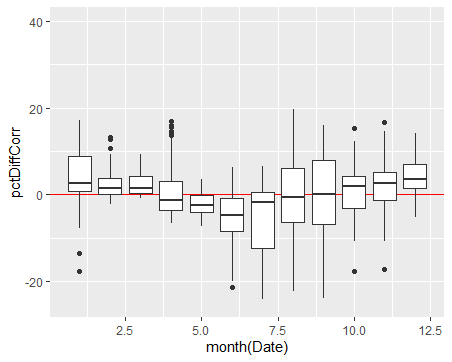

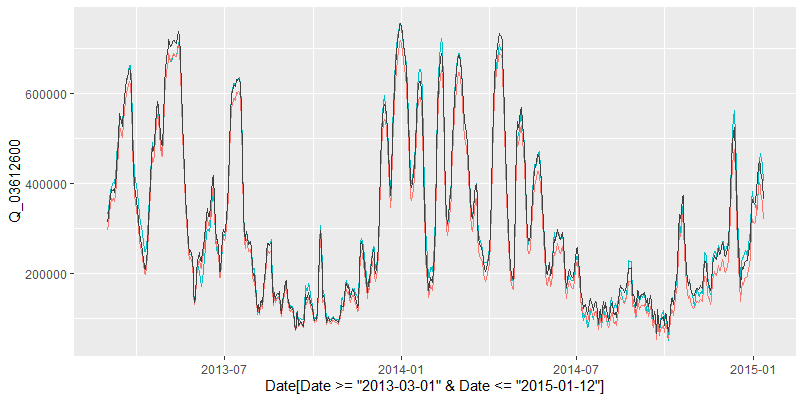


Figure SI3-4. Top graph shows daily flow in cubic feet per second (cfs) versus Date where the salmon line is streamflow at Metropolis, the blue line is streamflow at Olmsted, and the black line is bias-corrected streamflow at Metropolis. Bottom left graph shows daily streamflow in cfs at Olmsted (Q_03612600) versus bias-corrected streamflow in cfs at Metropolis (Q_03611500_corr). Bottom right boxplot shows percent difference between the bias corrected Metropolis streamflow and streamflow at Olmsted (pctDiffCorr) versus month of the year where “1” is January. For the boxplots, the bottom, middle, and top of the box of represent the 25^th^ percentile, median value, 75^th^ percentile, respectively. Top and bottom whiskers extend to the largest or smallest value or no farther than +/- 1.5*IQR (interquartile range). Solid black circles are outliers beyond +/- 1.5*IQR.

# Mississippi River @ Memphis (MS-MEMP | MS-MEMPqx)

Streamflow data at Memphis are a combination of USGS data and USACE data. USGS data are used from the beginning of the record through water year (WY) 1994, then USACE data provided via written communication are used WY 1995 through WY 2014, and USGS data are used again from WY 2015 through the end of the record. USACE data were provided to Jennifer Murphy (USGS) as CSV files via email from James Lewis (U.S. Army Corps of Engineers, former Director of Mississippi River Science and Technology Office).


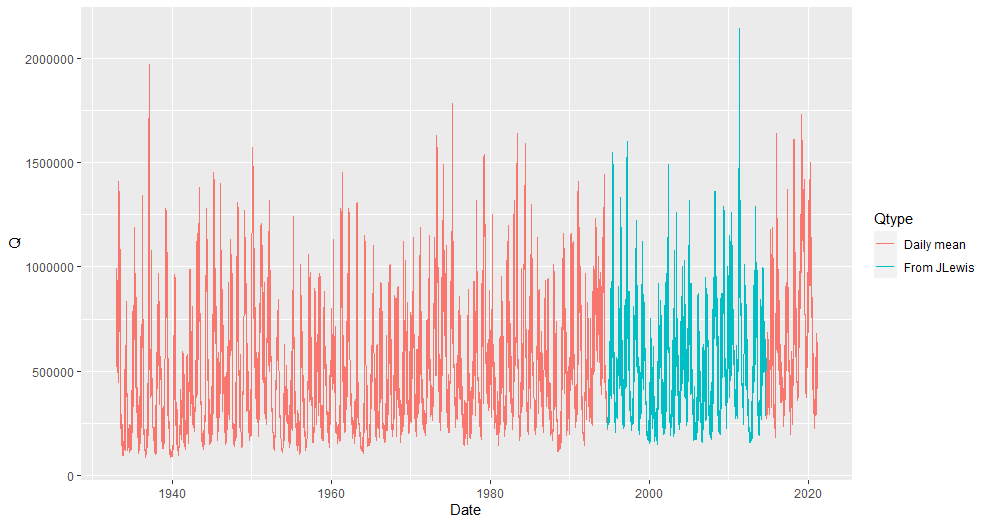


Figure SI3-5. Daily mean streamflow data (Q) in, in cubic feet per second at Memphis where salmon line is daily mean streamflow provided by the U.S. Geological Survey and the blue line is daily streamflow provided by U.S. Army Corps of Engineers as written communications. Data from Murphy, J. C., Mize, S. V., Swarzenski, C. M., and Schafer, L. A., 2022, Datasets of suspended sediment concentration and percent fines (1973–2021), sampling information (1973–2021), and daily streamflow (1928–2021) for sites in the Lower Mississippi and Atchafalaya Rivers to support analyses of sediment transport and delivery: U.S. Geological Survey data release, <https://doi.org/10.5066/P9YK3S9R>.

#

# Mississippi River above Vicksburg (MS-abvVIC | MS-abvVICqx)

Suspended sediment data are available from mid-2008 through water year (WY) 2021 at the Mississippi River above Vicksburg site (MS-abvVIC; 322023090544500); however, no streamflow data have been collected at this site. This sediment site is located directly upstream from the Mississippi River at Vicksburg site (MS-atVIC; 07289000); however, the Yazoo River joins the Mississippi River between these locations. Estimates of daily streamflow for MS-abvVIC were computed by subtracting the daily streamflow at the Yazoo River gage (07288955) from the daily streamflow at the MS-atVIC (07289000). Using this approach, an estimated daily streamflow record is available from WY 1996 through WY 2021.

# Mississippi River @ Vicksburg (MS-atVIC)

Streamflow data at Vicksburg are a combination of USGS and USACE data. USACE data from CSV files provided by James Lewis (former Director of the U.S. Army Corps of Engineers’ Mississippi River Science and Technology Office) in September and October 2021 via email were used for the early part of the record, calendar years 1950 through 2007. After this time, USGS data are used through the end of the record. An additional 53 days were estimated using smoothed estimates from a structural time series model (see Table SI3-1 above).


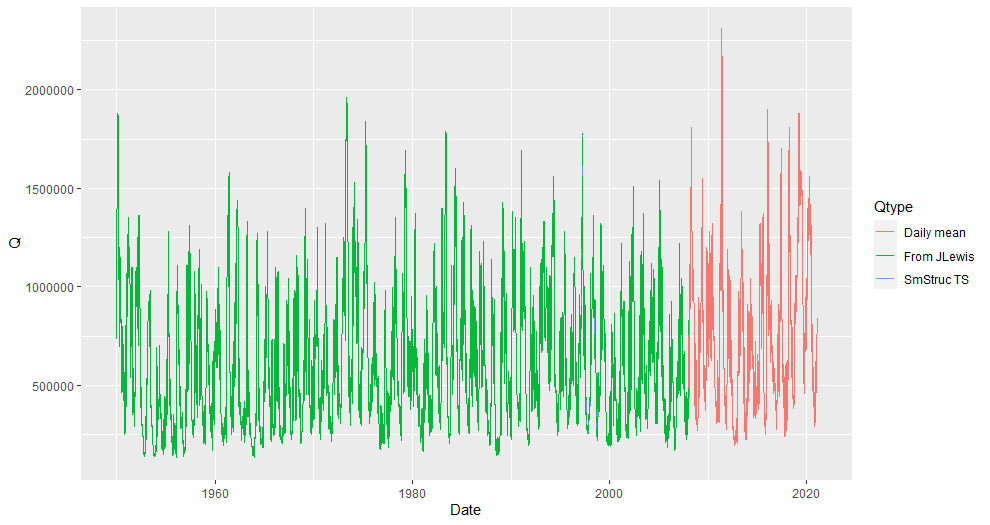


Figure SI3-6. Daily streamflow (Q) in cubic feet per second for MS-atVIC where the green line is streamflow provided by U.S. Army Corps of Engineers written communications (from JLewis), the salmon line is daily mean streamflow from the U.S. Geological Survey (daily mean), and the blue line is streamflow estimated using a smoothed structural time series model (SmStruc TS). Data from Murphy, J. C., Mize, S. V., Swarzenski, C. M., and Schafer, L. A., 2022, Datasets of suspended sediment concentration and percent fines (1973–2021), sampling information (1973–2021), and daily streamflow (1928–2021) for sites in the Lower Mississippi and Atchafalaya Rivers to support analyses of sediment transport and delivery: U.S. Geological Survey data release, <https://doi.org/10.5066/P9YK3S9R>.

# Atchafalaya River @ Wax Lake Outlet (AT-WAXL | AT-WAXLqx)

The retrieved daily streamflow data for Wax Lake Outlet contains many multi-day gaps and no daily values are available beyond water year (WY) 2015; thus, a complete daily flow record was stitched together using a variety of methods:

1. Observed daily streamflows were used whenever possible (salmon line in plot below)
2. Recent daily values (WY 2016 to end of record) were calculated from the unit values (15-minute observations) record (green line below).
3. Remaining missing days were estimated using an interpolated daily streamflow ratio between AT-WAXL and the Atchafalaya River at Simmesport (AT-SIMM) (blue line below). Daily ratios were calculated for every day that had measured streamflow at both locations. Linear interpolation was used to estimate a daily ratio for all other days. This daily ratio was then multiplied by the streamflow at AT-SIMM to give a daily estimate of streamflow at AT-WAXL.


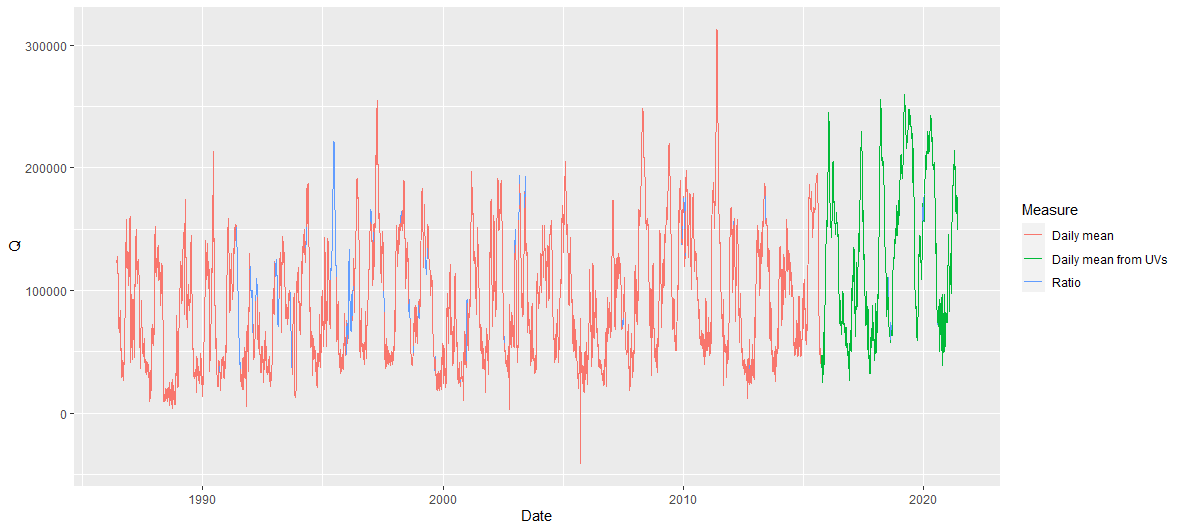


Figure SI3-7. Daily streamflow (Q) in cubic feet per second at Wax Lake Outlet where the salmon line is daily mean streamflow from the U.S. Geological Survey (USGS), the green line is computed daily mean streamflow from unit values (UVs; also from USGS), and the blue line is estimated daily streamflow using an interpolated daily streamflow ratio. Data from Murphy, J. C., Mize, S. V., Swarzenski, C. M., and Schafer, L. A., 2022, Datasets of suspended sediment concentration and percent fines (1973–2021), sampling information (1973–2021), and daily streamflow (1928–2021) for sites in the Lower Mississippi and Atchafalaya Rivers to support analyses of sediment transport and delivery: U.S. Geological Survey data release, <https://doi.org/10.5066/P9YK3S9R>.

Prior to applying method 3, this approach was first tested at Wax Lake Outlet (AT-WAXL) and Morgan City (AT-MORG). Half the observed values were held out as a test dataset and the remaining half of the observations were used to compute daily streamflow ratios, interpolate daily ratios for missing days, and ultimately estimate daily streamflow for the held-out data. This approach was compared to using subtraction between the Simmesport (AT-SIMM) and the lower two Atchafalaya gages (e.g., daily streamflow estimate at AT-WAXL = AT-SIMM – AT-MORG). The interpolated daily ratio method gave estimates with higher precision and accuracy than the subtraction method, especially for high streamflows. Additionally, the interpolated daily ratio method does not lead to abrupt up or downward shifts in the timeseries, which is an artifact of the subtraction method.

Mean differences across test (held-out) dataset:

- Subtraction approach = 2,341 cubic feet per second (cfs)
- Ratio approach = AT-WAXL: 123 cfs, AT-MORG: 105 cfs

Figure SI3-8. Error (Estimated – Observed), in cubic feet per second using the interpolated daily ratio method (left panel) and subtraction method (right panel) for Morgan City (MCY) and Wax Lake Outlet (WLO). For the boxplots, the bottom, middle, and top of the box of represent the 25th percentile, median value, 75th percentile, respectively. Top and bottom whiskers extend to the largest or smallest value or no farther than +/- 1.5*IQR (interquartile range). Solid black circles are outliers beyond +/- 1.5*IQR.


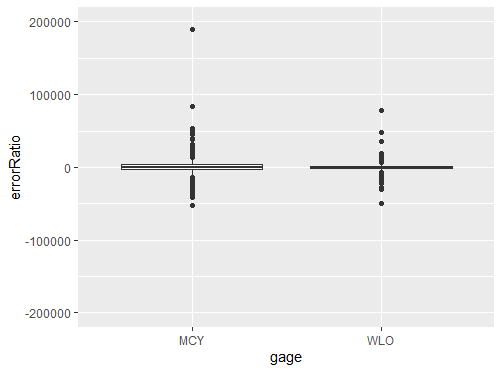

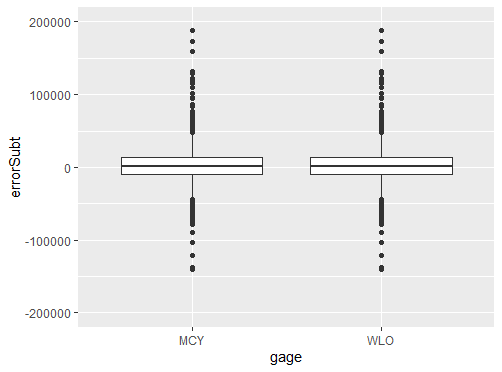


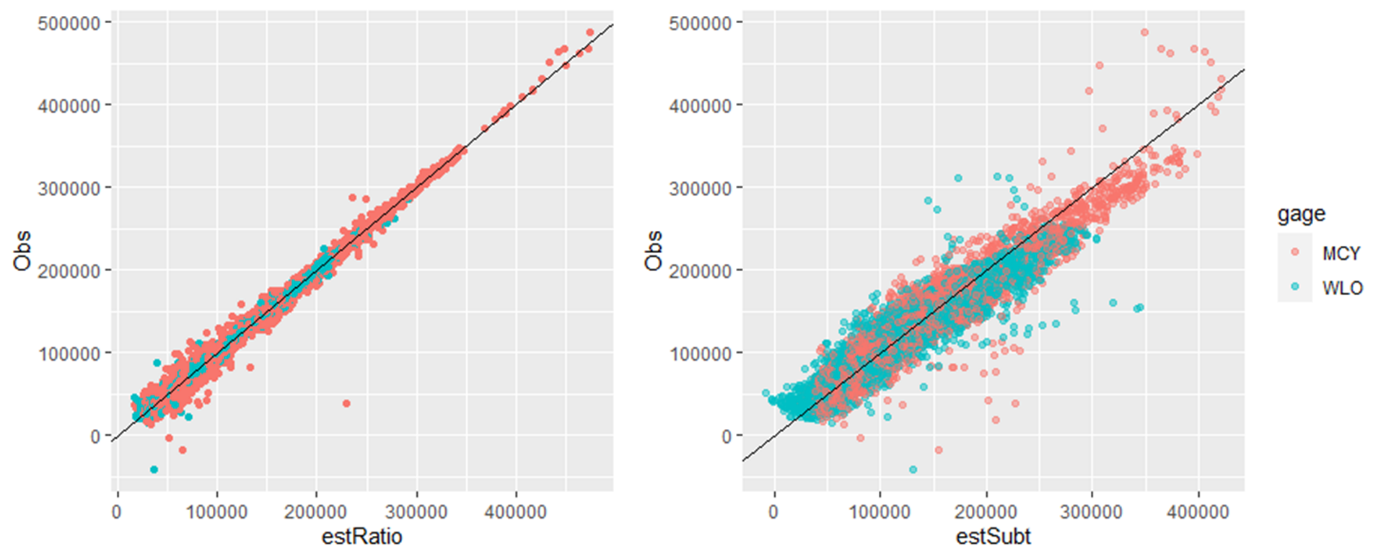


Figure SI3-9. Plot of observed (Obs) versus estimated daily streamflow values with a 1:1 line for interpolated daily ratio method (left panel, estRatio) and subtraction method (right panel, estSubt), where salmon points are Morgan City (MCY) and blue dots are Wax Lake Outlet (WLO).

# Atchafalaya River @ Morgan City (AT-MORG | AT-MORGqx)

The retrieved daily USGS streamflow data for the Atchafalaya River at Morgan City gage contain many multi-day gaps, no daily values after water year 2015, and no measurements prior to September 30, 1995. Like the Atchafalaya River at Wax Lake Outlet, a complete daily flow record was stitched together using a variety of methods:

1. Observed daily streamflows were used whenever possible (salmon line in plot below)
2. Recent daily values (WY 2016 to end of record) were calculated from the unit values (15-minute observations) record (green line below).
3. Like at Wax Lake Outlet, the remaining gaps were estimated using an interpolated daily streamflow ratio between AT-MORG and AT-SIMM (blue line below). (See description above).
4. Because no data are available at AT-MORG prior to WY 1995, the following pre-defined daily streamflow ratios were inserted into the record and interpolation was used to estimate the remaining daily ratios:
   1. 1989-10-01 to 1992-09-30: 0.30
   2. 1992-10-02 to 1993-09-30: interpolated
   3. 1993-10-01 to 1995-07-31: 0.416
   4. 1995-08-01 to start of observed record: interpolated


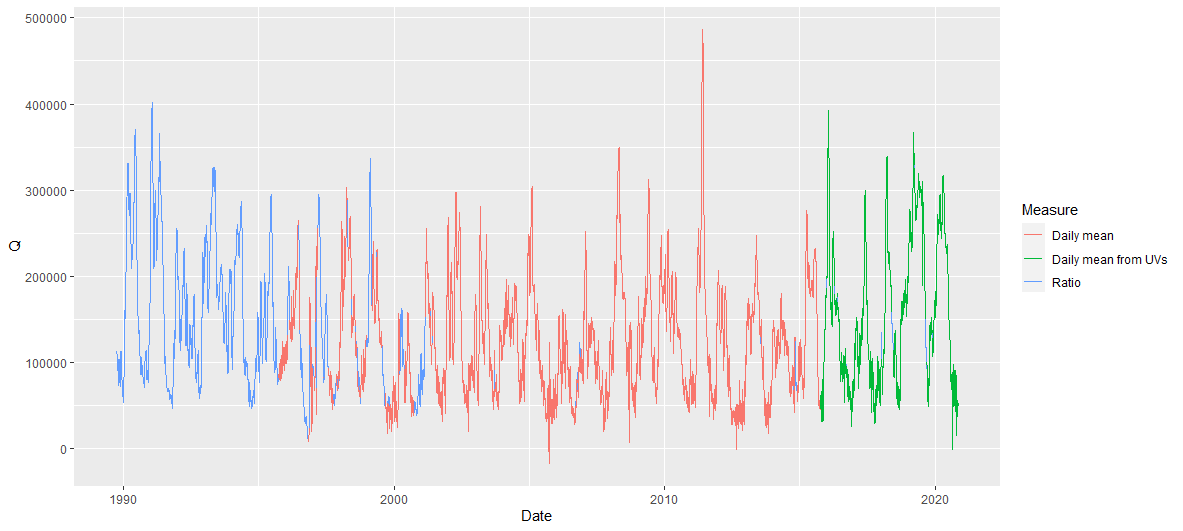
These inserted and interpolated daily ratios were then used with the AT-SIMM streamflow record to estimate daily streamflow at AT-MORG. These estimates lack measures of uncertainty and their accuracy is not known.

Figure SI3-10. Daily streamflow (Q) in cubic feet per second at Morgan City where the salmon line is daily mean streamflow from the U.S. Geological Survey, the green line is calculated daily mean streamflow from unit values (UVs), and the blue line is estimated daily streamflow using an interpolated daily streamflow ratio. Data from Murphy, J. C., Mize, S. V., Swarzenski, C. M., and Schafer, L. A., 2022, Datasets of suspended sediment concentration and percent fines (1973–2021), sampling information (1973–2021), and daily streamflow (1928–2021) for sites in the Lower Mississippi and Atchafalaya Rivers to support analyses of sediment transport and delivery: U.S. Geological Survey data release, <https://doi.org/10.5066/P9YK3S9R>.

# Mississippi River @ Belle Chasse (MS-BELL | MS-BELLqx)

Streamflow at the Mississippi River at Belle Chasse gage begins on 2008-10-29, but the earliest suspended sediment sample at this site is approximately 1.5 years earlier on 2006-06-02. To extend the streamflow record at Belle Chasse back to the beginning of water year (WY) 2006 (2005-10-01) a similar “interpolated daily streamflow ratio” method (used for filling long gaps at AT-WAXL and AT-MORG) was used at MS-BELL as well. At MS-BELL, streamflow data were paired with the upstream Mississippi River at Baton Rouge (MS-BATO) gage. Data prior to the spring of 2017 (2017-03-15) were used to compute three streamflow ratios (MS-BELL / MS-BATO) based on three ranges of flow (i.e., flow classes):

Table SI3-2. Table of flow classifications and their corresponding daily streamflow range and mean daily streamflow ratio.

| **Flow class** | **Daily streamflow range, in cubic feet per second** | **Mean daily streamflow ratio** |
| --- | --- | --- |
| High | >= 1,000,000 | 0.926 |
| Medium | >= 200,000 & < 1,000,000 | 0.997 |
| Low | < 200,000 | 1.16 |


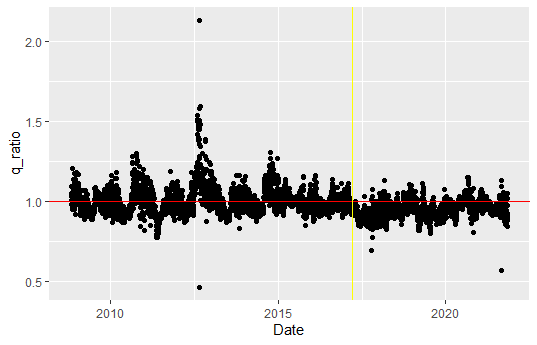
Figure SI3-11. Daily observed streamflow ratios for Bell Chasse and Baton Rouge. The yellow line indicates a step change. Red line is at y=1, indicating same flow magnitudes at Belle Chasse and MS-BATO.

The plot above (Figure SI3-11) of the observed daily streamflow ratios (Belle Chasse / Baton Rouge) over time shows a step change in the spring of 2017 where the daily streamflow ratio shifts to lower values (yellow line). Because of this shift, only data prior to 2017-03-15 were used to compute the average daily streamflow ratios (table above).


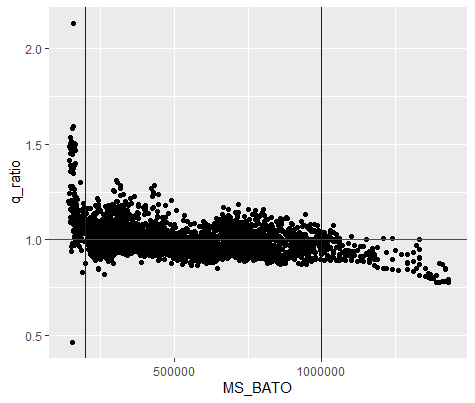
Plotting the observed daily streamflow ratios (prior to 2017-03-15) against daily streamflow at Baton Rouge (in cfs) shows a pattern (Figure SI3-12). The lowest streamflows (<200,000 cfs) tend to have higher daily ratios whereas streamflows at MS-BATO that exceed a million cubic feet per second tend to have daily ratios less than 1. Because of these patterns, average daily streamflow ratios were computed for these three flow ranges (delineated by the blue lines) and shown in Table SI3-2.

Figure SI3-12. Daily streamflow ratio between Belle Chasse and Baton Rouge (MS-BELL/MS-BATO) versus the observed streamflow at Baton Rouge (MS-BATO) on the same day. Blue vertical lines divide streamflow at MS-BATO into three flow classes. Red line is at y=1, indicating same flow magnitudes at MS-BELL and MS-BATO.

From the start of WY 2006 through the beginning of the observed streamflow record (2008-10-29), an average daily Q ratio (see Table SI3-2) was inserted into the record at MS-BELL on every Tuesday and Saturday, based on the streamflow magnitude at MS-BATO on that day. The remaining daily ratios were estimated using linear interpolation, and a complete daily record of streamflow over this whole period was estimated by multiplying the daily streamflow ratio and the observed daily streamflow at MS-BATO.


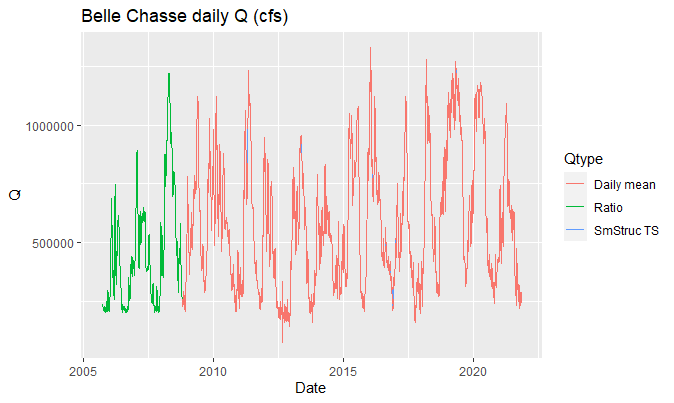


Figure SI3-13. Daily streamflow (Q) in cubic feet per second at Mississippi River at Belle Chasse where the salmon line is the observed daily mean streamflow from the U.S. Geological Survey (Daily mean), the green line is the estimated streamflow using interpolated daily streamflow ratio method (Ratio), and the blue line is the estimated smoothed streamflow from a structural time series model (SmStruc TS).
