# Supplementary material for: Differing field methods and site conditions lead to varying bias in suspended sediment concentrations in the Lower Mississippi and Atchafalaya Rivers
Source: Environ Monit Assess. 2023 Oct 2;195(11):1260. doi: 10.1007/s10661-023-11836-z (PMC10749891; doi:10.1007/s10661-023-11836-z)
Supplement: Supplementary file 4 — Supplementary file4 (PDF 3210 KB) [file 10661_2023_11836_MOESM4_ESM.pdf]

Differing field methods and site conditions lead to varying bias in suspended sediment concentrations in the Lower Mississippi and Atchafalaya Rivers

Environmental Monitoring and Assessment

Online Resource 4 – Site-specific timelines of all field method information [U.S. Geological Survey National Water Information System (NWIS) Database]

J. Murphy[1]
L. Schafer[2]
S. Mize[3]

[1]U.S. Geological Survey, DeKalb, Illinois, USA; jmurphy@usgs.gov
[2]U.S. Geological Survey, Catonsville, Maryland, USA
[3]U.S. Geological Survey, Baton Rouge, Louisiana, USA

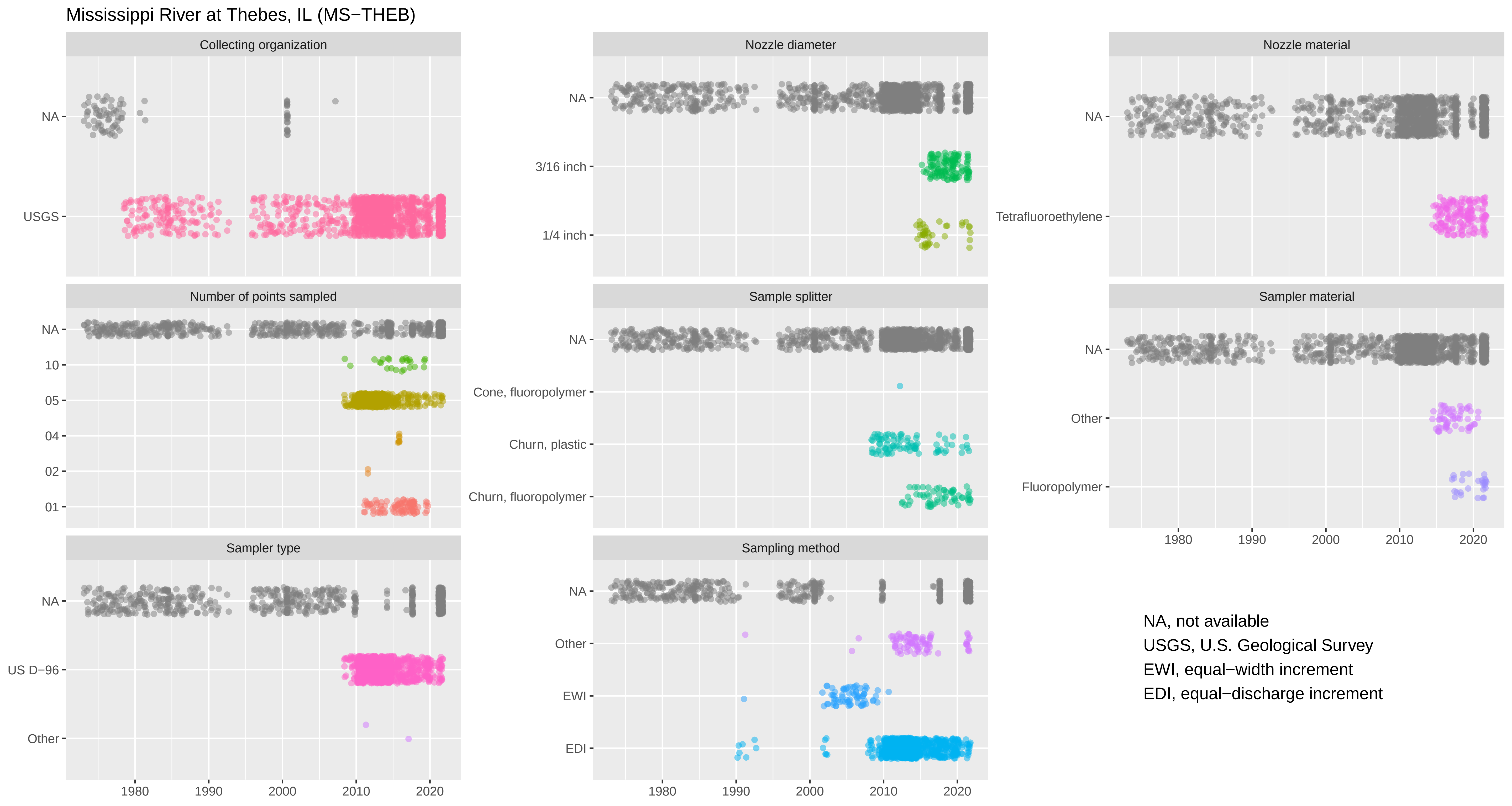
Mississippi River at Thebes, IL (MS−THEB)
Collecting organization
NA
USGS
Nozzle diameter
NA
3/16 inch
1/4 inch
Nozzle material
NA
Tetrafluoroethylene
Number of points sampled
NA
10
05
04
02
01
Sample splitter
NA
Cone, fluoropolymer
Churn, plastic
Churn, fluoropolymer
Sampler material
NA
Other
Fluoropolymer
Sampler type
NA
US D−96
Other
Sampling method
NA
Other
EWI
EDI
1980
1990
2000
2010
2020
NA, not available
USGS, U.S. Geological Survey
EWI, equal−width increment
EDI, equal−discharge increment

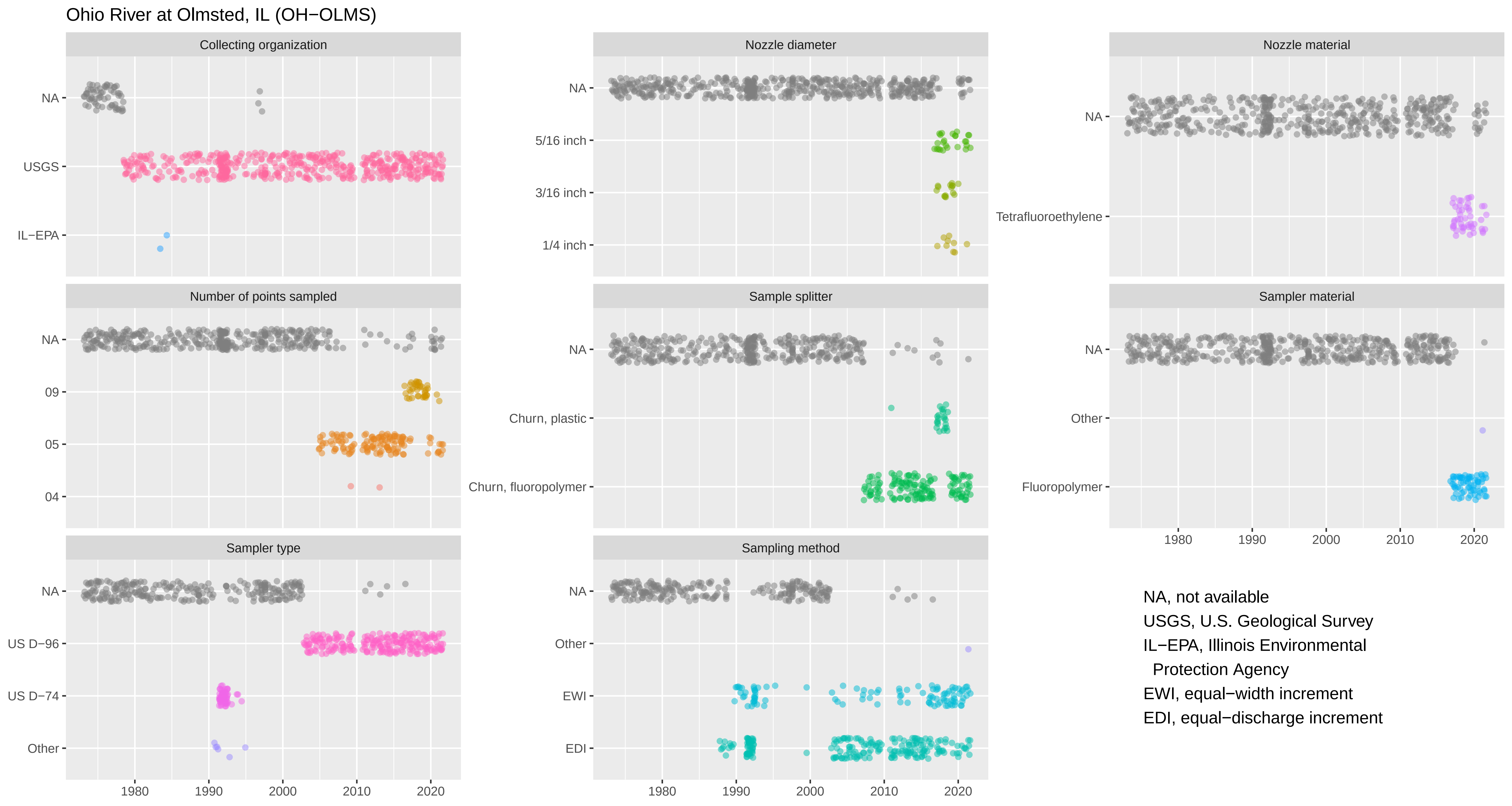

Ohio River at Olmsted, IL (OH−OLMS)
Collecting organization
NA
USGS
IL−EPA
Nozzle diameter
5/16 inch
3/16 inch
1/4 inch
Nozzle material
Tetrafluoroethylene
Number of points sampled
09
05
04
Sample splitter
Churn, plastic
Churn, fluoropolymer
Sampler material
Other
Fluoropolymer
Sampler type
US D−96
US D−74
Other
Sampling method
EWI
EDI
1980
1990
2000
2010
2020
NA, not available
USGS, U.S. Geological Survey
IL−EPA, Illinois Environmental Protection Agency
EWI, equal−width increment
EDI, equal−discharge increment

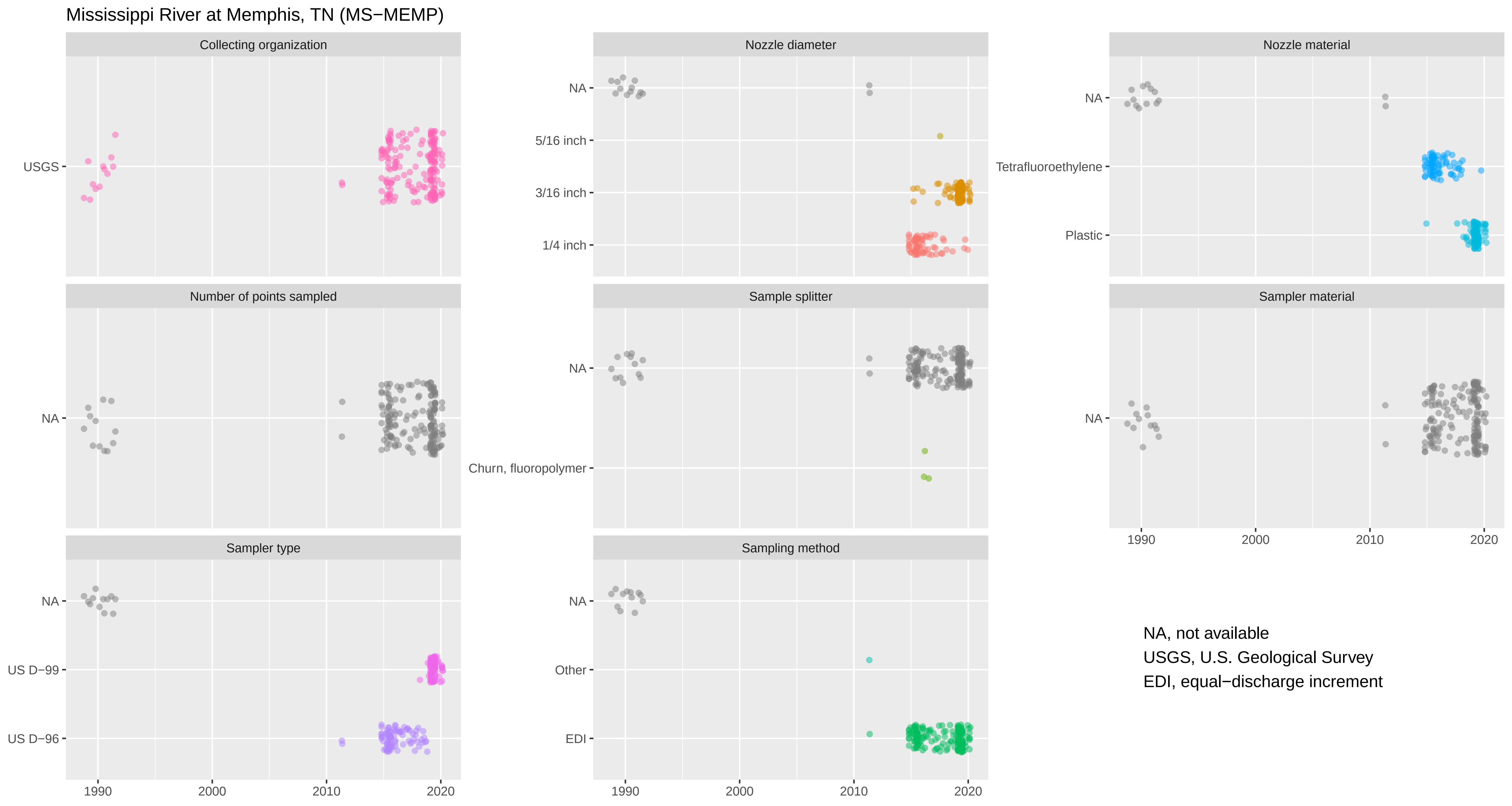
Mississippi River at Memphis, TN (MS−MEMP)
Collecting organization
USGS
Nozzle diameter
NA
5/16 inch
3/16 inch
1/4 inch
Nozzle material
NA
Tetrafluoroethylene
Plastic
Number of points sampled
NA
Sample splitter
NA
Churn, fluoropolymer
Sampler material
NA
Sampler type
NA
US D−99
US D−96
Sampling method
NA
Other
EDI
1990
2000
2010
2020
NA, not available
USGS, U.S. Geological Survey
EDI, equal−discharge increment

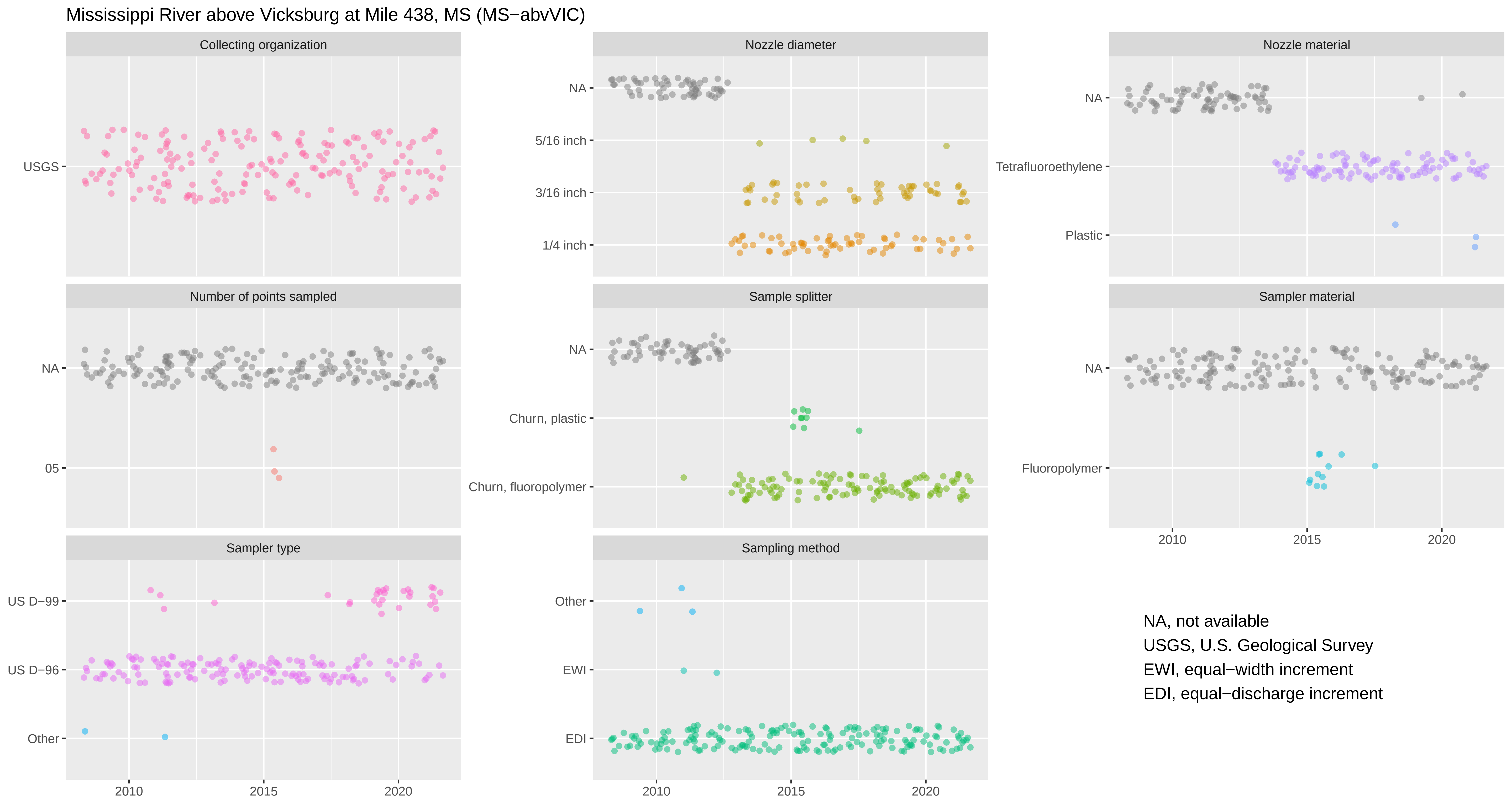
Mississippi River above Vicksburg at Mile 438, MS (MS−abvVIC)
Collecting organization
USGS
Nozzle diameter
NA
5/16 inch
3/16 inch
1/4 inch
Nozzle material
NA
Tetrafluoroethylene
Plastic
Number of points sampled
NA
05
Sample splitter
NA
Churn, plastic
Churn, fluoropolymer
Sampler material
NA
Fluoropolymer
Sampler type
US D−99
US D−96
Other
Sampling method
Other
EWI
EDI
2010
2015
2020
NA, not available
USGS, U.S. Geological Survey
EWI, equal−width increment
EDI, equal−discharge increment

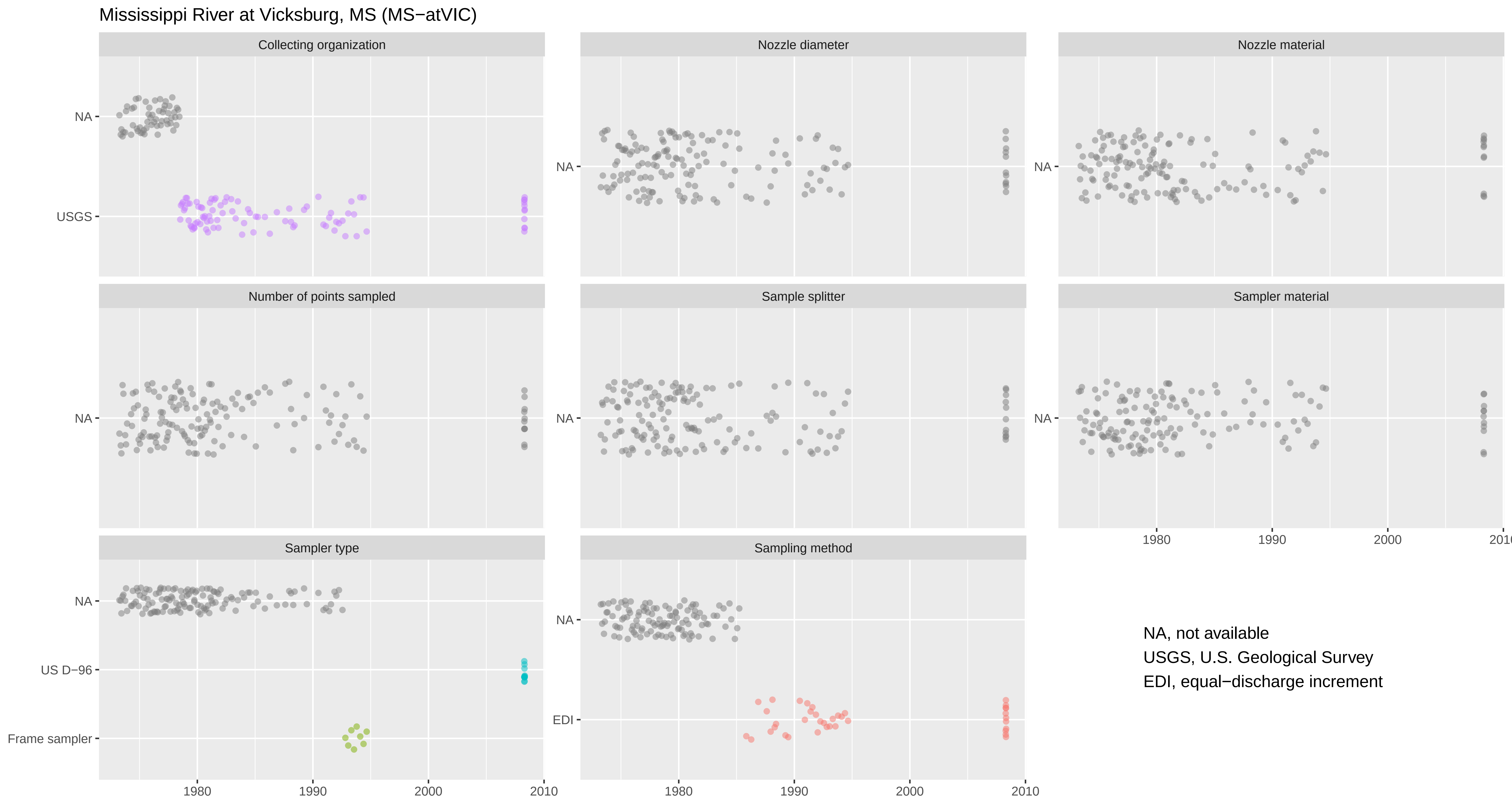

Mississippi River at Vicksburg, MS (MS−atVIC)
Collecting organization
Nozzle diameter
Nozzle material
Number of points sampled
Sample splitter
Sampler material
Sampler type
Sampling method
NA
USGS
US D−96
Frame sampler
EDI
1980
1990
2000
2010
NA, not available
USGS, U.S. Geological Survey
EDI, equal−discharge increment

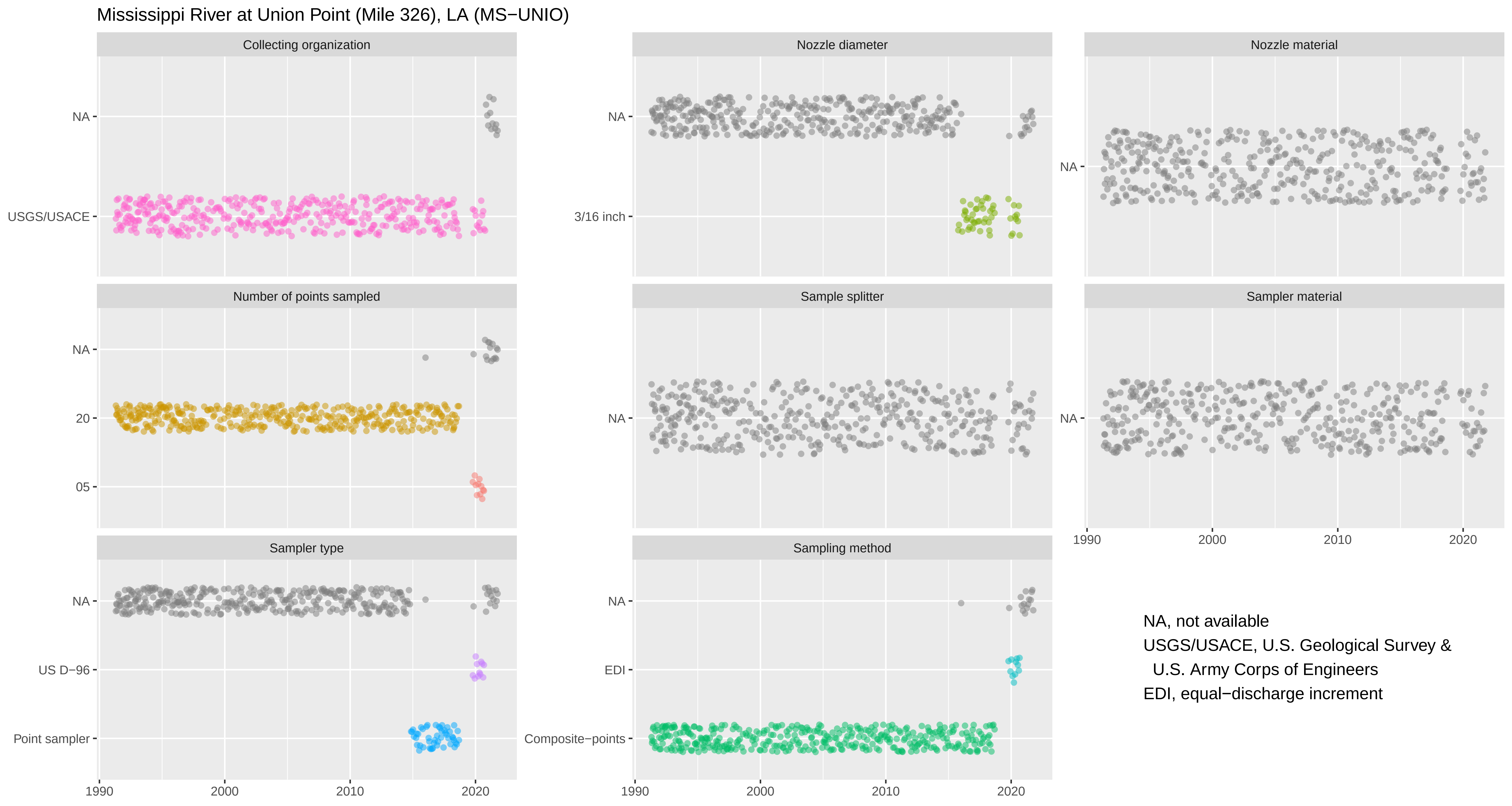
Mississippi River at Union Point (Mile 326), LA (MS−UNIO)
Collecting organization
NA
USGS/USACE
Nozzle diameter
NA
3/16 inch
Nozzle material
NA
Number of points sampled
NA
20
05
Sample splitter
NA
Sampler material
NA
Sampler type
NA
US D−96
Point sampler
Sampling method
NA
EDI
Composite−points
1990
2000
2010
2020
NA, not available
USGS/USACE, U.S. Geological Survey & U.S. Army Corps of Engineers
EDI, equal−discharge increment

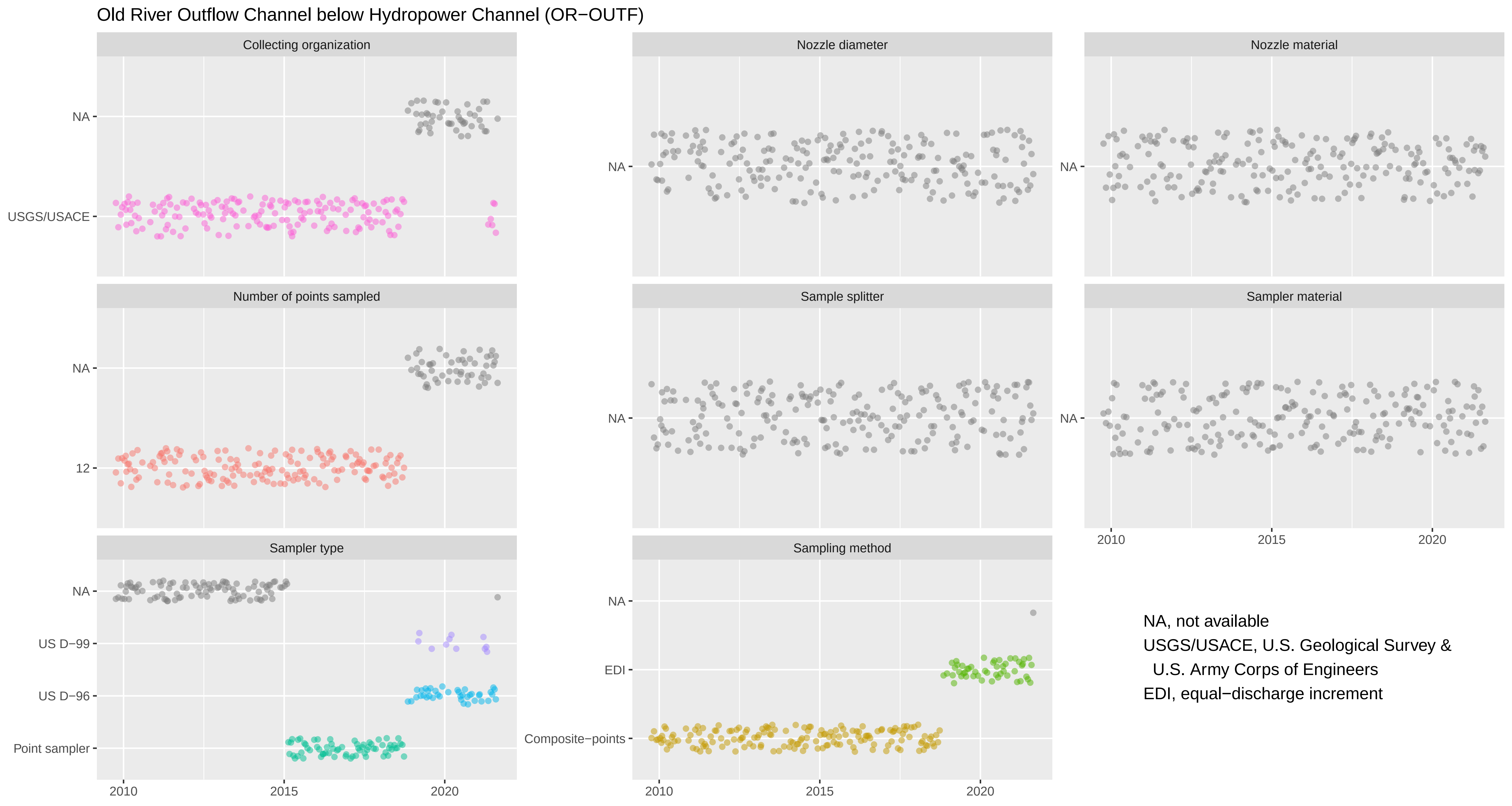
Old River Outflow Channel below Hydropower Channel (OR−OUTF)
Collecting organization
NA
USGS/USACE
Nozzle diameter
NA
Nozzle material
NA
Number of points sampled
NA
12
Sample splitter
NA
Sampler material
NA
Sampler type
NA
US D−99
US D−96
Point sampler
Sampling method
NA
EDI
Composite−points
2010
2015
2020
NA, not available
USGS/USACE, U.S. Geological Survey &
U.S. Army Corps of Engineers
EDI, equal−discharge increment

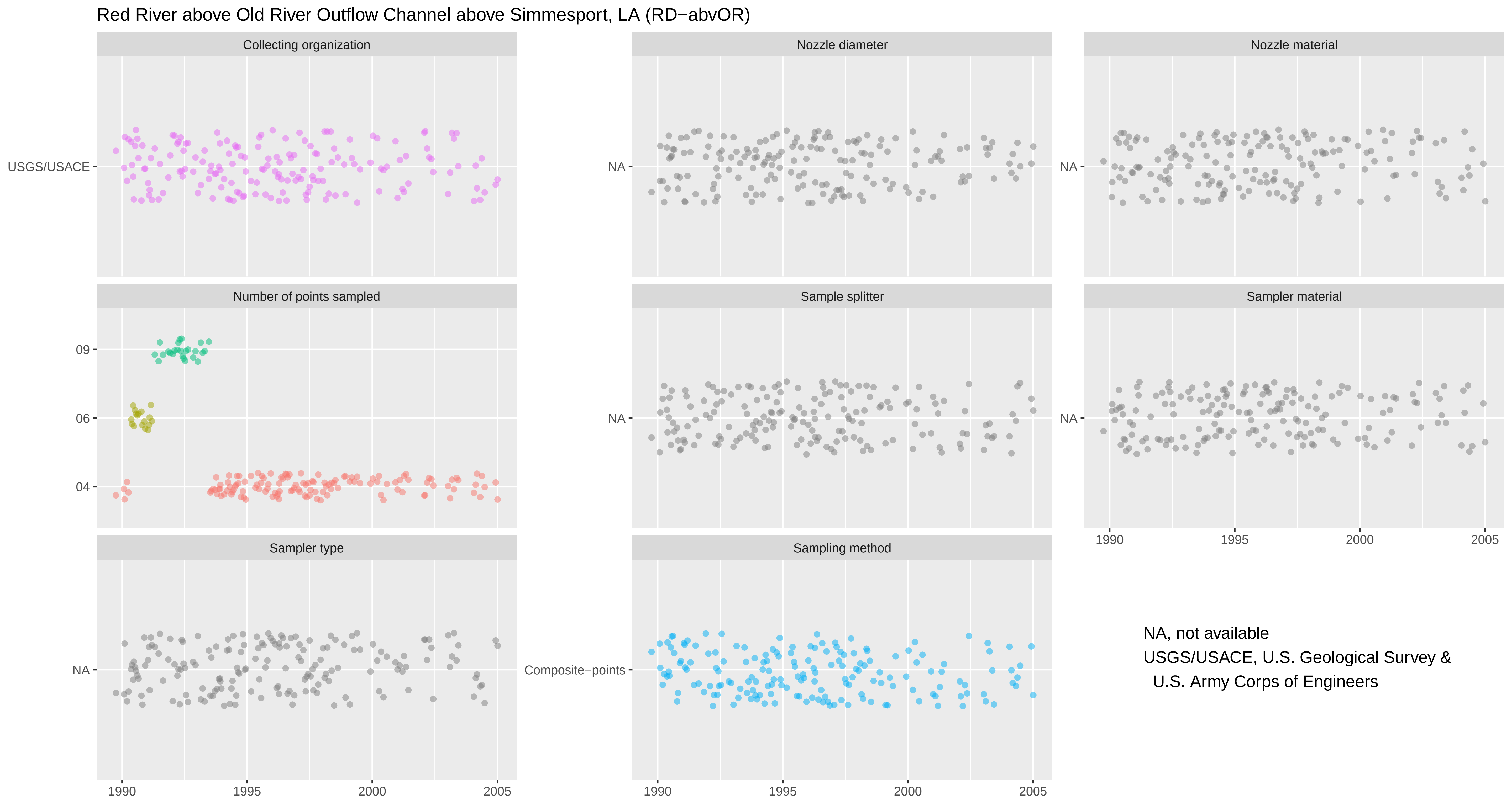

Red River above Old River Outflow Channel above Simmesport, LA (RD−abvOR)
Collecting organization
USGS/USACE
Nozzle diameter
NA
Nozzle material
NA
Number of points sampled
09
06
04
Sample splitter
NA
Sampler material
NA
Sampler type
NA
Sampling method
Composite−points
1990
1995
2000
2005
NA, not available
USGS/USACE, U.S. Geological Survey &
U.S. Army Corps of Engineers

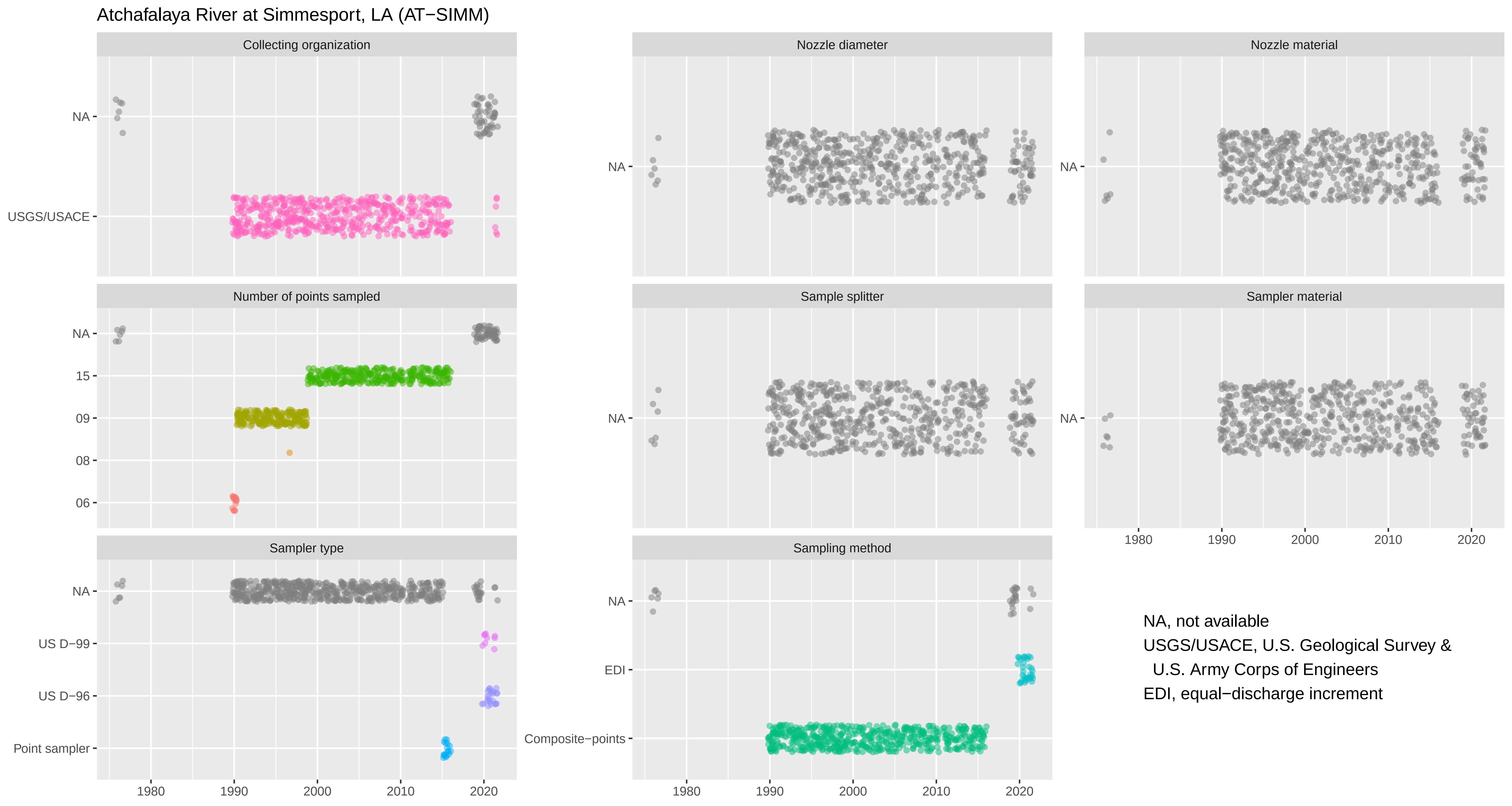
Atchafalaya River at Simmesport, LA (AT−SIMM)
Collecting organization
NA
USGS/USACE
Nozzle diameter
NA
Nozzle material
NA
Number of points sampled
NA
15
09
08
06
Sample splitter
NA
Sampler material
NA
Sampler type
NA
US D−99
US D−96
Point sampler
Sampling method
NA
EDI
Composite−points
1980
1990
2000
2010
2020
NA, not available
USGS/USACE, U.S. Geological Survey &
U.S. Army Corps of Engineers
EDI, equal−discharge increment

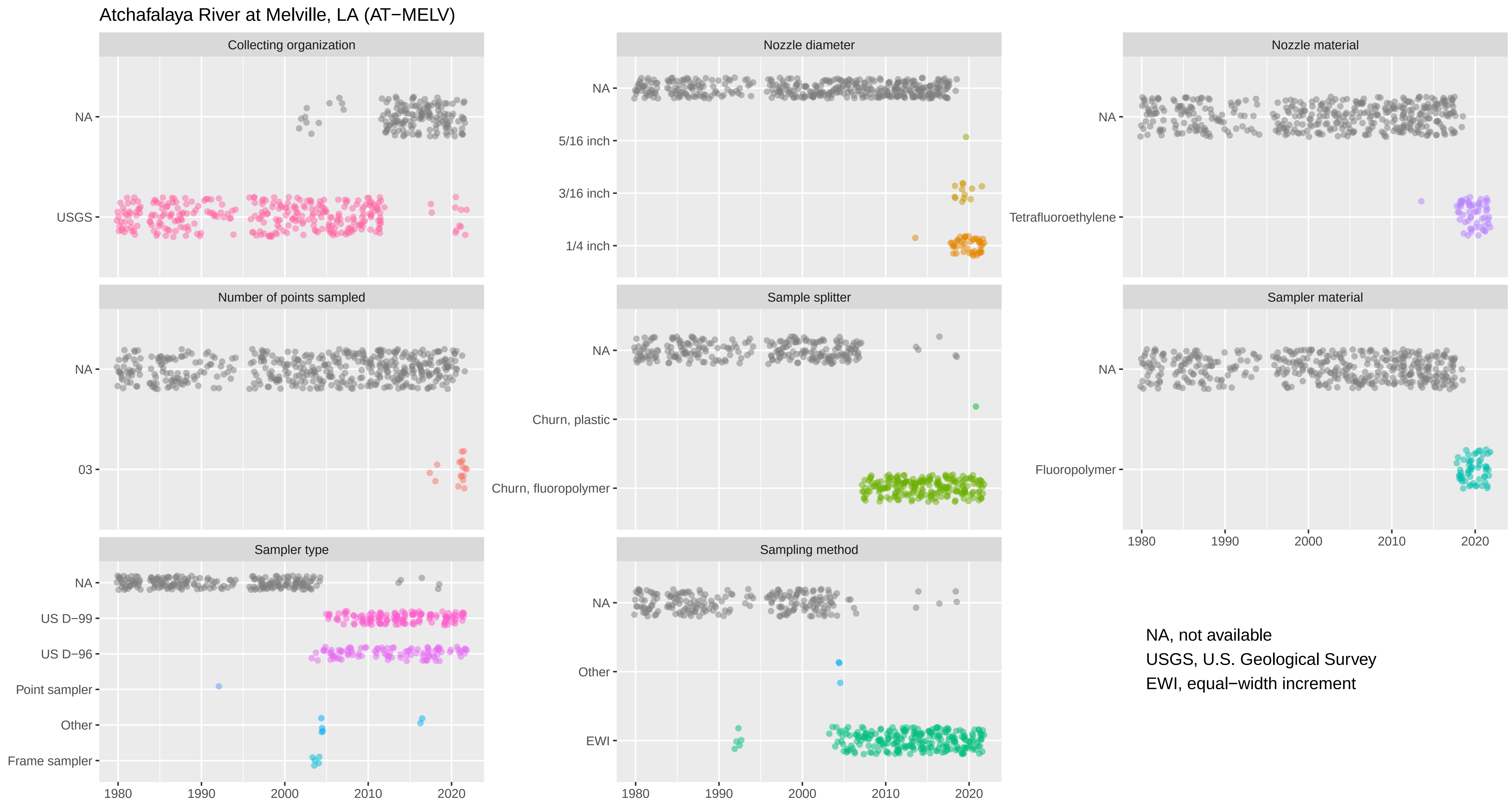

NA, not available
USGS, U.S. Geological Survey
EWI, equal−width increment

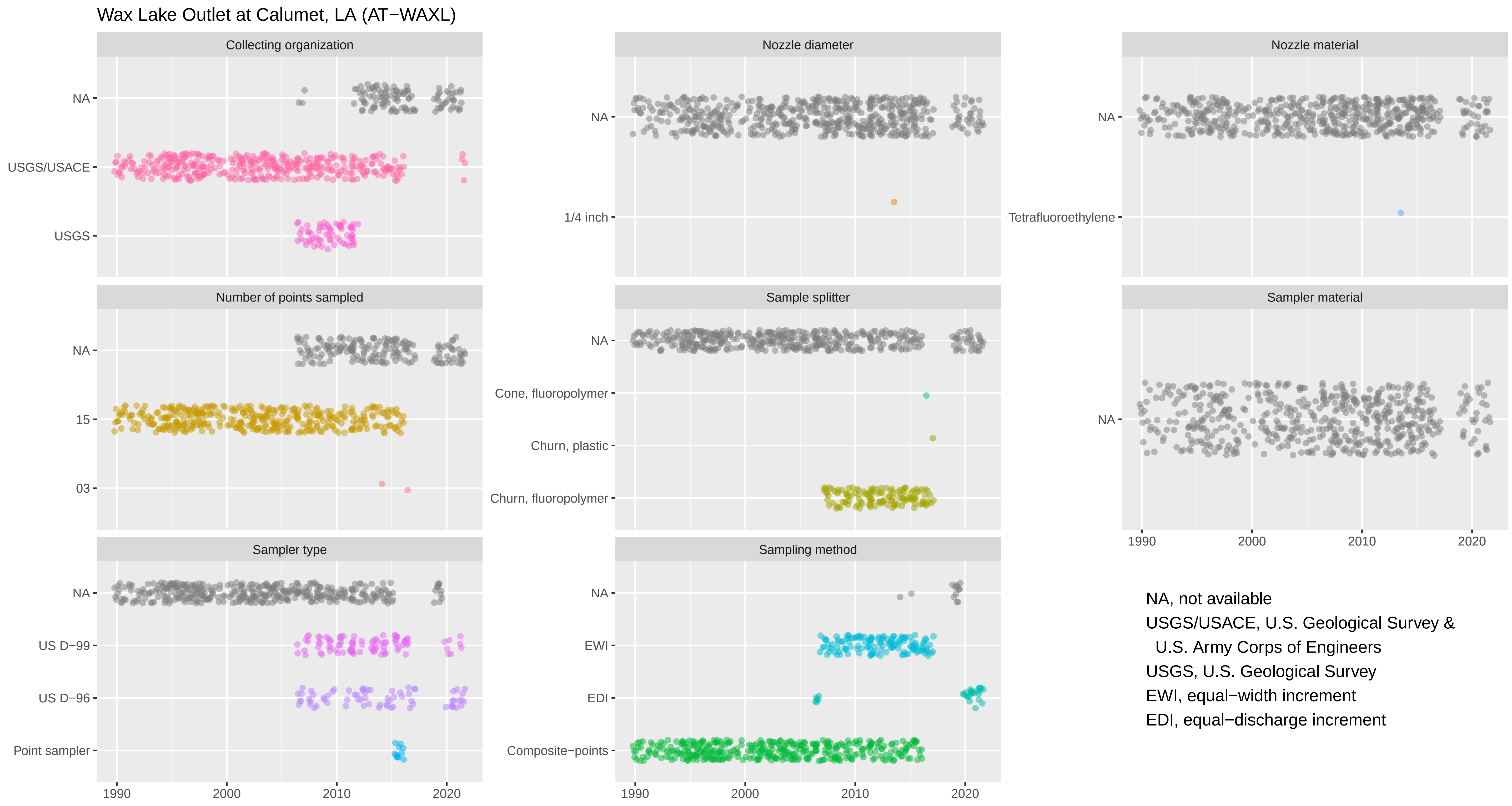

NA, not available
USGS/USACE, U.S. Geological Survey & U.S. Army Corps of Engineers
USGS, U.S. Geological Survey
EWI, equal−width increment
EDI, equal−discharge increment

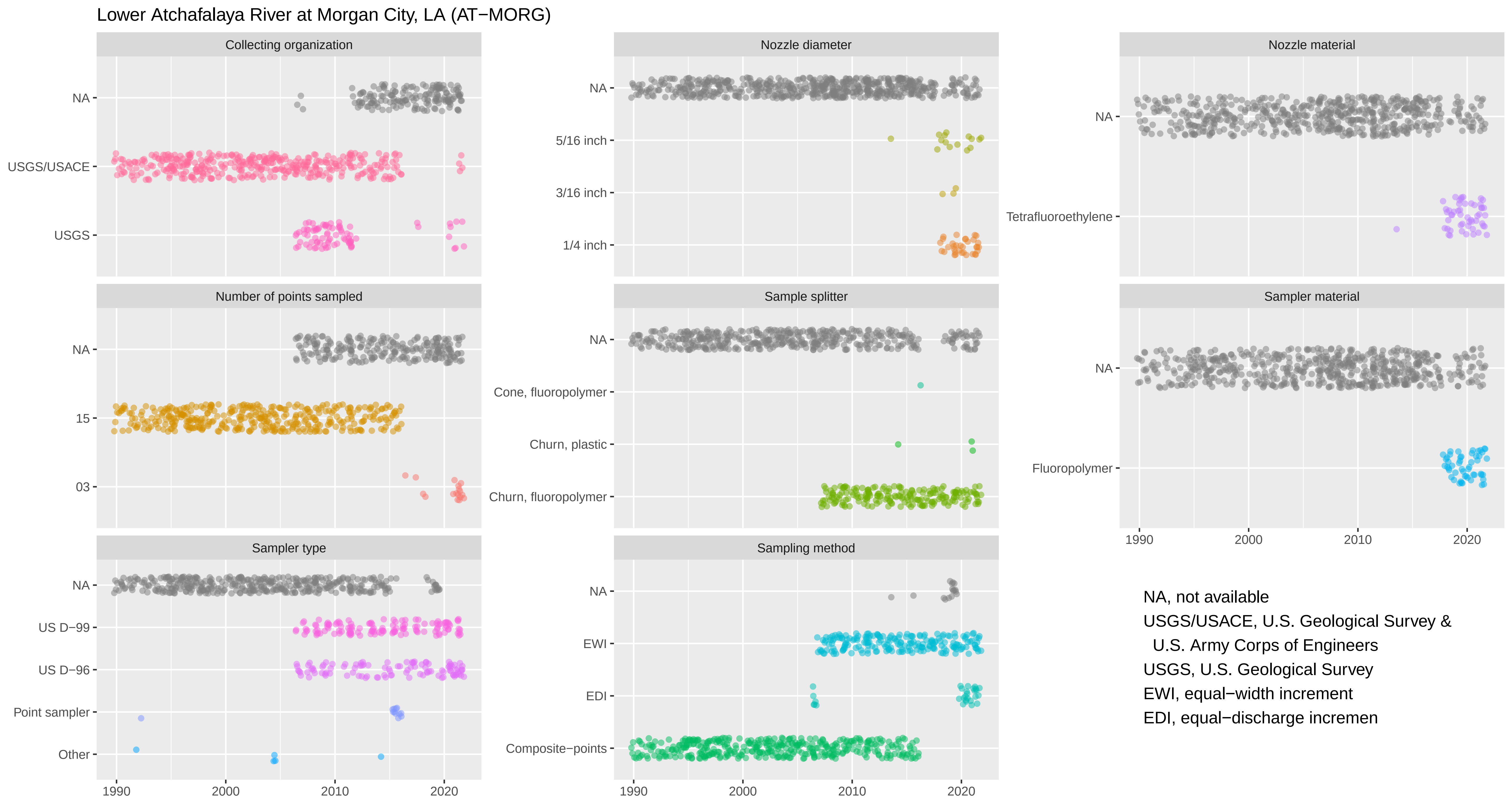

NA, not available
USGS/USACE, U.S. Geological Survey & U.S. Army Corps of Engineers
USGS, U.S. Geological Survey
EWI, equal−width increment
EDI, equal−discharge incremen

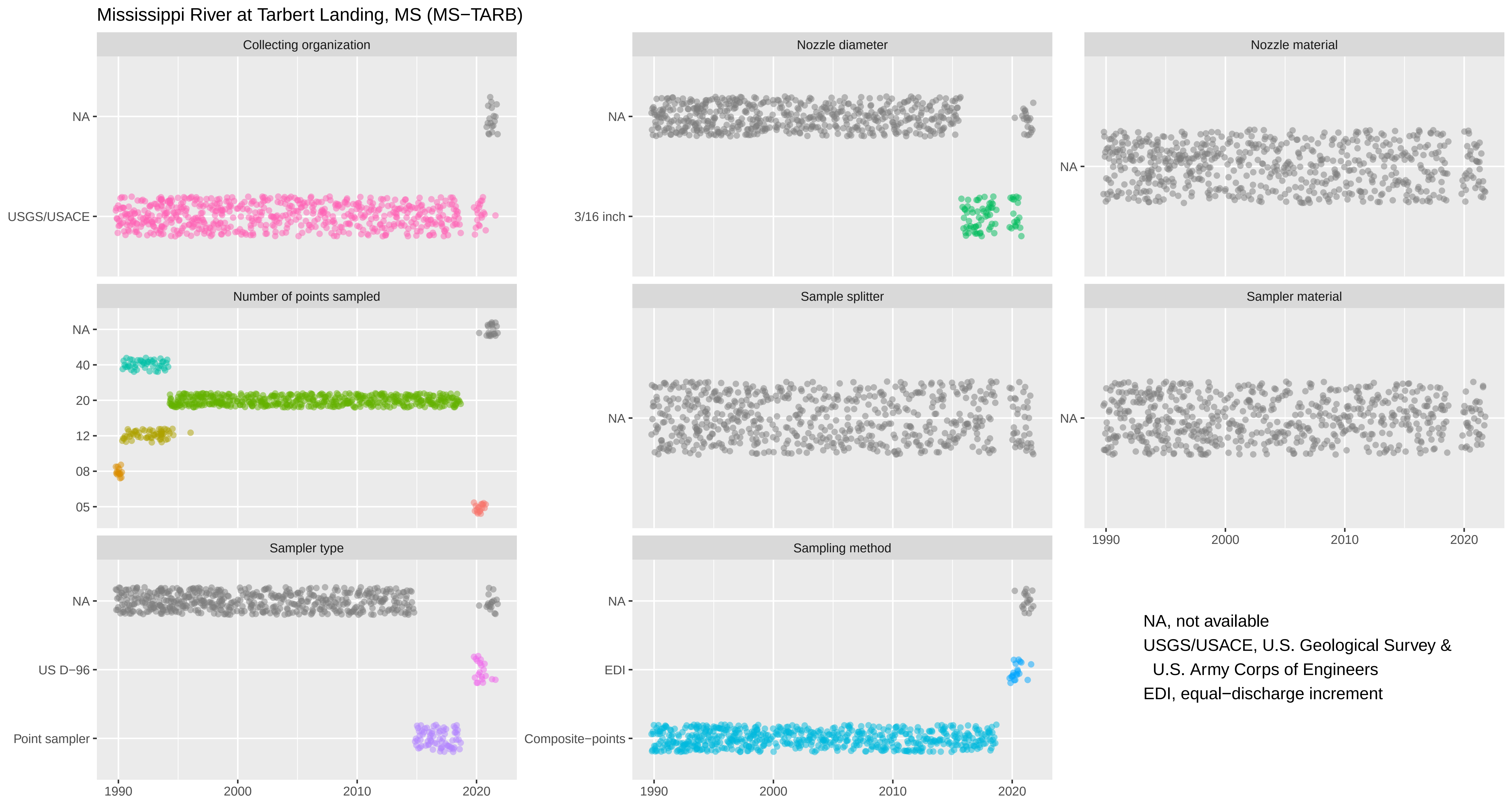

Mississippi River at Tarbert Landing, MS (MS−TARB)
Collecting organization
Nozzle diameter
Nozzle material
Number of points sampled
Sample splitter
Sampler material
Sampler type
Sampling method
NA
USGS/USACE
3/16 inch
40
20
12
08
05
US D−96
Point sampler
EDI
Composite−points
1990
2000
2010
2020
NA, not available
USGS/USACE, U.S. Geological Survey &
U.S. Army Corps of Engineers
EDI, equal−discharge increment

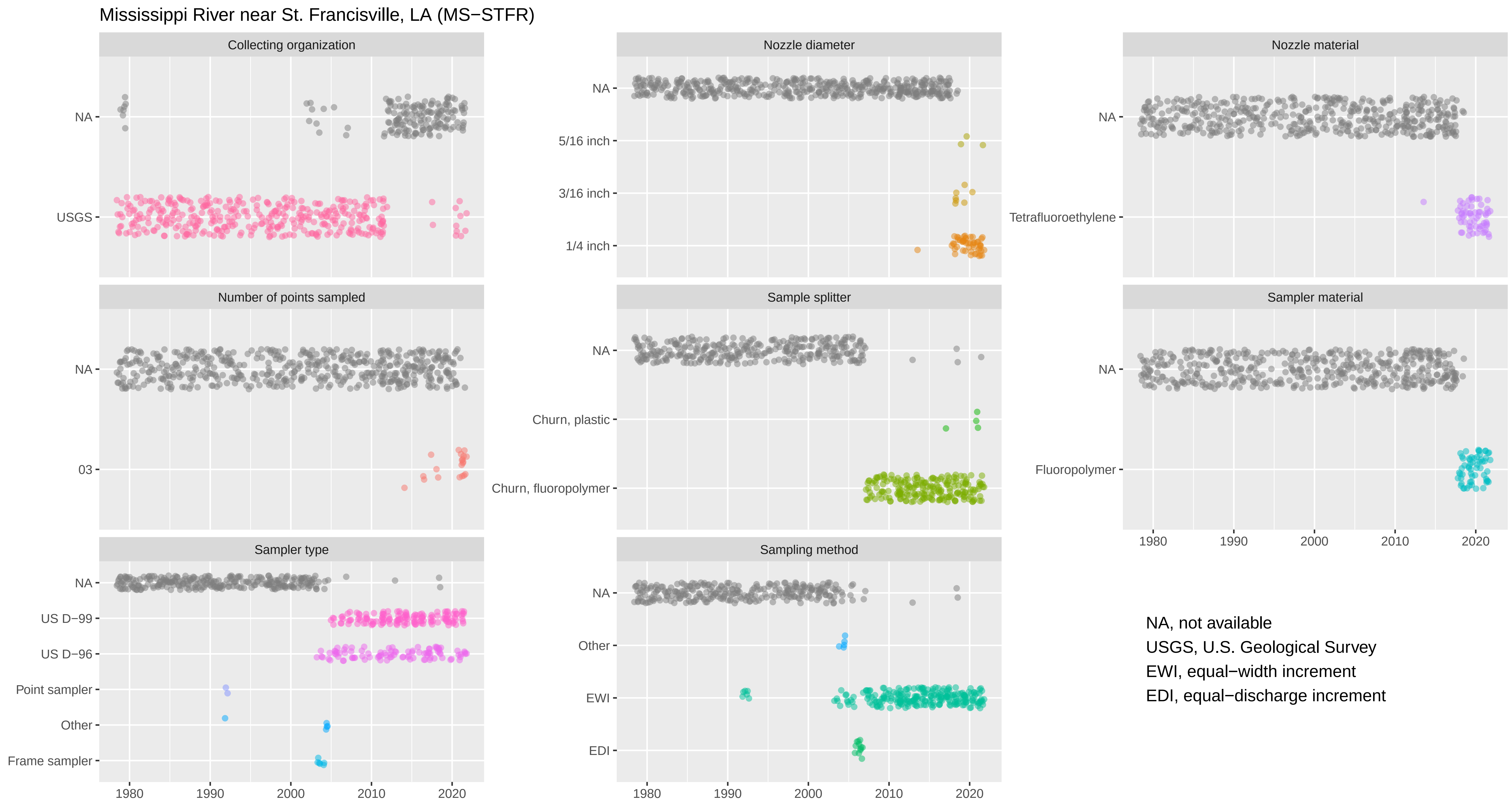
Mississippi River near St. Francisville, LA (MS−STFR)
Collecting organization
NA
USGS
Nozzle diameter
NA
5/16 inch
3/16 inch
1/4 inch
Nozzle material
NA
Tetrafluoroethylene
Number of points sampled
NA
03
Sample splitter
NA
Churn, plastic
Churn, fluoropolymer
Sampler material
NA
Fluoropolymer
Sampler type
NA
US D−99
US D−96
Point sampler
Other
Frame sampler
Sampling method
NA
Other
EWI
EDI
1980
1990
2000
2010
2020
NA, not available
USGS, U.S. Geological Survey
EWI, equal−width increment
EDI, equal−discharge increment

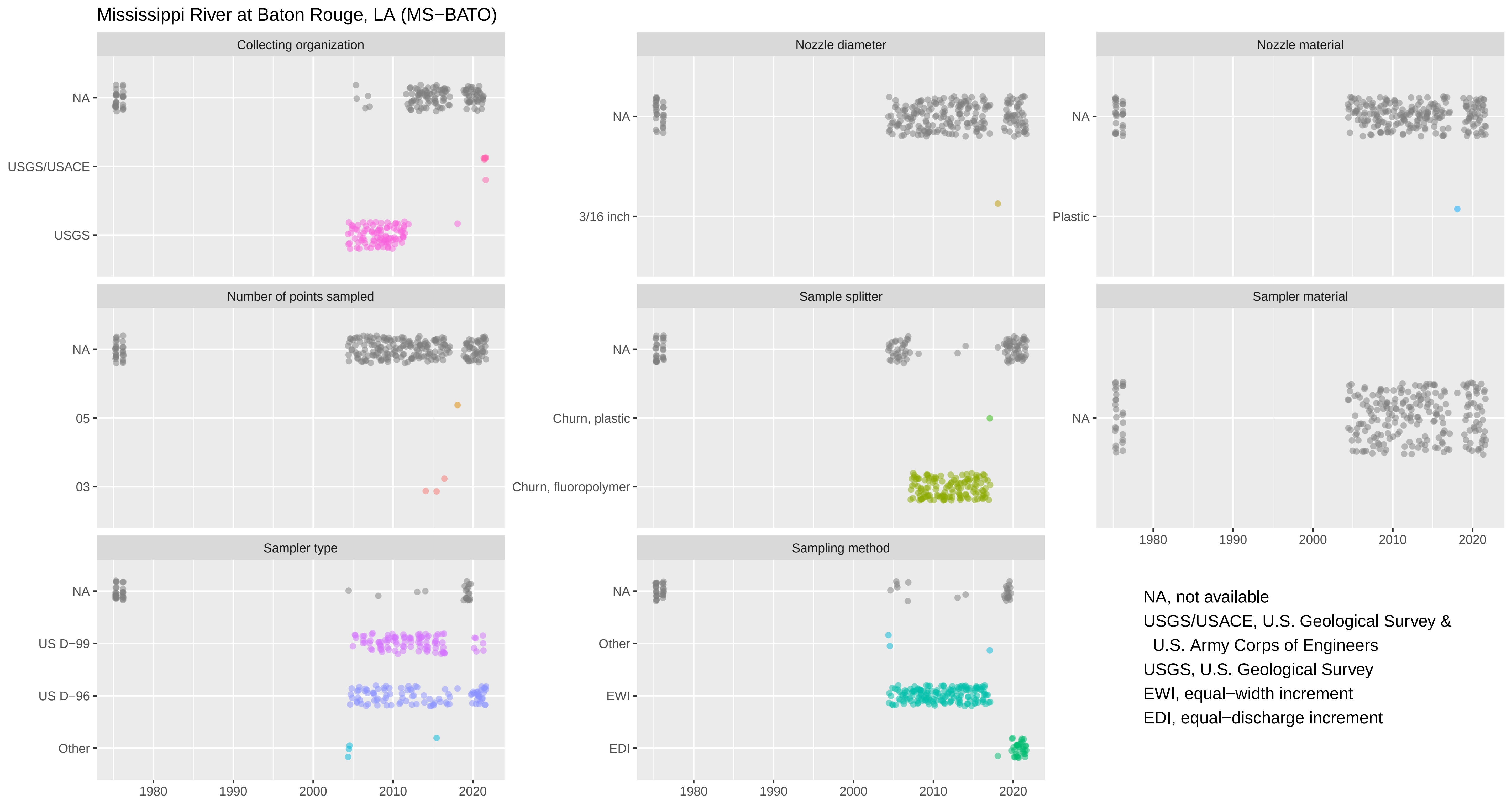
Mississippi River at Baton Rouge, LA (MS−BATO)
Collecting organization
NA
USGS/USACE
USGS
Nozzle diameter
NA
3/16 inch
Nozzle material
NA
Plastic
Number of points sampled
NA
05
03
Sample splitter
NA
Churn, plastic
Churn, fluoropolymer
Sampler material
NA
Sampler type
NA
US D−99
US D−96
Other
Sampling method
NA
Other
EWI
EDI
1980
1990
2000
2010
2020
NA, not available
USGS/USACE, U.S. Geological Survey &
U.S. Army Corps of Engineers
USGS, U.S. Geological Survey
EWI, equal−width increment
EDI, equal−discharge increment

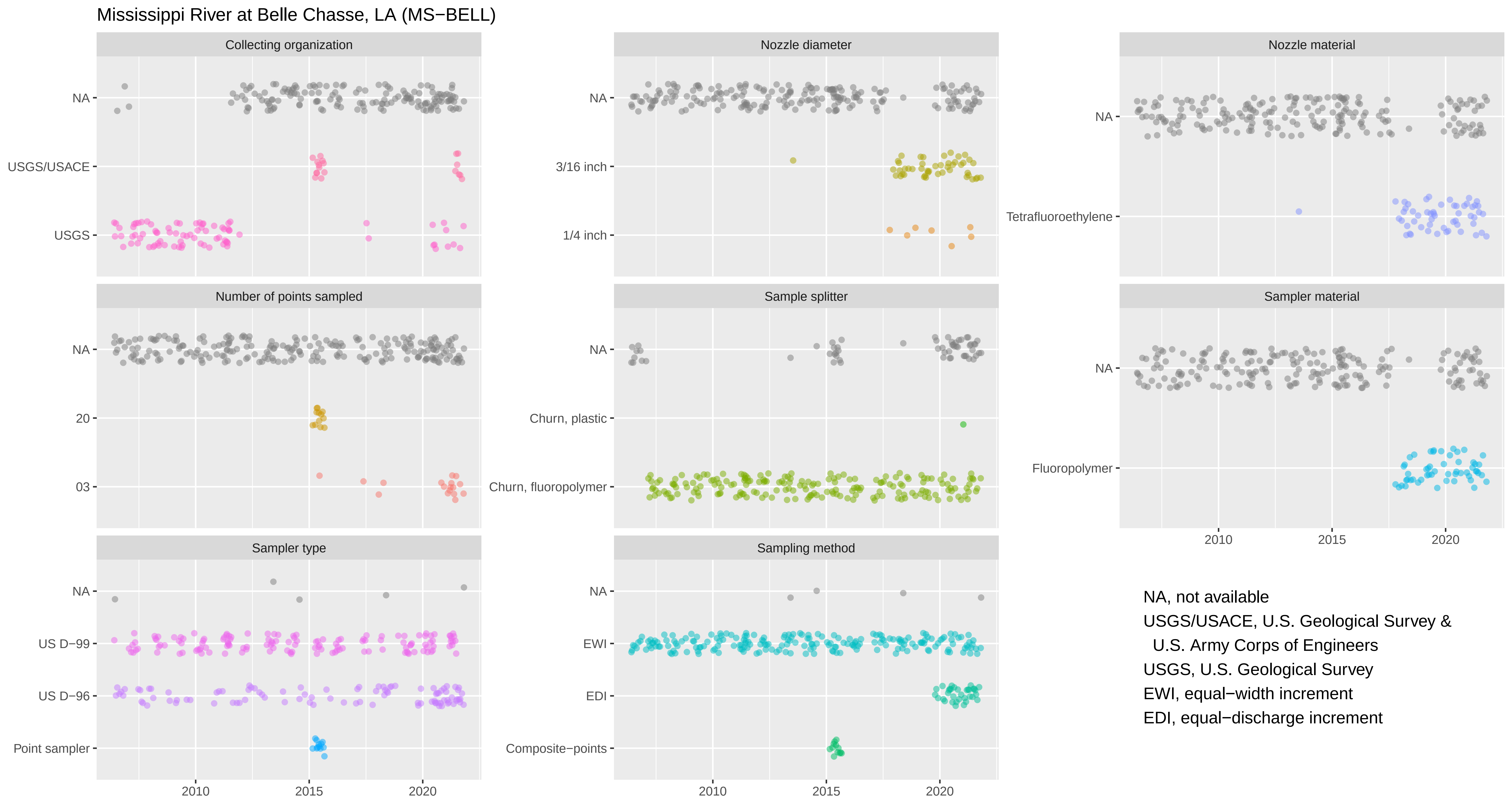

Mississippi River at Belle Chasse, LA (MS−BELL)
Collecting organization
NA
USGS/USACE
USGS
Nozzle diameter
NA
3/16 inch
1/4 inch
Nozzle material
NA
Tetrafluoroethylene
Number of points sampled
NA
20
03
Sample splitter
NA
Churn, plastic
Churn, fluoropolymer
Sampler material
NA
Fluoropolymer
Sampler type
NA
US D−99
US D−96
Point sampler
Sampling method
NA
EWI
EDI
Composite−points
2010
2015
2020
NA, not available
USGS/USACE, U.S. Geological Survey & U.S. Army Corps of Engineers
USGS, U.S. Geological Survey
EWI, equal−width increment
EDI, equal−discharge increment
