# Supplementary material for: Differing field methods and site conditions lead to varying bias in suspended sediment concentrations in the Lower Mississippi and Atchafalaya Rivers
Source: Environ Monit Assess. 2023 Oct 2;195(11):1260. doi: 10.1007/s10661-023-11836-z (PMC10749891; doi:10.1007/s10661-023-11836-z)
Supplement: Supplementary file 5 — Supplementary file5 (PDF 1265 KB) [file 10661_2023_11836_MOESM5_ESM.pdf]

Differing field methods and site conditions lead to varying bias in suspended sediment concentrations in the Lower Mississippi and Atchafalaya Rivers

Environmental Monitoring and Assessment

Online Resource 5 – Field-method-specific timelines of all sites [U.S. Geological Survey National Water Information System (NWIS) Database]

J. Murphy[1]
L. Schafer[2]
S. Mize[3]

[1]U.S. Geological Survey, DeKalb, Illinois, USA; jmurphy@usgs.gov
[2]U.S. Geological Survey, Catonsville, Maryland, USA
[3]U.S. Geological Survey, Baton Rouge, Louisiana, USA

Site abbreviations used in plots below.

**Site (USGS site numbers)**	**Site name**
MS-THEB (07022000)	Mississippi River at Thebes, IL
OH-OLMS (03612600; 03612500)	Ohio River at Olmsted, IL; Ohio River at Dam 53 near Grand Chain, IL
MS-MEMP (07032000)	Mississippi River at Memphis, TN
MS-abvVIC (322023090544500)	Mississippi River above Vicksburg at Mile 438, MS
MS-atVIC (07289000)	Mississippi River at Vicksburg, MS
MS-UNIO (07295025)	Mississippi River at Union Point (Mile 326), LA
MS-TARB (07295100)	Mississippi River at Tarbert Landing, MS
MS-STFR (07373420)	Mississippi River near St. Francisville, LA
MS-BATO (07374000)	Mississippi River at Baton Rouge, LA
MS-BELL (07374525)	Mississippi River at Belle Chasse, LA
RD-abvOR (310408091424500)	Red River above Old River Outflow Channel above Simmesport, LA
OR-OUTF (07381482)	Old River Outflow Channel below Hydropower Channel
AT-SIMM (07381490)	Atchafalaya River at Simmesport, LA
AT-MELV (07381495)	Atchafalaya River at Melville, LA
AT-WAXL (07381590)	Wax Lake Outlet at Calumet, LA
AT-MORG (07381600)	Lower Atchafalaya River at Morgan City, LA

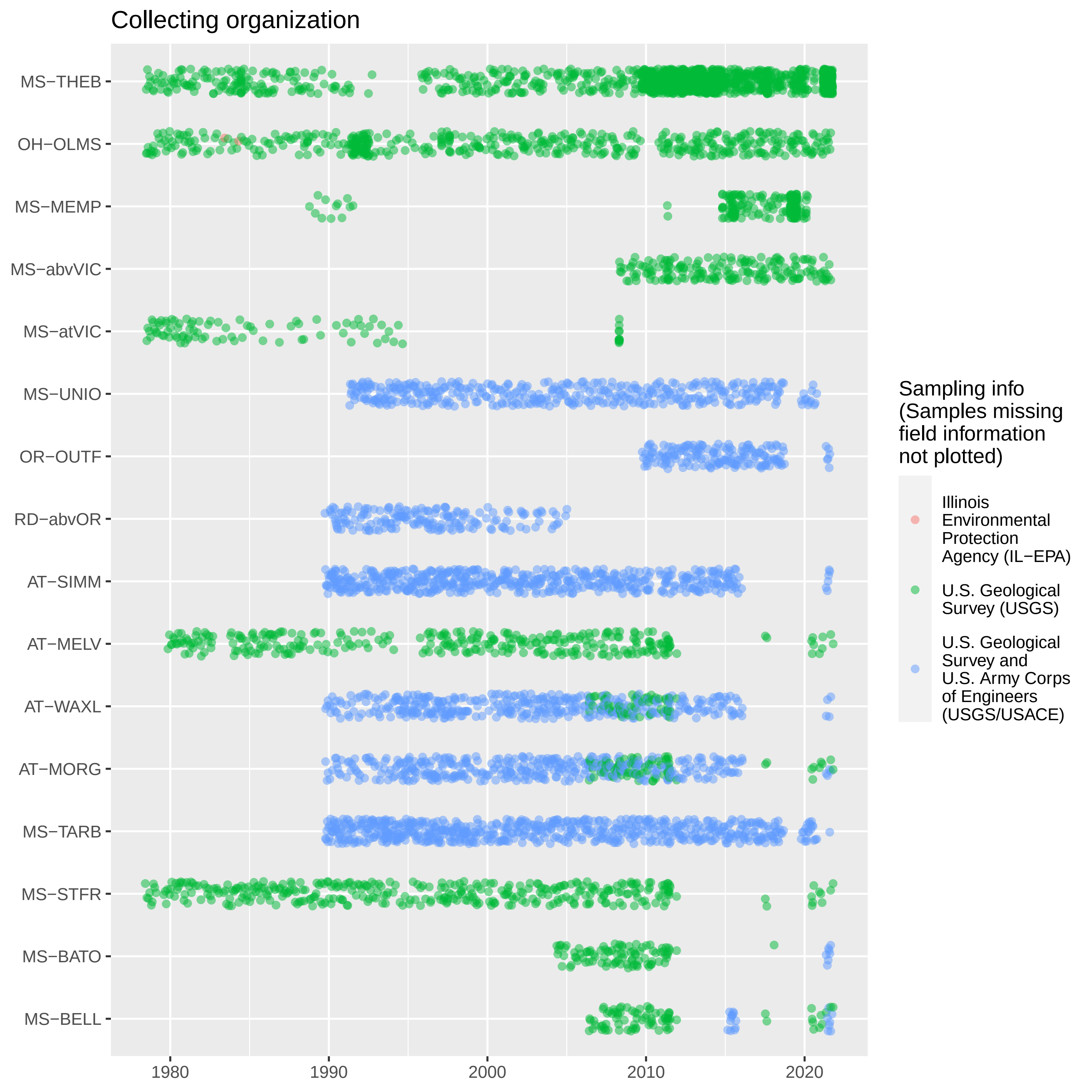
Collecting organization
MS−THEB
OH−OLMS
MS−MEMP
MS−abvVIC
MS−atVIC
MS−UNIO
OR−OUTF
RD−abvOR
AT−SIMM
AT−MELV
AT−WAXL
AT−MORG
MS−TARB
MS−STFR
MS−BATO
MS−BELL
1980
1990
2000
2010
2020
Sampling info (Samples missing field information not plotted)
Illinois Environmental Protection Agency (IL−EPA)
U.S. Geological Survey (USGS)
U.S. Geological Survey and U.S. Army Corps of Engineers (USGS/USACE)

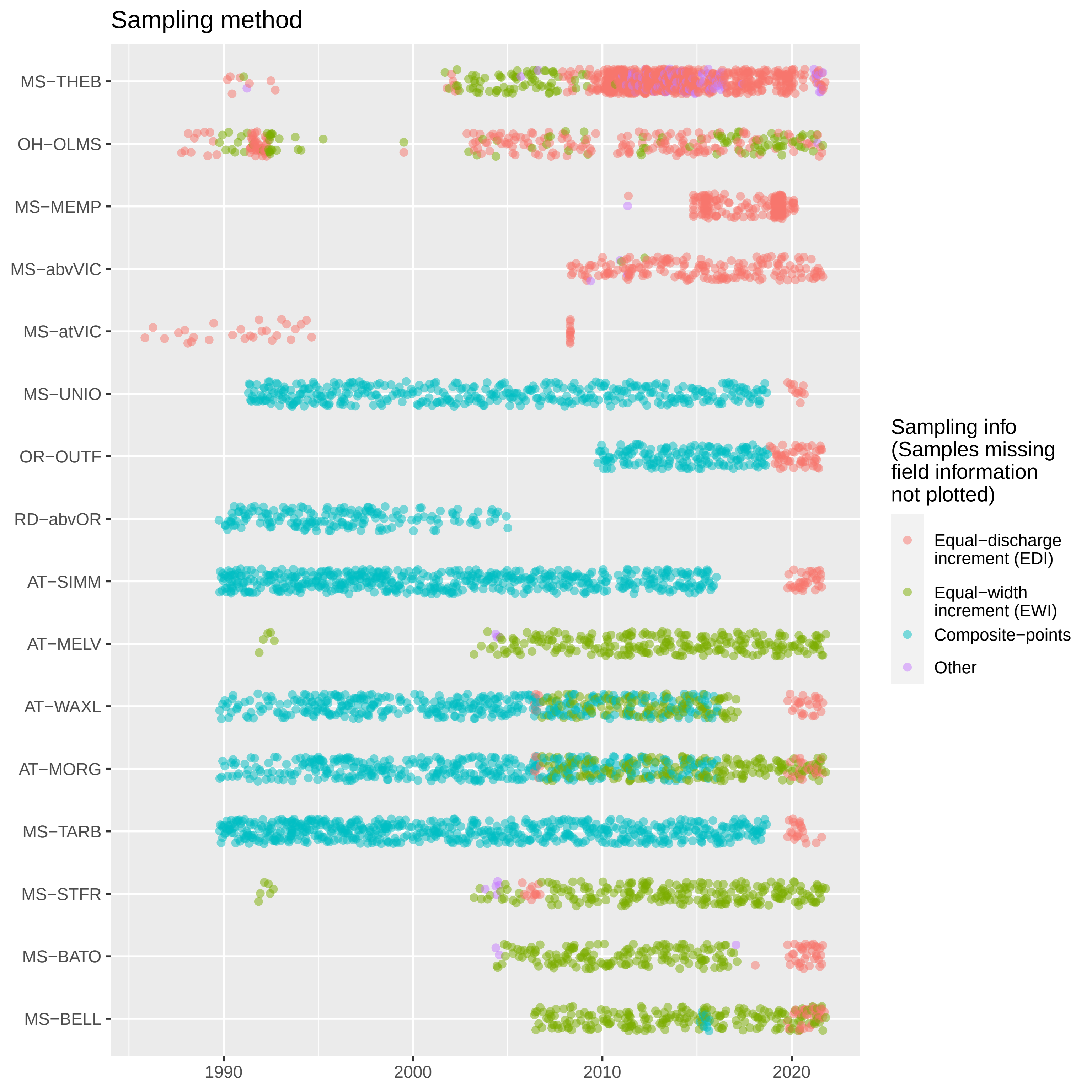

Sampling method
MS−THEB
OH−OLMS
MS−MEMP
MS−abvVIC
MS−atVIC
MS−UNIO
OR−OUTF
RD−abvOR
AT−SIMM
AT−MELV
AT−WAXL
AT−MORG
MS−TARB
MS−STFR
MS−BATO
MS−BELL
1990
2000
2010
2020
Sampling info (Samples missing field information not plotted)
Equal−discharge increment (EDI)
Equal−width increment (EWI)
Composite−points
Other

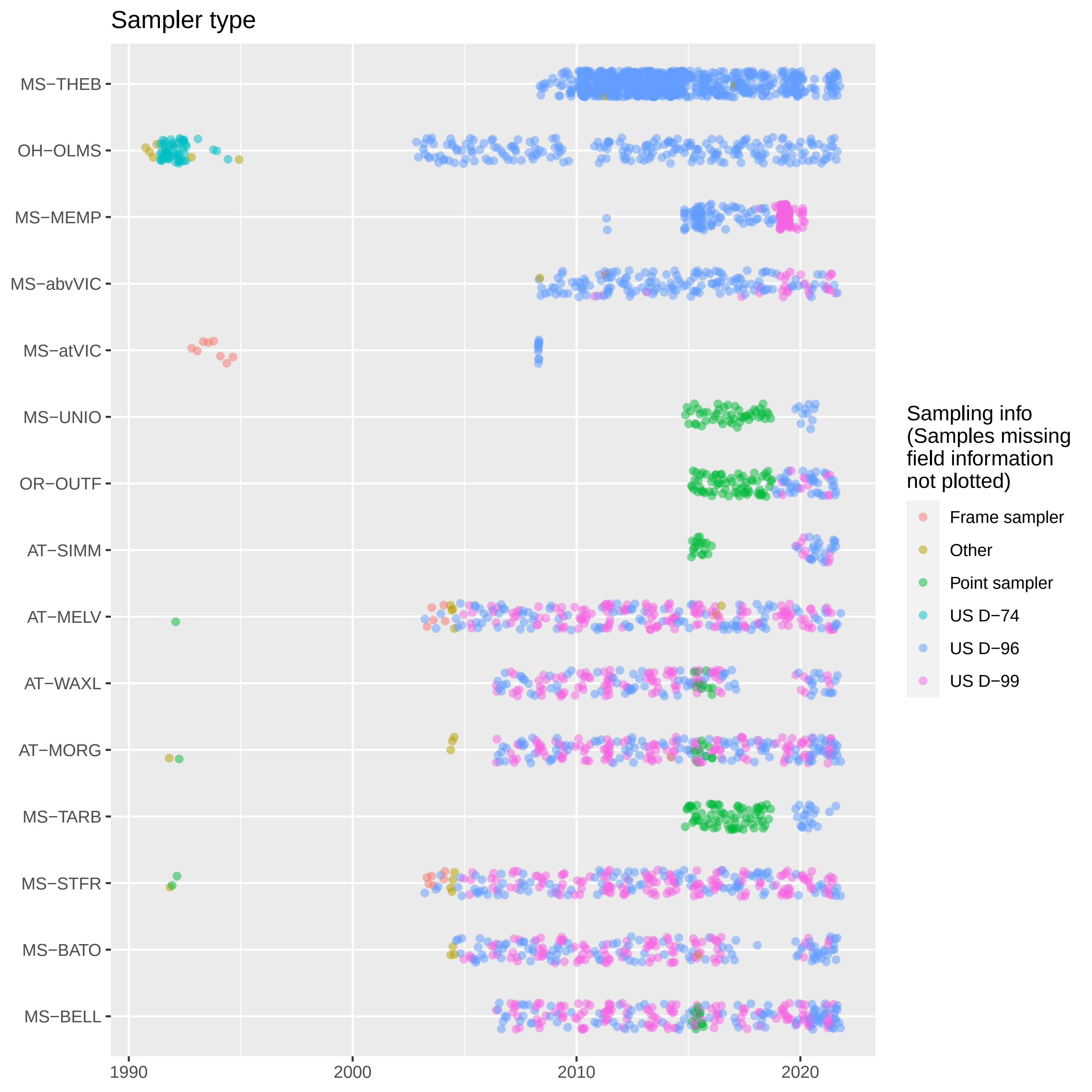

Sampler type
MS−THEB
OH−OLMS
MS−MEMP
MS−abvVIC
MS−atVIC
MS−UNIO
OR−OUTF
AT−SIMM
AT−MELV
AT−WAXL
AT−MORG
MS−TARB
MS−STFR
MS−BATO
MS−BELL
1990
2000
2010
2020
Sampling info (Samples missing field information not plotted)
Frame sampler
Other
Point sampler
US D−74
US D−96
US D−99

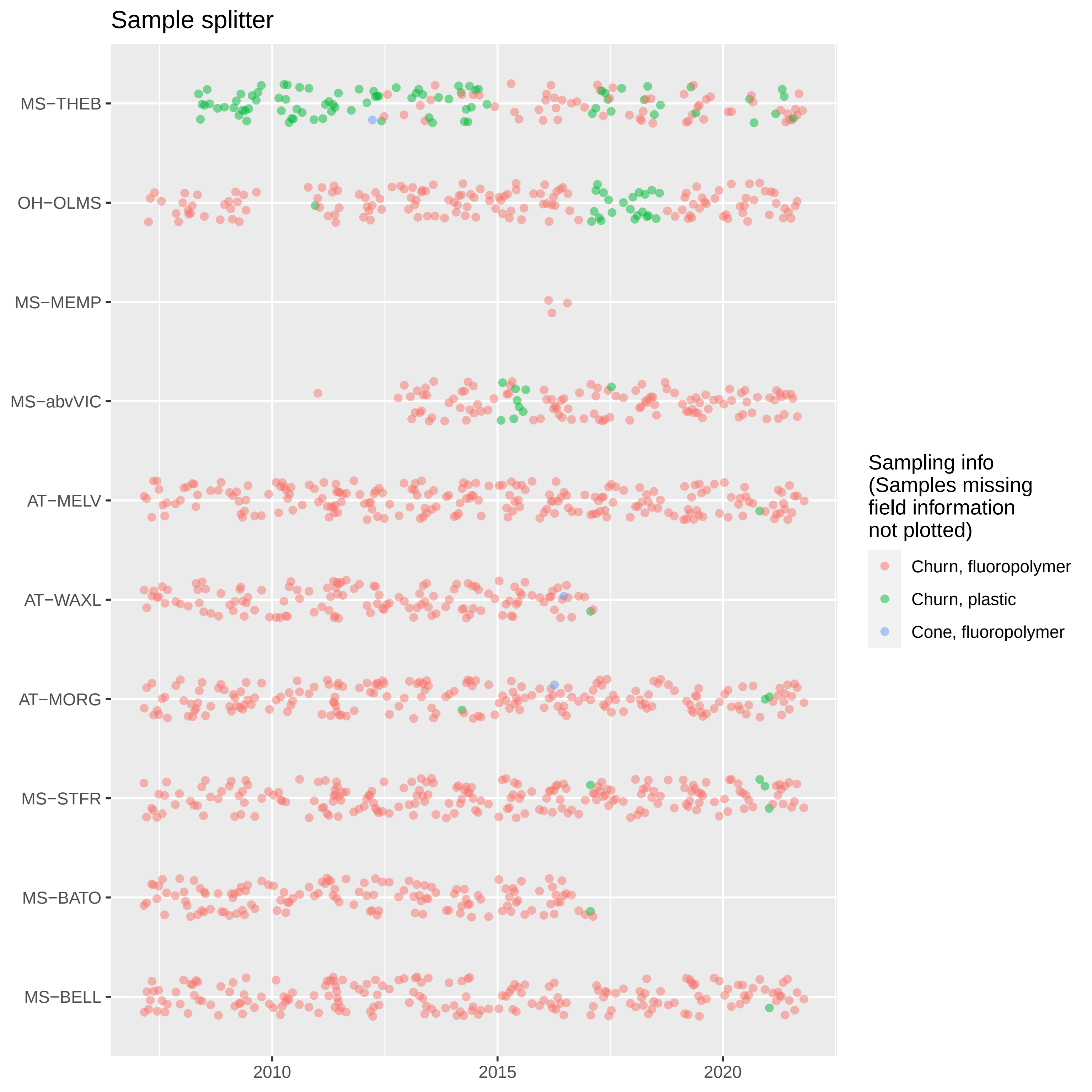
Sample splitter
MS−THEB
OH−OLMS
MS−MEMP
MS−abvVIC
AT−MELV
AT−WAXL
AT−MORG
MS−STFR
MS−BATO
MS−BELL
2010
2015
2020
Sampling info
(Samples missing
field information
not plotted)
Churn, fluoropolymer
Churn, plastic
Cone, fluoropolymer

Nozzle material

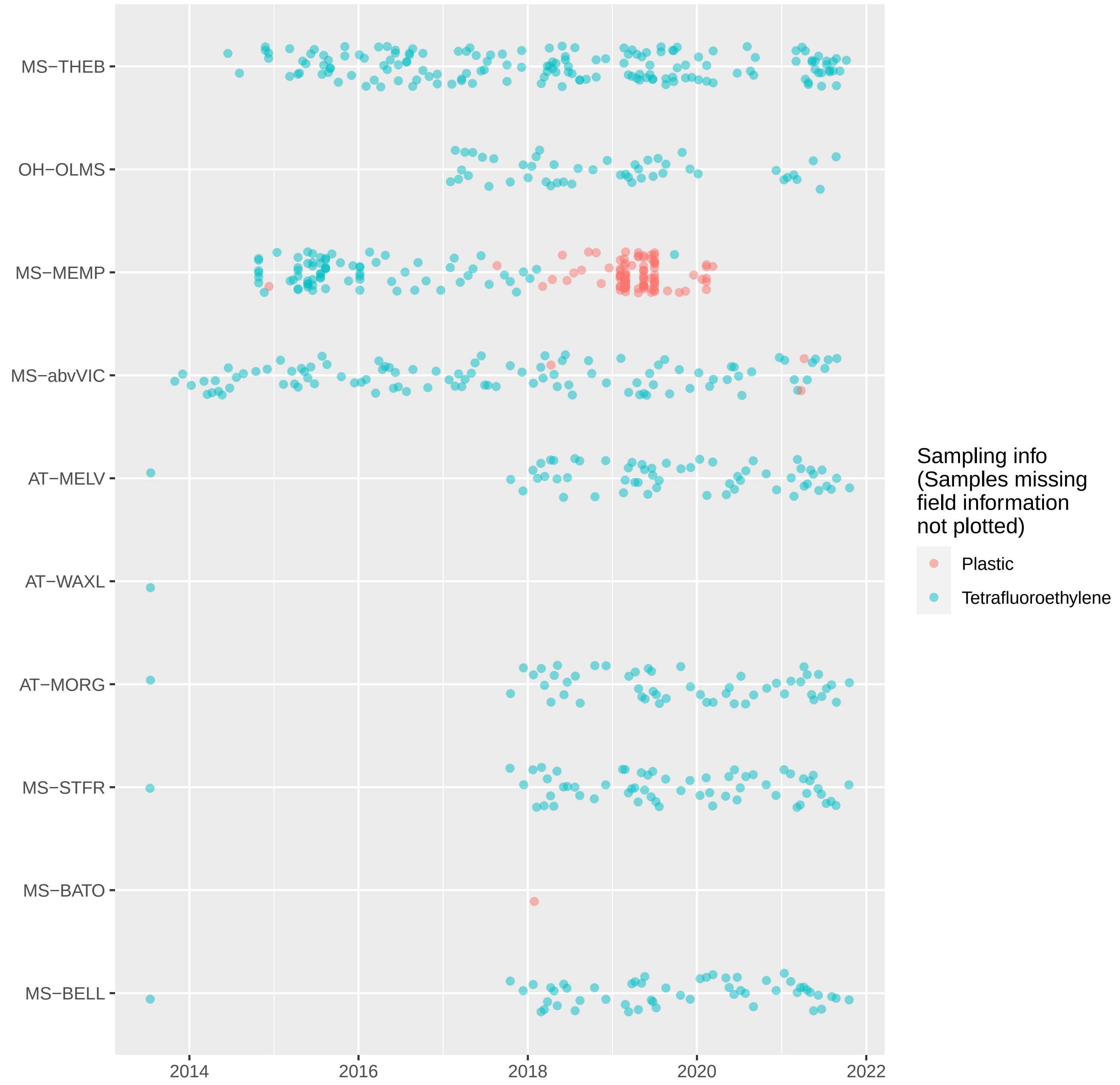

Nozzle diameter

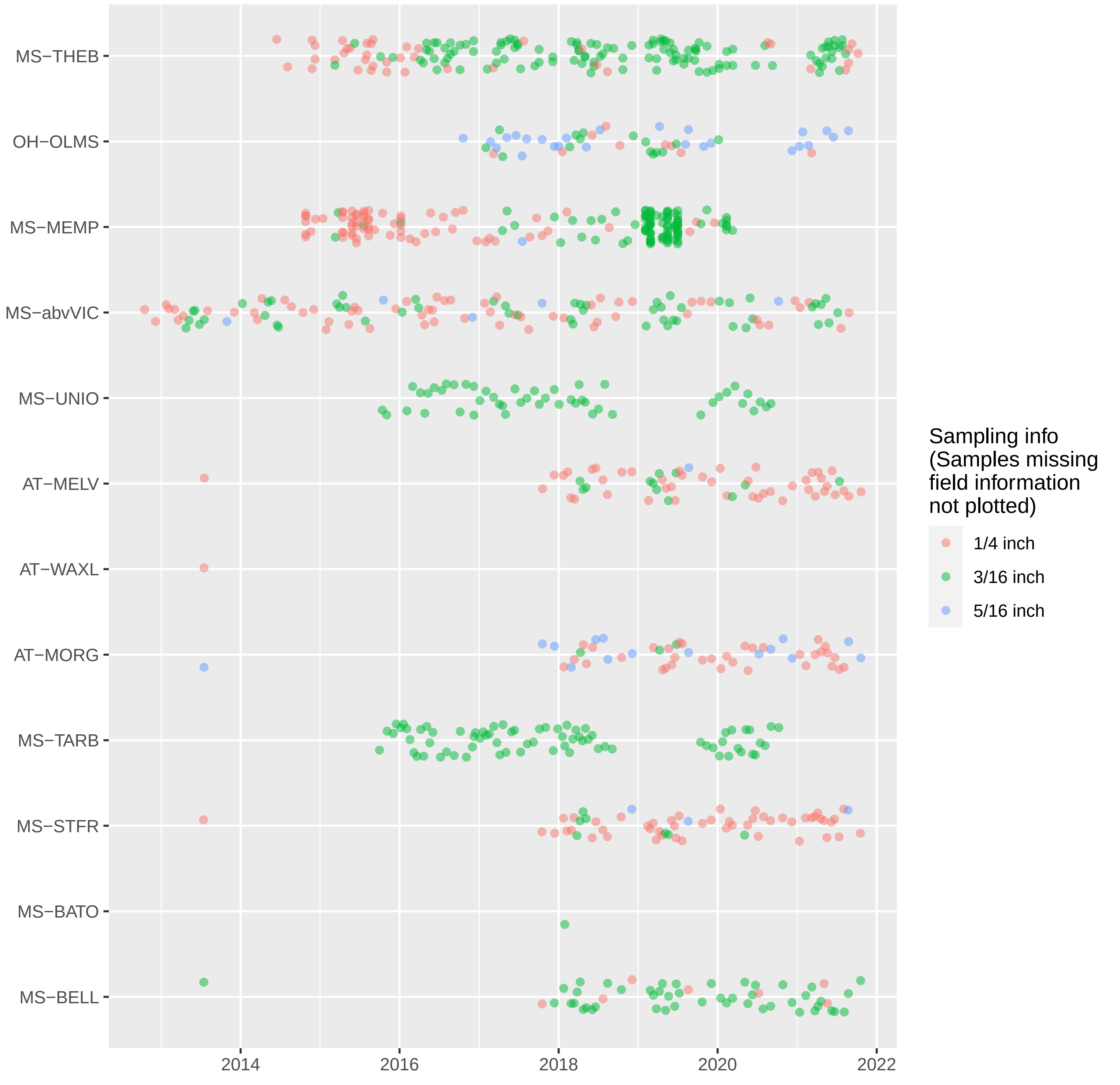

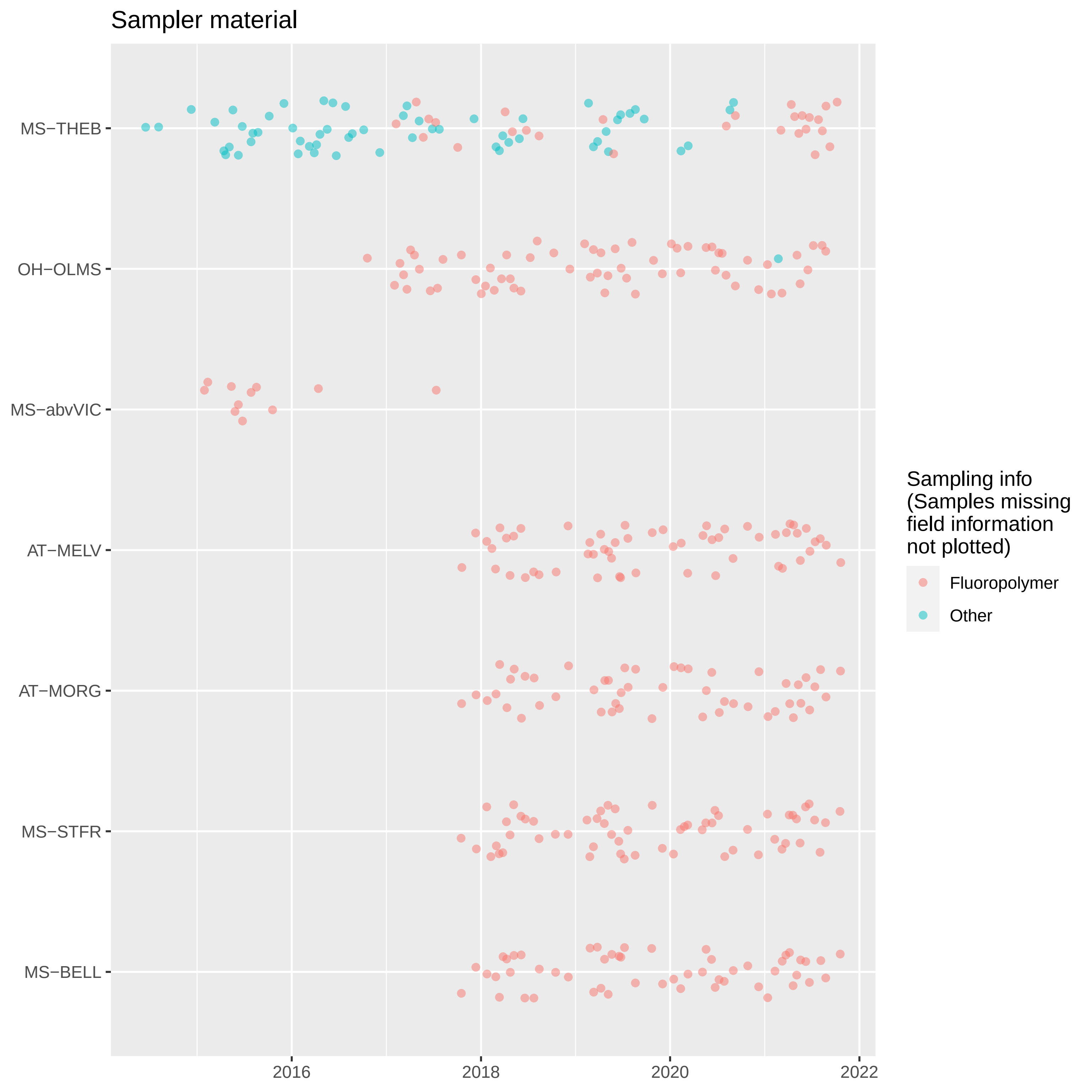

Sampler material
MS−THEB
OH−OLMS
MS−abvVIC
AT−MELV
AT−MORG
MS−STFR
MS−BELL
2016
2018
2020
2022
Sampling info
(Samples missing
field information
not plotted)
Fluoropolymer
Other

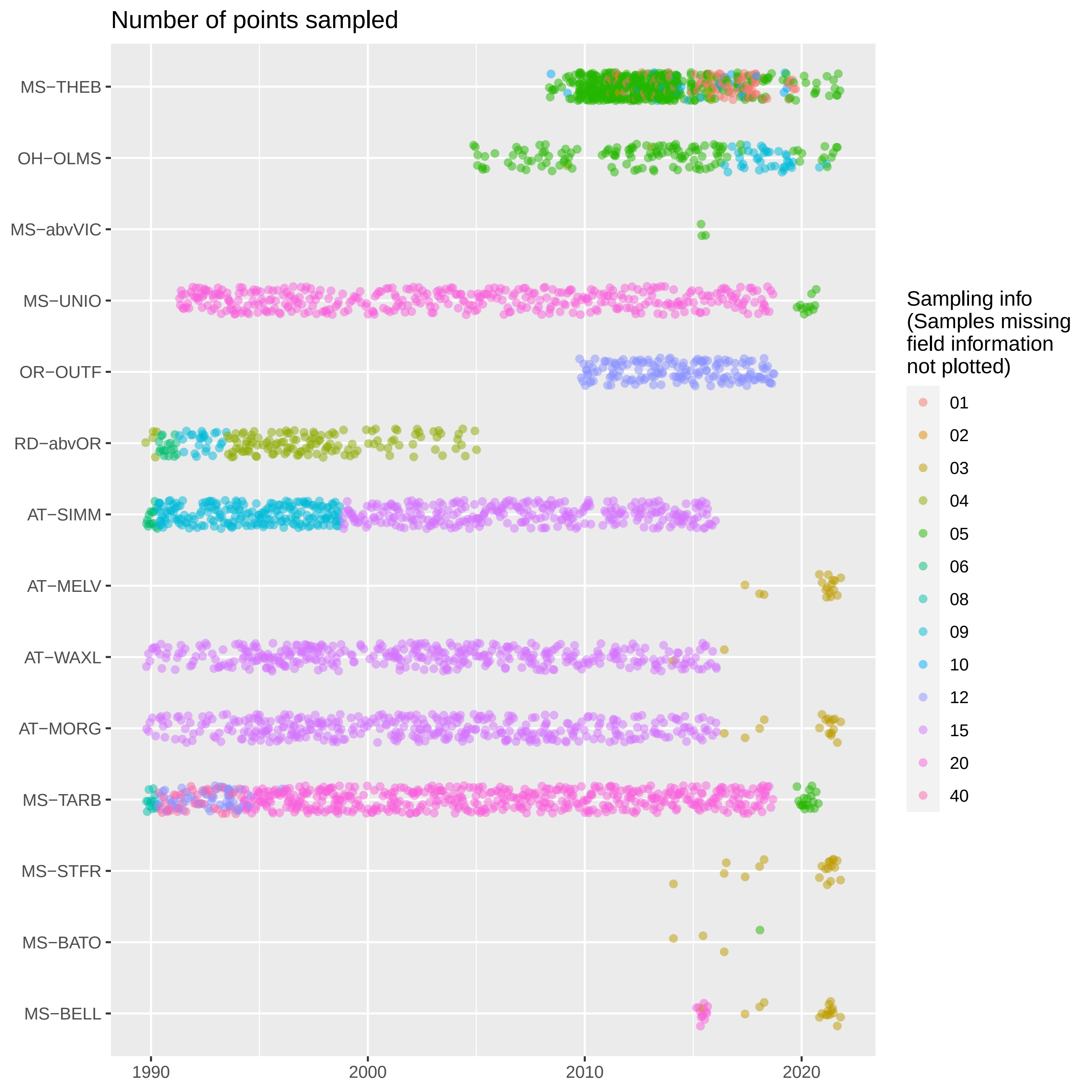

Number of points sampled
MS−THEB
OH−OLMS
MS−abvVIC
MS−UNIO
OR−OUTF
RD−abvOR
AT−SIMM
AT−MELV
AT−WAXL
AT−MORG
MS−TARB
MS−STFR
MS−BATO
MS−BELL
1990
2000
2010
2020
Sampling info (Samples missing field information not plotted)
01
02
03
04
05
06
08
09
10
12
15
20
40
